# Effects of antibiotic resistance, drug target attainment, bacterial pathogenicity and virulence, and antibiotic access and affordability on outcomes in neonatal sepsis: an international microbiology and drug evaluation prospective substudy (BARNARDS)

**DOI:** 10.1016/S1473-3099(21)00050-5

**Published:** 2021-12

**Authors:** Kathryn M Thomson, Calie Dyer, Feiyan Liu, Kirsty Sands, Edward Portal, Maria J Carvalho, Matthew Barrell, Ian Boostrom, Susanna Dunachie, Refath Farzana, Ana Ferreira, Francis Frayne, Brekhna Hassan, Ellis Jones, Lim Jones, Jordan Mathias, Rebecca Milton, Jessica Rees, Grace J Chan, Delayehu Bekele, Abayneh Mahlet, Sulagna Basu, Ranjan K Nandy, Bijan Saha, Kenneth Iregbu, Fatima Modibbo, Stella Uwaezuoke, Rabaab Zahra, Haider Shirazi, Najeeb U Syed, Jean-Baptiste Mazarati, Aniceth Rucogoza, Lucie Gaju, Shaheen Mehtar, Andre N H Bulabula, Andrew Whitelaw, Johan G C van Hasselt, Timothy R Walsh, BARNARDS Group, Samir Saha, Maksuda Islam, Zabed Bin-Ahmed, Wazir Ahmed, Taslima Begum, Mitu Chowdhury, Shaila Sharmin, Chumki Rani Dey, Uttam, Abdul Matin, Sowmitra Ranjan Chakraborty, Sadia Tasmin, Dipa Rema, Rashida Khatun, Liza Nath, Nigatu Balkachew, Delayehu Bekele, Katherine Schaughency, Semaria Solomon, Zenebe Gebreyohanes, Rozina Ambachew, Oludare Odumade, Misgana Haileselassie, Grace Chan, Abigail Russo, Redeat Workneh, Gesit Metaferia, Mahlet Abayneh, Yahya Zekaria Mohammed, Tefera Biteye, Alula Teklu, Wendimagegn Gezahegn, Partha Sarathi Chakravorty, Anuradha Mukherjee, Ranjan Kumar Nandy, Samarpan Roy, Anuradha Sinha, Sharmi Naha, Sukla Saha Malakar, Siddhartha Bose, Monaki Majhi, Subhasree Sahoo, Putul Mukherjee, Sumitra Kumari Routa, Chaitali Nandi, Sulagna Basu, Bijan Saha, Pinaki Chattopadhyay, Fatima Zara Isa Modibbo, Stella Uwaezuoke, Dilichukwu Meduekwe, Khairiyya Muhammad, Queen Nsude, Ifeoma Ukeh, Mary-Joe Okenu, Akpulu Chinenye, Samuel Yakubu, Vivian Asunugwo, Folake Aina, Isibong Issy, Dolapo Adekeye, Adiele Eunice, Abdulmlik Amina, R Oyewole, I Oloton, BC Nnaji, M Umejiego, PN Anoke, S Adebayo, GO Abegunrin, OB Omotosho, R Ibrahim, B Igwe, M Abroko, K Balami, L Bayem, C Anyanwu, H Haruna, J Okike, K Goroh, M Boi-Sunday, Augusta Ugafor, Maryam Makama, Kaniba Ndukwe, Anastesia Odama, Hadiza Yusuf, Patience Wachukwu, Kachalla Yahaya, Titus Kalade Colsons, Mercy Kura, Damilola Orebiyi, Kenneth C. Iregbu, Chukwuemeka Mmadueke, Lamidi Audu, Nura Idris, Safiya Gambo, Jamila Ibrahim, Edwin Precious, Ashiru Hassan, Shamsudden Gwadabe, Adeola Adeleye Falola, Muhammad Aliyu, Amina Ibrahim, Aisha Sani Mukaddas, Rashida Yakubu Khalid, Fatima Ibrahim Alkali, Maryam Yahaya Muhammad, Fatima Mohammad Tukur, Surayya Mustapha Muhammad, Adeola Shittu, Murjanatu Bello, Muhammad Abubakar Hassan, Fatima Habib Sa ad, Aishatu Kassim, Haider Shirazi, Adil Muhammad, Rabaab Zahra, Syed Najeeb Ullah, Muhammad Hilal Jan, Rubina Kamran, Sajana, Jazba Saeed, Noreen Maqsood, Maria Zafar, Saraeen Sadiq, Sumble Ahsan, Madiha Tariq, Sidra Sajid, Hasma Mustafa, Anees-ur Rehman, Atif Muhammad, Gahssan Mehmood, Mahnoor Nisar, Shermeen Akif, Tahira Yasmeen, Sabir Nawaz, Anam Shanal Atta, Mian Laiq-ur-Rehman, Robina Kousar, Kalsoom Bibi, Kosar Waheed, Zainab Majeed, Ayesha Jalil, Espoir Kajibwami, Aniceth Rucogoza, Innocent Nzabahimana, Mazarati Jean-Baptiste, Lucie Gaju, Kankundiye Riziki, Brigette Uwamahoro, Rachel Uwera, Eugenie Nyiratuza, Kumwami Muzungu, Violette Uwitonze, Marie C Horanimpundu, Francine Nzeyimana, Prince Mitima, Angela Dramowski, Andrew Whitelaw, Lauren Paterson, Mary Frans, Marvina Johnson, Eveline Swanepoel, Zoleka Bojana, Mieme du Preez, Shaheen Mehtar, Andre Bulabula, Feiyan Liu, Johan GC van Hasselt, Timothy Walsh, Kirsty Sands, Maria Carvalho, Rebecca Milton, Kathryn Thomson, Edward Portal, Jordan Mathias, Calie Dyer, Ana Ferreira, Robert Andrews, John Watkins, David Gillespie, Kerry Hood, Katie Taiyai, Nigel Kirby, Maria Nieto, Thomas Hender, Patrick Hogan, Habiba Saif, Brekhna Hassan, Ellis Jones, Matthew Barrell, Ian Boostrom, Francis Frayne, Jessica Rees, Lim Jones, Susanna Dunachie, Brad Spiller, Julian Parkhill

**Affiliations:** a Institute of Infection and Immunity, School of Medicine, Cardiff University, Cardiff, UK; b Centre for Trials Research, Cardiff University, Cardiff, UK; c Leiden Academic Centre for Drug Research, Leiden University, Leiden, Netherlands; d Oxford Institute of Antimicrobial Research, Department of Zoology, University of Oxford, Oxford, UK; e Ineos Oxford Institute of Antimicrobial Research, Department of Zoology, University of Oxford, Oxford, UK; f Institute of Biomedicine, Department of Medical Sciences, University of Aveiro, Aveiro, Portugal; g Centre for Tropical Medicine and Global Health, University of Oxford, Oxford, UK; h Mahidol-Oxford Tropical Medicine Research Unit, Mahidol University, Bangkok, Thailand; i Public Health Wales Microbiology, University Hospital of Wales, Cardiff, UK; j Division of Medicine Critical Care, Boston Children's Hospital, Boston, MA, USA; k Department of Epidemiology, Harvard T H Chan School of Public Health, Boston, MA, USA; l Department of Obstetrics and Gynecology, St Paul's Hospital Millennium Medical College, Addis Ababa, Ethiopia; m Department of Paediatrics, St Paul's Hospital Millennium Medical College, Addis Ababa, Ethiopia; n Division of Bacteriology, ICMR-National Institute of Cholera and Enteric Diseases Beliaghata, Kolkata, India; o Department of Neonatology, Institute of Postgraduate Medical Education & Research, Kolkata, India; p National Hospital Abuja, Nigeria; q 54gene, Lagos, Nigeria; r Federal Medical Centre Jabi, Abuja, Nigeria; s Quaid-i-Azam University, Islamabad, Pakistan; t Pakistan Institute of Medical Sciences, Islamabad, Pakistan; u University Teaching Hospital, Kigali, Rwanda; v National Reference Laboratory, Rwanda Biomedical Center, Kigali, Rwanda; w Department of Global Health, Stellenbosch University, Cape Town, South Africa; x Division of Medical Microbiology, Stellenbosch University, Cape Town, South Africa; y National Health Laboratory Service, Tygerberg Hospital, Cape Town, South Africa

## Abstract

**Background:**

Sepsis is a major contributor to neonatal mortality, particularly in low-income and middle-income countries (LMICs). WHO advocates ampicillin–gentamicin as first-line therapy for the management of neonatal sepsis. In the BARNARDS observational cohort study of neonatal sepsis and antimicrobial resistance in LMICs, common sepsis pathogens were characterised via whole genome sequencing (WGS) and antimicrobial resistance profiles. In this substudy of BARNARDS, we aimed to assess the use and efficacy of empirical antibiotic therapies commonly used in LMICs for neonatal sepsis.

**Methods:**

In BARNARDS, consenting mother–neonates aged 0–60 days dyads were enrolled on delivery or neonatal presentation with suspected sepsis at 12 BARNARDS clinical sites in Bangladesh, Ethiopia, India, Pakistan, Nigeria, Rwanda, and South Africa. Stillborn babies were excluded from the study. Blood samples were collected from neonates presenting with clinical signs of sepsis, and WGS and minimum inhibitory concentrations for antibiotic treatment were determined for bacterial isolates from culture-confirmed sepsis. Neonatal outcome data were collected following enrolment until 60 days of life. Antibiotic usage and neonatal outcome data were assessed. Survival analyses were adjusted to take into account potential clinical confounding variables related to the birth and pathogen. Additionally, resistance profiles, pharmacokinetic–pharmacodynamic probability of target attainment, and frequency of resistance (ie, resistance defined by in-vitro growth of isolates when challenged by antibiotics) were assessed. Questionnaires on health structures and antibiotic costs evaluated accessibility and affordability.

**Findings:**

Between Nov 12, 2015, and Feb 1, 2018, 36 285 neonates were enrolled into the main BARNARDS study, of whom 9874 had clinically diagnosed sepsis and 5749 had available antibiotic data. The four most commonly prescribed antibiotic combinations given to 4451 neonates (77·42%) of 5749 were ampicillin–gentamicin, ceftazidime–amikacin, piperacillin–tazobactam–amikacin, and amoxicillin clavulanate–amikacin. This dataset assessed 476 prescriptions for 442 neonates treated with one of these antibiotic combinations with WGS data (all BARNARDS countries were represented in this subset except India). Multiple pathogens were isolated, totalling 457 isolates. Reported mortality was lower for neonates treated with ceftazidime–amikacin than for neonates treated with ampicillin–gentamicin (hazard ratio [adjusted for clinical variables considered potential confounders to outcomes] 0·32, 95% CI 0·14–0·72; p=0·0060). Of 390 Gram-negative isolates, 379 (97·2%) were resistant to ampicillin and 274 (70·3%) were resistant to gentamicin. Susceptibility of Gram-negative isolates to at least one antibiotic in a treatment combination was noted in 111 (28·5%) to ampicillin–gentamicin; 286 (73·3%) to amoxicillin clavulanate–amikacin; 301 (77·2%) to ceftazidime–amikacin; and 312 (80·0%) to piperacillin–tazobactam–amikacin. A probability of target attainment of 80% or more was noted in 26 neonates (33·7% [SD 0·59]) of 78 with ampicillin–gentamicin; 15 (68·0% [3·84]) of 27 with amoxicillin clavulanate–amikacin; 93 (92·7% [0·24]) of 109 with ceftazidime–amikacin; and 70 (85·3% [0·47]) of 76 with piperacillin–tazobactam–amikacin. However, antibiotic and country effects could not be distinguished. Frequency of resistance was recorded most frequently with fosfomycin (in 78 isolates [68·4%] of 114), followed by colistin (55 isolates [57·3%] of 96), and gentamicin (62 isolates [53·0%] of 117). Sites in six of the seven countries (excluding South Africa) stated that the cost of antibiotics would influence treatment of neonatal sepsis.

**Interpretation:**

Our data raise questions about the empirical use of combined ampicillin–gentamicin for neonatal sepsis in LMICs because of its high resistance and high rates of frequency of resistance and low probability of target attainment. Accessibility and affordability need to be considered when advocating antibiotic treatments with variance in economic health structures across LMICs.

**Funding:**

The Bill & Melinda Gates Foundation.

## Introduction

An estimated 2·5 million neonates or infants in the first month of life die each year globally, with sub-Saharan Africa and Asia having the greatest mortality burden.^1^ Neonatal sepsis often presents with varying clinical presentations and non-specific symptoms (such as lethargy and high temperature).^2^, ^3^ In many low-income and middle-income countries (LMICs), where infant mortality is high, laboratory facilities to assess sepsis-causing pathogens and associated antimicrobial resistance are often unavailable.^4^ Antibiotic treatment is therefore often empirical with therapeutic changes based on clinical response, which is difficult to monitor in LMIC settings.^5^

**Research in context**

**Evidence before this study**

We searched the PubMed, Ovid MEDLINE, Scopus, The World Health Organization Library Database, Popline, and ScienceDirect databases for articles published between Jan 1, 2010, and Jan 1, 2021. Searches were limited to English-language articles and reviews were excluded. We searched for the terms “neonatal sepsis” and “neonatal mortality” but not limited to low-income and middle-income countries (LMICs) and linked these descriptions to the following terms: “virulence factors”, which identified three articles, of which one was of relevance and cited in our Article; “bacterial pathogenicity”, which identified 97 articles, none of which covered bacterial sepsis linked to *Escherichia coli*, *Klebsiella pneumoniae*, or *Staphylococcus aureus*; “target attainment”, which identified 128 articles, none of which described the complete drug combinations analysed in our Article; and “economics”, which identified 97 references, eight that were of interest and one of which is cited in our Article. Furthermore, no relevant articles were found when we searched “LMICs” with “average income” and “antibiotic costs”. Despite the global interest in neonatal sepsis and mortality, we found no designed or undertaken prospective studies that combined microbiology, antibiotic kinetics, and health economics in a holistic manner to further understand the antibiotic treatment of neonatal sepsis and mortality.

**Added value of this study**

This study assessed empirical antibiotic treatment in neonates with biological sepsis from Bangladesh, India, Pakistan, Ethiopia, Nigeria, Rwanda, and South Africa. Data showed deviation from ampicillin plus gentamicin in multiple sites. This might be due to an absence of efficacy caused by high levels of resistance and low probability of target attainment found in this study in addition to high frequency of resistance rates for gentamicin. The combination of ceftazidime and amikacin had a higher probability of target attainment and lower prevalence of resistance than ampicillin–gentamicin and lower frequency of resistance rates for amikacin compared with gentamicin. Treatment with ceftazidime–amikacin was associated with reduced reported mortality compared with treatment with ampicillin–gentamicin. The reduction in reported mortality was significant for the subset analysed in this study (476 prescriptions), but was not significant when the effect of country was accounted for in the model, as therapies prescribed were disproportionately specified to a country, making country effects indistinguishable from antibiotic effects. Our health-cost survey showed that ampicillin and gentamicin were the most affordable antibiotics, followed by amikacin and ceftazidime. These antibiotics were available in all participating countries, except for amikacin which was available in all countries excluding Ethiopia. Multiple antibiotics were not available in a range of countries and varied greatly in price. Participating sites in Bangladesh, India, Pakistan, Ethiopia, Nigeria, and Rwanda stated that the cost of more potent antibiotics is deferred to the patient. The average income of patients within the study was examined and, comparative to the cost of antibiotics when the cost was deferred to the patient, this will often make these antibiotics unaffordable. However, investigating the effect of cost on use of antibiotics was outside the remit of this study.

**Implications of all the available evidence**

Our study indicates that for neonatal sepsis the combination treatment of ampicillin–gentamicin needs to be reviewed for LMICs, and replaced with another combination, potentially ceftazidime–amikacin. As the frequency of resistance was high for fosfomycin, we suggest that this antibiotic is not considered for treatment of neonatal sepsis, singularly or in combination with other antibiotics, although further work is needed here. Data collected on antibiotic costs in LMICs are of concern because costs varied widely between antibiotics and between sites. The cost of an antibiotic inevitably affects therapy choice and invariably the outcome, particularly in Bangladesh, Nigeria, and Ethiopia. Selection of front-line antimicrobial regimes needs to account for the rise in antimicrobial resistance against broad-spectrum antibiotics, while balancing the need for effective therapy, cost, availability, dosing regimens, and potential toxicity.

Our data have immediate implications at every level of health care and suggest that WHO might need to revise their antibiotic guidelines for neonatal sepsis within LMICs, where antibiotic resistance to currently recommended treatments is extremely high. However, survival for neonates treated with ampicillin–gentamicin was higher than expected despite the high resistance and low probability of target attainment, although we believe that this result was due to under-reported mortality. Further work needs to be carried out to understand this discrepancy.

WHO recommends ampicillin in combination with gentamicin for the management of clinical neonatal sepsis;^6^ however, there are persistent concerns regarding antimicrobial resistance and therapeutic failures with this treatment.^7^ Additionally, suboptimal microbiological support in LMICs has led to the empirical use of broad-spectrum β-lactams, aminoglycosides, and even carbapenems.^8^ Antibiotic cost is not always covered by a hospital, state, or government, and might defer to patients, or only certain antibiotics might be covered, affecting neonatal outcomes when alternative treatments are unaffordable.

Access to effective antibiotics needs to be improved,^9^ alongside preventing overuse of antibiotics. To reduce the use of certain antibiotics, the WHO Essential Medicines List for Children^10^ has classified antibiotics into three categories (Access, Watch, and Reserve). The Access group should account for more than 60% of antibiotic usage, providing adequate coverage for most common infections. The Watch group covers antibiotics with a broader spectrum and higher resistance potential. The Reserve group is for last-resort antibiotics targeting multidrug-resistant infections.^10^ Although use of Reserve antibiotics remains low, use of alternative antibiotic regimes have been reported in various countries for treatment of neonatal sepsis.^9^ This might represent an absence of antibiotic stewardship, or necessity because of ineffectiveness of antibiotics in the Access group.

WHO's global action plan highlighted a need for antimicrobial resistance surveillance networks and centres to create and strengthen coordinated regional and global surveillance.^11^, ^12^ In 2015, a Bill & Melinda Gates Foundation-funded study titled Burden of Antibiotic Resistance in Neonates from Developing Societies (BARNARDS) was established^13^ to assess the burden of neonatal sepsis and antimicrobial resistance in LMICs. The objectives of the main BARNARDS study included characterisation of common sepsis pathogens via whole genome sequencing (WGS), antimicrobial resistance profiles, assessment of carriage rates of antimicrobial resistance genes in the normal flora of mothers and neonates older than 7 days, and risk factors associated with neonatal sepsis. In this substudy of BARNARDS, we focused on the effectiveness of antibiotic therapies after determining a high prevalence of pathogens resistant to ampicillin and gentamicin.^14^ This Article describes the use of antibiotics in BARNARDS clinical sites, the effectiveness of recommended empirical treatment for neonatal sepsis, and examines potential alternative treatments. On the basis of resistance data collated in the main BARNARDS study, we hypothesised that neonates treated with ampicillin–gentamicin would have higher reported mortality than those treated with alternative combinations with a lower prevalence of resistance.

## Methods

### Study design

BARNARDS was an observational cohort study analysing sepsis rates in neonates and infants aged 0–60 days, both inborn (ie, born in the participating hospital) or those with clinical presentation of suspected sepsis (non-inborn cohort) in hospitals in LMICs between Nov 12, 2015, and Feb 2, 2018. Herein, the term neonate refers to 0–60 days of life. Countries with participating sites included Bangladesh, Ethiopia, India, Nigeria, Pakistan, Rwanda, and South Africa, with a total of 12 clinical sites.^13^ Ethical approval was obtained locally for each clinical site, granted via local ethics committees (appendix p 1). All mothers were enrolled alongside their neonates at clinical sites, either upon delivery (inborn cohort) or at clinical presentation of suspected sepsis (non-inborn cohort) and questionnaires were completed that included information on the birth of the neonate enrolled. All mothers provided informed consent (forms written in local languages; appendix p 1) and could withdraw from the study at any time.^13^ Stillborn babies were excluded from the study.

Blood samples were obtained from neonates presenting with clinical signs of sepsis according to phlebotomy checklists and microbiology protocols carried out at sites (appendix p 1). Bacterial isolates from positive blood cultures confirming sepsis were stored on charcoal swabs before being sent to Cardiff University (Cardiff, UK) under UN3373 transport regulations, where WGS was done and minimum inhibitory concentrations (MICs) were calculated. Neonates diagnosed with sepsis without a positive blood culture were categorised as clinically diagnosed, as were positive cultures that were deemed as contaminants. Antibiotic treatments were recorded on site. Neonatal outcome data were collected following enrolment until 60 days of life (appendix, p 1).^15^ Neonates of parents who could not be contacted were considered as alive until their age at last observation.

BARNARDS neonates included in this study had available antibiotic information and pathogen WGS data. We collated antibiotic consumption data from all clinical sites and selected the four most common antibiotic combinations for analysis. The study size was based on the maximum number of cases treated with common antibiotic combinations found following retrospective clinical note review, with available WGS data. All neonates from this subset reported as deceased within 60 days of life were included in analyses.

### Antibiotic susceptibility profiling

Agar dilution was done to determine MICs for 14 and 20 antibiotics against Gram-positive and Gram-negative sepsis isolates, respectively, validated against control strains and analysed using European Committee on Antimicrobial Susceptibility Testing (EUCAST) breakpoints (version 9.0, 2019)^16^ (appendix pp 2–3). Coverage was determined as the proportion of isolates susceptible to at least one antibiotic in a combination. Isolates with MIC values determined as requiring increased exposure to an antibiotic were combined with resistant isolates. Additional analyses compared outcomes with coverage for neonates treated with an antibiotic combination prescribed with no treatment change following initial therapy. A scoping literature review was done to obtain a snapshot of global rates of resistance against ampicillin, gentamicin, ceftazidime, and amikacin (appendix p 3).

### Pharmacokinetic and pharmacodynamic modelling

Pharmacokinetic and pharmacodynamic analyses determined probability of target attainment values accounting for differences in dosing schedules, patient-specific characteristics, and MIC values of pathogens for neonates who received an antibiotic combination with no change in treatment. This was repeated for neonates who received additional antibiotic treatments to compare country-specific differences in dosing schedules. The full analysis method is in the appendix (pp 4–6). Briefly, individual patient characteristics were derived using reference values^17^ for bodyweight, serum creatinine, and albumin, and applied for probability of target attainment simulations^18^ using published population pharmacokinetic models for each antibiotic combination.^11^ Target attainment was met for a combination when at least one antibiotic reached their MIC-specific pharmacokinetic and pharmacodynamic target value associated with efficacy. Simulated probability of target attainment values for each combination were compared with MIC distributions and a probability of target attainment of 80% or more after 100 simulations and compared with observed survival. Additional probability of target attainment simulations were done for meropenem, fosfomycin, and colistin.

### Frequency of resistance

Frequency of resistance (ie, resistance defined by in-vitro growth of isolates when challenged by antibiotics) was assessed for antibiotics commonly used in the treatment of neonatal sepsis at BARNARDS sites and potential antibiotic alternatives with lower rates of resistance. Frequency of resistance isolates were based on different bacterial species, sequence types, outcomes, and resistance profiles from across clinical sites selected proportionally from the BARNARDS dataset but restrained to isolates recorded as sensitive during MICs separately for each antibiotic (appendix p 7). Frequency of resistance rates were calculated as the proportion of isolates needed to develop growth and colony forming units per mL.^19^

### Virulence factors and pathogenicity indexing

Similar to criteria outlined for frequency of resistance isolate selection, virulence factors and pathogenicity indexing were also undertaken for a selection of isolates on the basis of varied bacterial species, sequence types, outcomes, and resistance profiles from a range of BARNARDS sites; however, clinical outcome was also considered (appendix p 7). *Galleria mellonella* models were used to determine bacterial pathogenicity indexes as previously described.^20^ In this model, bacterial isolates were grown in broth, centrifuged, washed, and resuspended in saline solution and injected at certain dilutions into *Galleria* larvae (10^−7^ to 10^−8^ dilutions for *Enterobacteriaceae*; 10^−7^ to 10^−9^ for *Staphylococcus aureus*). Ten larvae were used for each dilution per isolate in triplicate with controls used as previously outlined.^20^ *Galleria* were kept at 37°C and mortality recorded at 24, 48, and 72 h. Pathogenicity indexes were calculated from the proportion and time of larvae death from a bacterial dilution.

WGS and bioinformatics analyses were undertaken in the main BARNARDS study,^14^ (accession numbers: PRJEB33565 [Gram-negative isolates] and PRJEB40908 [Gram-positive isolates]). Individual accession numbers are in the appendix (pp 8–10). *Escherichia coli, Klebsiella pneumoniae,* and *S aureus* isolates selected for pathogenicity indexing were analysed for the presence of virulence factors using Abricate (v 0.9.7) with the virulence factors database with a cutoff of 98% or more gene identity.^21^, ^22^ A presence or absence matrix of virulence-associated factors deposited in the database was created to generate a total virulence score (appendix p 7).^22^

### Antibiotic costs and availability

To better understand neonatal sepsis treatment practices, questionnaires were sent and completed by the principal investigators at each BARNARDS clinical site. The questionnaire covered antibiotic availability, national public or private health partnerships, and antibiotic costs (ie, whether they were paid for by the hospital, state, government, or the patients). Data for standard dosing regimens were collected for clinical sites (appendix pp 11–12).

### Statistical analysis

Cox regression hazard ratios (HRs) were calculated in R v 4.0.2,^23^ using the Survival (v3.2.7),^24^ coxphf (v 1.13.1), and coxme (v 2.2.16)^25^ packages. Potential clinical confounding variables that affected these models were included in the analyses (detailed in the appendix p 14); HRs shown have been adjusted for these potential confounding variables. Proportional hazard assumptions were assessed via Schoenfeld residuals showing no patterns over time, unless otherwise stated. Survminer (version 0.4.8) and ggfortify (version 0.4.11) packages were used to create survival plots. Virulence factors and pathogenicity indexing *R*^2^ correlations were done with Microsoft Excel. Normality and Mann-Whitney *U* tests were run in IBM SPSS version 25 for associations between virulence factors or pathogenicity index and outcome, with sensitivity analyses undertaken (appendix p 13). Frequency of resistance results were log-transformed with an added standard of 1 × 10^−10^ to incorporate zero values into violin plots, created in R (version 3.6.1) using the ggplot2 (version 3.3.2) package.^23^, ^26^ Pharmacokinetic–pharmacodynamic analysis was done in R (version 3.5.1)^23^ with the packages RxODE (version 0.9.1.1), ggplot 2 (version 3.3.0),^26^ and dplyr (version 0.8.5).

### Role of the funding source

The funder of the study had no role in study design, data collection, data analysis, data interpretation, or writing of the report.

## Results

**Figure 1:**
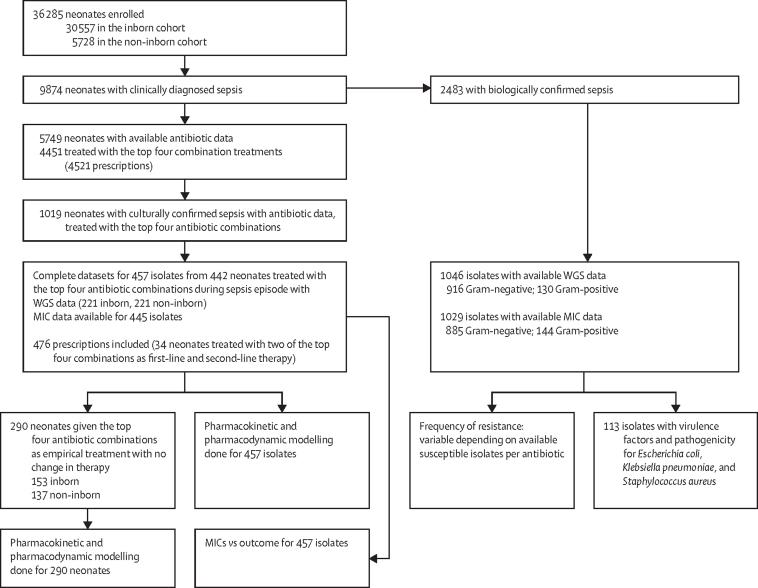
Study profile The diagram shows the process of isolate selection for inclusion in this study, as a substudy from the main BARNARDS project and subsets used for analyses. BARNARDS=Burden of Antibiotic Resistance in Neonates from Developing Societies. WGS=whole genome sequencing. MIC=minimum inhibitory concentration.

Between Nov 12, 2015, and Feb 1, 2018, 36 285 neonates were enrolled onto the main BARNARDS project, of whom 9874 had clinically diagnosed sepsis, 5749 had antibiotic data available (figure 1), and 2483 were confirmed with a positive blood culture. The four most common combinations of first-line antibiotics prescribed were given to 4451 neonates (77·4%) of 5749: ampicillin–gentamicin administered by almost all sites across Africa and in Bangladesh; ceftazidime–amikacin used primarily by sites in Bangladesh with minor usage in Nigeria and Pakistan; piperacillin–tazobactam and amikacin (piperacillin–tazobactam–amikacin) used by sites in Pakistan and occasionally in South Africa and India; and amoxicillin clavulanate and amikacin (amoxicillin clavulanate–amikacin) used by sites in Nigeria (appendix p 14). We therefore assessed neonates treated with these four combinations in this study. Antibiotic prescription data was available for 1019 of 2483 culturally confirmed sepsis cases. 300 of these isolates were not analysed further because of study constraints, lost viability, or they were deemed contaminants. Furthermore, 262 isolates were not related to treatment of the neonate with one of the top four antibiotic combinations. Therefore, this subset is based on 457 isolates from 442 neonates who were treated with one of the four common antibiotic combinations described above, for which WGS data were available. Of the isolates with WGS data, 12 had undetermined MIC results. However, these neonates were kept in the subset as they were valuable in contributing to other analyses. Multiple pathogens were isolated from 442 neonates, totalling 457 isolates (figure 1). Two of the above antibiotic combinations were prescribed as first-line and second-line therapy to 34 neonates; both combinations were assessed, totalling 476 relevant prescriptions. Inborn and non-inborn neonates were included, with varied clinical presentations (appendix pp 14–15). From the 442 neonates included, 290 were treated with a single antibiotic combination only, with no change in therapy (figure 1).

BARNARDS countries were represented in this subset, except for India. However, antibiotic combinations were dominated by certain sites (appendix p 14). The proportion of neonates reported deceased in this subset was similar to those with confirmed sepsis overall from BARNARDS (*X*^2^[1] = 1·18, p=0·28).

**Figure 2:**
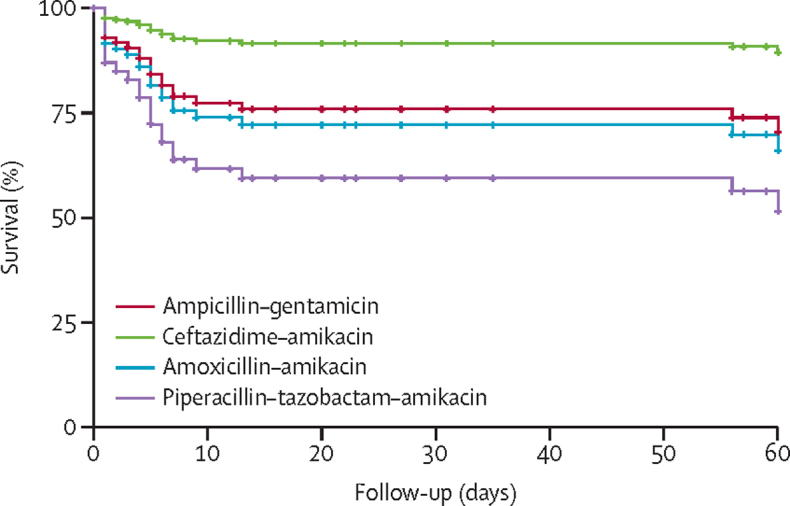
Survival analysis for neonates treated with each antibiotic therapy Analysis was done for 476 neonates through Cox regression hazard ratios, adjusted for clinical factors. Clinical variables considered included cohort, sex, type of pathogen (ie, Gram-negative *vs* Gram-positive); whether the neonate was delivered via caesarean section; and whether the neonate was premature (appendix, pp 14–15) and stratified for onset of sepsis (early-onset sepsis or late-onset sepsis) to meet proportional hazard assumptions. For the purpose of this survival curve, the following clinical variables were set: male sex, inborn cohort, early-onset sepsis, Gram-negative sepsis pathogen type, no caesarean section, and no premature neonates.

Neonates treated with ceftazidime–amikacin had the lowest numerical reported mortality (16 [9·3%] of 172), followed by ampicillin–gentamicin (18 [16·2%] of 111), amoxicillin clavulanate–amikacin (19 [24·4%] of 78), and piperacillin–tazobactam–amikacin (32 [27·8%] of 115; appendix p 15). Neonates treated with ceftazidime–amikacin had significantly reduced reported mortality compared with those treated with ampicillin–gentamicin (adjusted HR 0·32, 95% CI 0·14–0·72; p=0·0060; figure 2; appendix pp 16–18). No differences for reported mortality compared with ampicillin–gentamicin were found for neonates treated with amoxicillin clavulanate–amikacin (HR 1·19, 95% CI 0·60–2·24; p=0·62) or piperacillin–tazobactam–amikacin (1·89, 0·88–4·06; p=0·10; figure 2). Significant results were negated in the mixed model with country incorporated as a random effect, although the data did not fit this model well because of the use of different antibiotics per country (appendix pp 16–18).

Analyses were repeated for 290 neonates treated with one antibiotic combination with no changes after initial treatment. No significant difference was found for this subset between ampicillin–gentamicin and ceftazidime–amikacin (adjusted HR 0·51, 95% CI 0·16–1·69, p=0·27) or piperacillin–tazobactam–amikacin (3·02, 0·93–9·81, p=0·066) although higher mortality was associated with amoxicillin clavulanate–amikacin compared with ampicillin–gentamicin (5·56, 1·71–18·08; p=0·0044; appendix pp 19–21). A high proportion of neonates treated with ampicillin–gentamicin who were not reported as deceased were followed up for fewer than 10 days (55 [59·1%] of 93 compared with 37 [23·7%] of 156 not reported as deceased and treated with ceftazidime–amikacin).

**Figure 3:**
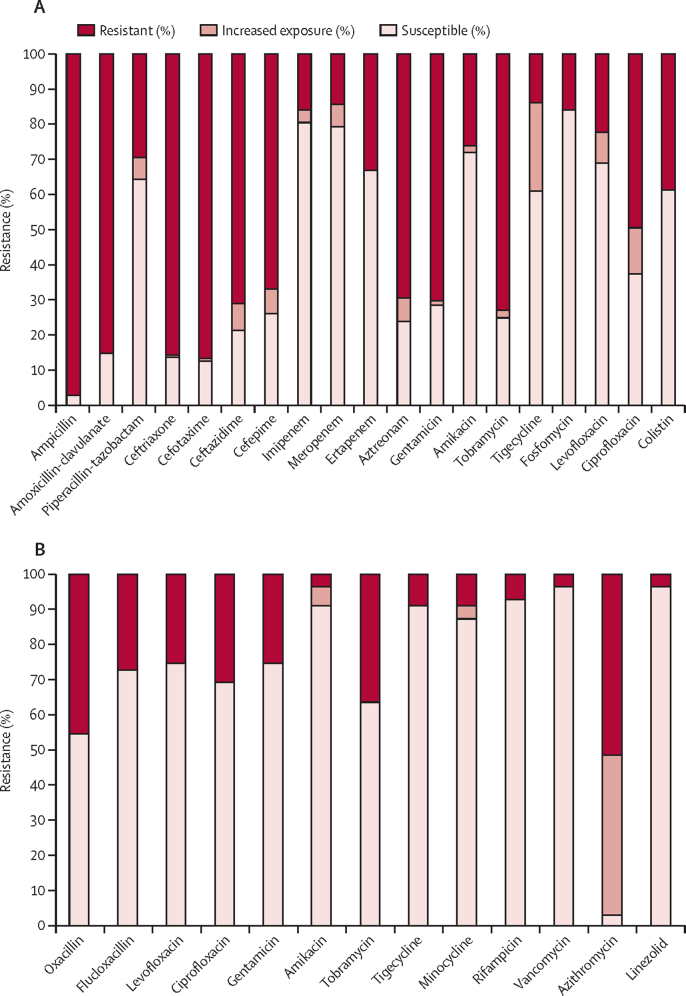
Antibiotic resistance profiles according to EUCAST version 9.0 (2019) (A) 390 Gram-negative isolates were tested against 20 antibiotics (minocycline is not shown as there is no defined breakpoint in EUCAST for Gram-negative species). (B) 55 Gram-positive isolates (33 for azithromycin) were tested against a panel of 14 antibiotics. Resistance profiles for 13 of 14 antibiotics tested against Gram-positive bacteria are shown (ampicillin is not shown as there is no defined breakpoint in EUCAST for *Staphylococcus aureus* against ampicillin, because most *Staphylococci* are penicillinase producers making them resistant to ampicillin).^16^ Breakpoints for oxacillin and flucloxacillin are based on the assumption that isolates with MIC >2 mg/L are resistant because they carry *mecA* or *mecC*. These were also evaluated as meticillin-resistant *S aureus* (45·5% of isolates). MIC_50_ and MIC_90_ results are in the appendix, pp 7–8. EUCAST=European Committee on Antimicrobial Susceptibility Testing. MIC=minimum inhibitory concentration.

Gram-negative isolates were overwhelmingly resistant to ampicillin (379 [97·2%] of 390) and to gentamicin (274 [70·3%] of 390; figure 3; appendix p 22), with similar resistance results noted in sites across both African and south Asian continents (appendix pp 23–24). Amikacin had lower resistance (101 [25·9%] of 390) than other aminoglycosides, in keeping with global comparative data (appendix p 25). Few of the 390 Gram-negative isolates were resistant to fosfomycin (61 [15·6%]), imipenem (62 [15·9%]), and meropenem (56 [14·4%]). Regarding treatment combinations for the 390 Gram-negative isolates, the lowest coverage was provided by ampicillin–gentamicin (111 [28·5%]), with higher coverage from amoxicillin clavulanate–amikacin (286 [73·3%]), ceftazidime–amikacin (301 77·2%]) and piperacillin–tazobactam–amikacin (312 [80·0%]). Gram-positive isolates generally had reduced levels of resistance (figure 3; appendix pp 22, 24). Resistance rates varied between species, with high levels of resistance in *K pneumoniae* and *Acinetobacter baumannii* isolates (appendix pp 25–28).

MIC values were compared with reported outcomes for 290 neonates with no change in therapy (figure 1). Pathogen susceptibility to at least one antibiotic or resistance to both antibiotics received by the neonate did not dictate outcome across treatment combinations tested (ampicillin–gentamicin: HR 1·28, 95% CI 0·25–6·60, p=0·77; ceftazidime–amikacin: 2·78, 0·35–22·24, p=0·34**;** amoxicillin clavulanate–amikacin: 0·37, 0·08–1·67, p=0·20; and piperacillin–tazobactam–amikacin 1·73, 0·38–7·83, p=0·48). Unadjusted results provided due violations of proportional hazard assumptions in related adjusted models (appendix, pp 31–38).

**Figure 4:**
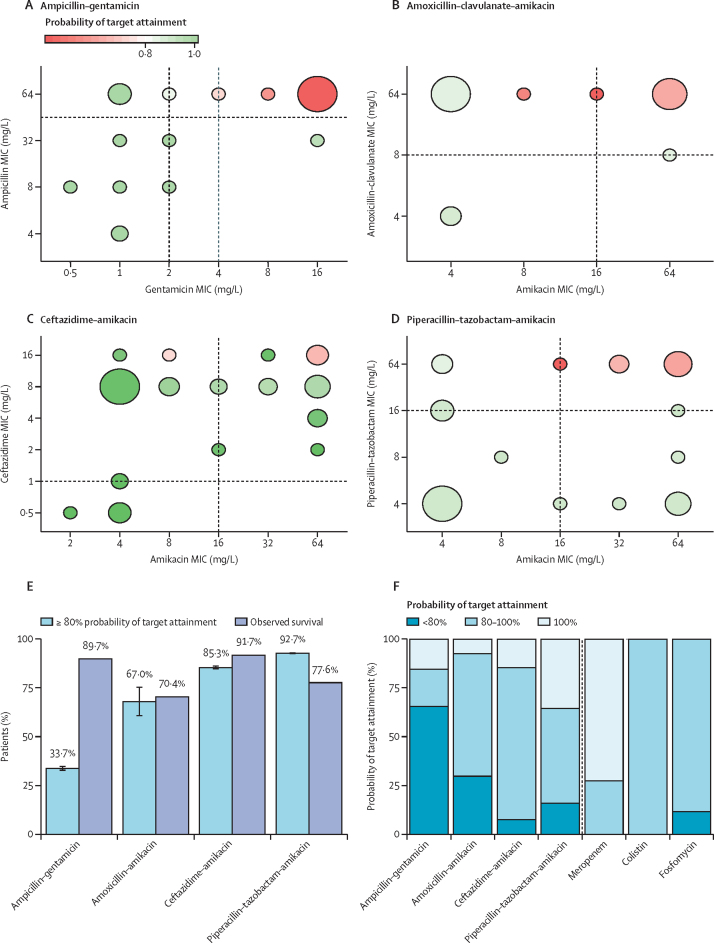
Probability of target attainment of commonly used antibiotic combination treatments (A–D) Relationship between simulated probability of target attainment values and MIC values for four antibiotic combination therapies for 290 neonates treated empirically with no following change in treatment. Vertical and horizontal lines represent ranges of MIC breakpoints according to EUCAST. Size of the bubbles indicates the frequency of isolates with associated MICs. (E) Comparison of simulated probability of target attainment values ≥80% (mean [1·96 SD]) and observed survival rate. (F) Simulated probability of target attainment values for the four combination therapies, compared with meropenem (10mg/kg every 8 h), fosfomycin (200 mg/kg every 12 h), and colistin (5 mg/kg per day), based on observed MIC distributions for these antibiotics. MIC=minimum inhibitory concentration. EUCAST=European Committee on Antimicrobial Susceptibility Testing.

Probability of target attainment values of 80% or more were predicted in 26 neonates (33·7% [SD 0·59]) of 78 with ampicillin–gentamicin; in 15 (68·0% [3·84]) of 27 with amoxicillin clavulanate–amikacin; 93 (92·7% [0·24]) of 109 with ceftazidime–amikacin; and 70 (85·3% [0·47] of 76 with piperacillin–tazobactam–amikacin. A reduction in probability of target attainment was most commonly predicted when there was resistance against both antibiotics of a combination treatment (figure 4). Probability of target attainment values aligned with observed survival rates (figure 4) for most treatments, except ampicillin–gentamicin, for which observed survival was higher than the probability of target attainment (33·7% *vs* 89·7%). Major differences in dosing schedules and resulting probability of target attainment values existed across study sites (appendix p 42). Sensitivity analyses showed that assumptions made regarding partly missing patient information did not significantly alter simulated probability of target attainment values (appendix p 43). In 290 neonates, mean probability of target attainment values of 80% or more adjusted to patient-specific MIC values were high for meropenem (290 [100%, SD 0]), fosfomycin (256 [89%, 0·16]) and colistin (290 [100%, 0]; figure 4). Reliable neonatal pharmacokinetic studies were available for meropenem; however, there were only a few fosfomycin and colistin pharmacokinetic studies, which moreover had a small sample size.^27^, ^28^, ^29^

**Figure 5:**
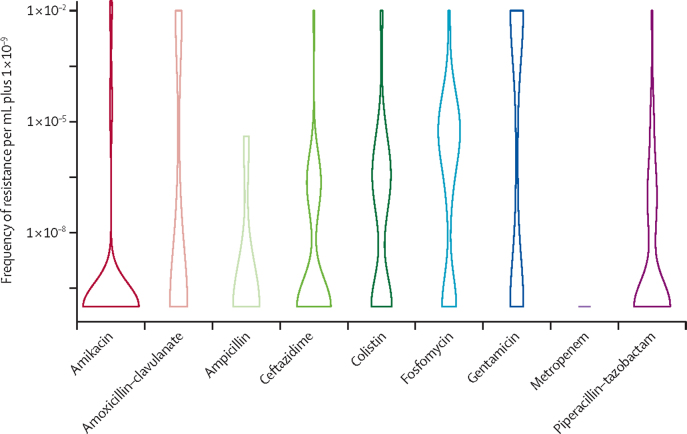
Frequency of resistance for Gram-negative isolates Numbers included in the analysis and mean growth per mL were: amikacin n=117, 3·75 × 10^−4^; amoxicillin–clavulanate n=24, 2·50 × 10^−3^; ampicillin n=6, 6·67 × 10^−7^; ceftazidime n=52, 1·92 × 10^−4^; colistin n=96, 4·17 × 10^−4^; fosfomycin n=114, 3·59 × 10^−4^; gentamicin n=117, 4·45 × 10^−3^; meropenem n=117, 0·00; and piperacillin–tazobactam n=102, 1·97 × 10^−4^. The numbers of isolates differed across antibiotics because of susceptibility patterns, with only sensitive bacteria suitable for frequency of resistance determination. Data are presented per mL and the frequency of resistance calculated from growth at a lower dilution on control plates free from antibiotics. Results have been log-transformed with a standard of 1 × 10^−10^ added to enable incorporation of zero values. This standard was chosen as the lowest rate of frequency of resistance found was 1 × 10^−9^.

Resistance occurred most frequently with fosfomycin (in 78 isolates [68·4%] of 114), followed by colistin (55 [57·3%] of 96), gentamicin (62 [53·0%] of 117), piperacillin–tazobactam (35 [34·3%] of 102), amoxicillin clavulanate (eight [33·3%] of 24), ceftazidime (17 [32·7%] of 52), ampicillin (one [16·7%] of six), amikacin (nine [7·7%] of 117) and meropenem (0 of 117; figure 5). Growth per mL was highest for gentamicin (4·44 × 10^−3^), with significantly higher growth per mL than all other antibiotics tested (ANOVA p<0·05; figure 5). Isolates for *E coli* had lower frequency of resistance than *K pneumoniae* isolates for most antibiotics tested (appendix p 44). Gram-negative species had varied frequencies of resistance to each antibiotic (appendix pp 45–47). Gram-positive isolates did not develop resistance against amikacin, although they did develop resistance against fosfomycin (one [5·3%] of 19), flucloxacillin (seven [36·8%] of 19) and gentamicin (four [21·1%] of 19; appendix p 47).

No correlations were found between pathogenicity indexing and virulence factors scores (*R*^2^=0·00–0·14); therefore both were analysed separately against outcomes. Lower virulence factor scores for *E coli* were associated with reported mortality (*U*=12·50, p=0·042) with no association between pathogenicity index and outcome (*U*=33·00, p=0·84). No associations between virulence factors or pathogenicity index and outcome were found for *K pneumoniae* (*U*=188·0, p=0·66; *U*=178·50, p=0·52, respectively) *or S aureus* (*U*=128·0, p=0·63; *U*=113·0, p=0·95, respectively; appendix p 48).

All sites except Nigeria offer ampicillin–gentamicin free of charge (table). Only sites in India and South Africa cover the costs of second-line or third-line antibiotics. For all other sites, access permitting, the patients bore the cost, which was as high as US$14·00 per day for meropenem; $8·00 per day for colistin; and $30·00 per day for tigecycline, often exceeding daily incomes. Costs varied greatly between sites, even within countries. Sites in six of the seven countries, except for South Africa, confirmed that cost of the antibiotic would influence treatment of neonatal sepsis (table).

**Table:** Health-care matrix consisting of average salaries, costs of antibiotics, and coverage by governments across different BARNARDS study countries

		**Bangladesh**	**Ethiopia**	**India**	**Nigeria**	**Pakistan**	**Rwanda**	**South Africa**
Average monthly salary		$228 (BK); $663 (BC)	$142	$98	$81 (NK); $204 (NW); $274 (NN)	$316 (PP); $100 (PC)	$250 (RK); $102 (RU)	$274
Who pays for the antibiotics?		Colistin, piperacillin–tazobactam, and tigecycline are paid for by the patient and not supplied by the hospital	Only ampicillin and gentamicin are available through the public system	Cost of antibiotics for neonates are borne by the West Bengal Government*	Cost of all drugs are mainly borne by patients; some financial support for government employees is given	Colistin, meropenem, and tigecycline are paid for by the patient	80% of the population have insurance, which covers payment for antibiotics; little data available on those who cannot afford insurance	Government covers the cost of all antibiotics
Is there private insurance and what role does it play?		Yes, but only for individuals with high incomes	Yes, but available to only a few people; poor people are not covered	Yes; widely used in India but not needed for the respondent's hospital (Institute of Post Graduate Medical Education and Research)	Yes, but available to only a few people; most cannot afford this and are not covered	Yes; private insurance covers <1% of the population. Poor patients are covered by the government	Yes; of those with insurance, companies cover 85% of treatment costs	Yes; approximately 20% of the country uses private health care
Local cost of antimicrobials per day (percentage of average daily wage based on 30 days per month)†								
	Ampicillin	$0·35 (2–5%)	$0·50 (11%)	$0·15 (5%)	$1·50 (17–56%)	$0·50 (1–5%)	$0·50 (6–15%)	$0·60 (7%)
	Gentamicin	$0·20 (1–3%)	$0·30 (6%)	$0·20 (6%)	$1·00 (11–38%)	$0·60 (1–6%)	$0·50 (6–15%)	$0·40 (4%)
	Ceftazidime	$3·00 (1-40%)	$3·50 (74%)	$2·50 (76%)	$3·50 (38–130%)	$2·30 (6–22%)	$2·00 (24–59%)	$1·80 (20%)
	Amikacin	$0·50 (2–7%)	Not available	$1·00 (30%)	$3·00 (33–111%)	$0·50 (1–5%)	$2·00 (24–59%)	$0·40 (4%)
	Amoxicillin–clavulanate	Not available	Not available	$8·00 (242%)	$10·00 (110–370%)	Not available	Not available	Not available
	Piperacillin–tazobactam	$24·60 (111–309%)	Not available	$2·60 (79%)	$20·00 (219–741%)	$9·00 (22–86%)	Not available	$7·20 (79%)
	Meropenem	$10·00 (45–132%)	$11·00 (234%)	$6·40 (194%)	$12·50 (137–463%)	$6·50 (62–197%)	$14·00 (169–412%)	$3·50 (38%)
	Colistin	$8·00 (36–105%)	Not available	$9·00 (272%)	Not available	$8·00 (19–76%)	Not available	$6·00 (66%)
	Tigecycline	Not available	Not available	$45·00 (1363%)	Not available	$30·00 (73–286%)	Not available	$27·00 (297%)
Does antibiotic cost influence accessibility?		Yes	Yes	Yes	Yes	Yes	Yes	No

Site acronyms are provided where more than one clinical site in a country has participated in the BARNARDS study. Clinical sites included Kumudini Women's Medical College (BK) and Chittagong Ma O Shishu Hospital (BC), Bangladesh; St Paul's Hospital Millennium Medical College, Ethiopia; Institute of Post Graduate Medical Education and Research, Kolkata, India; Murtala Mohammad Specialist hospital, Kano (NK), Wuse District Hospital, Abuja (NW), and National Hospital Abuja (NN), Nigeria; Pakistan Institute of Medical Sciences (PP), and Community health centre, Bhara Kahu (PC), Pakistan; University Central Hospital of Kigali (RK) and Kabgayai Hospital (RU), Rwanda; and Tygerberg Academic Hospital, Cape Town, South Africa. Costs are given in US dollars for ease of comparison, with exchange rates calculated through www.XE.com, March 2020. BARNARDS=Burden of Antibiotic Resistance in Neonates from Developing Societies.

* India is a federal union split into 28 states and 8 union territories. Reliability for the information displayed in this table for India is limited to the state of West Bengal, where the clinical site in India is located.

† Percentage cost of average daily wages are provided as averages per site within each country for two sites within a country. Percentages of average daily wage are presented as a single percentage for countries with a single site, or as a range for countries with two or more clinical sites, because of varied average wages and demographics between sites.

## Discussion

High levels of antimicrobial resistance were found across both Africa and Asia, slightly higher in south Asia for Gram-negative isolates in keeping with previously published data,^30^, ^31^ with higher resistance in Gram-positive isolates from Africa. Extremely high resistance was found against ampicillin of more than 97% in Gram-negative isolates, and *Staphylococcus* spp, which are regarded as intrinsically resistant to ampicillin;^16^ therefore, it can be argued that ampicillin is now redundant for treating neonatal sepsis in LMIC settings. Gentamicin with 70·2% resistance in Gram-negative bacteria also did not have significant activity. Coverage for ampicillin–gentamicin was 28·5% in Gram-negative isolates, further indicating the need for re-evaluation of the WHO recommendation for managing neonatal sepsis.

Empirical treatment for neonatal sepsis in LMICs is based on data from high-income countries (HICs) because of the sparsity of data from LMICs.^7^, ^12^ Ampicillin is effective against pathogens common in HICs, such as group B streptococci and *Listeria*,^7^, ^30^, ^31^ although these species were not reported in this study, with reduced prevalence of these isolates in LMICs supported by previous studies.^7^, ^32^, ^33^ Empirical therapies used by BARNARDS sites showed deviation from use of ampicillin–gentamicin, commonly substituting it with amoxicillin clavulanate–amikacin, piperacillin–tazobactam–amikacin, or ceftazidime–amikacin, as supported by previous studies^8^ because of reduced clinical response to ampicillin–gentamicin.^7^

Amikacin, regularly used by sites, showed greater activity (74·0% susceptibility in Gram-negative isolates) than ampicillin and when paired with ceftazidime (which is on the WHO Watch list)^10^ provided 77·1% coverage against Gram-negative isolates. All BARNARDS sites except in Ethiopia had access to amikacin. Although more expensive (ranging from $0·40–3·00 per day compared with $0·15–1·50 per day for gentamicin), this was a small increase compared with alternative antibiotics. In all BARNARDS sites, ceftazidime was more expensive ($1·80–3·50 per day) than ampicillin ($0·15–0·60 per day), which might affect the choice of empirical use. Although reported mortality was low among neonates treated with ampicillin–gentamicin, it was significantly lower with ceftazidime–amikacin. This was negated when country-specific analysis was applied. Ampicillin–gentamicin was prescribed across multiple sites, although other combinations were predominant at certain sites—eg, ceftazidime–amikacin was mainly prescribed in Bangladesh, amoxicillin clavulanate–amikacin mainly in Nigeria, and piperacillin–tazobactam predominantly in Pakistan. Therefore, antibiotic effects could not be distinguished from country effects, which had different facilities and training and demands on staff, in addition to prevalence of resistance, bacterial strains, and patient characteristics. Some sites might be exposed to counterfeit antibiotics,^34^ which could also alter outcomes.

Probability of target attainment levels of 80% or more were low for ampicillin–gentamicin because of high levels of resistance, compared with the other treatment combinations of ceftazidime–amikacin, piperacillin–tazobactam–amikacin, and amoxicillin clavulanate–amikacin. Simulated probability of target attainment variation was small for all combinations, except for amoxicillin clavulanate–amikacin, for which significant inter-individual variation was observed. This study did not determine patient-specific antibiotic concentrations. However, variation in dosing schedules across sites and countries is an important factor to consider when comparing differences in treatment outcomes. The high reported survival rate relative to ampicillin–gentamicin probability of target attainment values could have arisen from under-reporting of mortality, as neonates not reported as deceased who were treated with ampicillin–gentamicin were followed up for fewer than 10 days (59·1% of those treated with ampicillin–gentamicin *vs* 23·7% of those treated with ceftazidime–amikacin). Follow-up is particularly difficult in LMIC settings, where mothers might live far away from clinical sites, or not have a contact number.

Amoxicillin clavulanate was not available in five of the seven countries in the study, and piperacillin–tazobactam was expensive (ranging from $2·60–24·60 per day). A high level of mortality was reported for neonates treated with piperacillin–tazobactam–amikacin (27·8%), despite high coverage for Gram-negative isolates. This might be due to confounding factors, as piperacillin–tazobactam–amikacin is often used for nosocomial infections for which neonates were already admitted to hospital; however, the severity of neonatal sepsis was not recorded. This might again represent country effect, because most of the prescriptions for piperacillin–tazobactam–amikacin were from sites in Pakistan. Outcomes might be negatively affected where patients bear the cost of antibiotic therapies and might not have been able to afford full courses of antibiotics.

Resistance to meropenem and fosfomycin (both on the WHO Watch list) and acquired resistance to colistin (on the WHO Reserve list) were low in addition to high probability of target attainment values simulated.^35^, ^36^, ^37^ Frequency of resistance assessments, proposed by Sommer and colleagues^19^ to gauge robustness of antibiotic regimes, also suggested in our study that meropenem is a strong candidate as a single therapy.^36^ However, accessibility and costs currently preclude meropenem, fosfomycin, and colistin as treatment regimens for neonatal sepsis in LMICs. High frequency of resistance was seen against fosfomycin, supported by previous studies.^37^ However, growth per mL was similar to other antibiotics, potentially showing the presence of mutations that were unstable because of their fitness costs. However, da Campos and colleagues^38^ found that resistance had no or only minor effects on bacterial fitness in *E coli* strains; further work needs to be undertaken investigating the stability of fosfomycin resistance across bacterial species. Frequency of resistance for gentamicin was much higher than for amikacin, with significantly higher growth per mL, implying amikacin to be more robust, which might help to prevent selection for resistance over time with increased usage.

Associations were not found between pathogenicity or virulence factors and outcome, except for low virulence factor scores for *E coli* associated with higher reported mortality in neonates. It is possible that although an *E coli* bacterium might have numerous virulence factor genes, these might not be expressed or regulated. Discrepancies between outcome and microbiological data might be partly due to the numbers of neonates lost to follow-up. Antimicrobial resistance can affect bacterial virulence, but it is mechanism-dependent, which could possibly account for the absence of correlations.^39^

This substudy of a large international prospective study provides vital insights into antimicrobial usage and efficacy in LMICs, although it had several limitations. Outcomes were not always obtainable as neonates were sometimes lost to follow-up. This is an issue with observational studies, particularly within LMICs, where mothers might live far from clinical sites or have no telephone for contacting. Neonatal bodyweight was not reported and was estimated from gestational and postnatal age for pharmacokinetic and pharmacodynamic analysis.^17^ There are no EUCAST breakpoints for some antibiotic–bacteria combinations; we extrapolated breakpoints from similarly related species. Frequency of resistance data were generated for single antibiotics only because it is problematic to choose concentrations for antibiotic combinations. Patient-specific dosing regimens were not available. Isolates were selected on the basis of antibiotic therapy given and available data, potentially biasing the inclusion of neonates from sites with information on antibiotic usage. Differences in antibiotics used per site led to indistinguishable country versus treatment effects for outcome. Clinical data did not state the severity of sepsis before treatment.

This study uniquely combines epidemiology, microbiology, pharmacokinetic and pharmacodynamic data, and health-cost matrices to understand immediate and future issues facing the effective treatment of neonatal sepsis in LMICs. Our combined MIC, frequency of resistance, and probability of target attainment data suggest that ampicillin is redundant in these settings and that the WHO-recommended combination of ampicillin–gentamicin for treatment of neonatal sepsis requires further scrutiny for LMICs. The results in this study suggest that ceftazidime–amikacin might be an effective potential alternative to ampicillin–gentamicin in LMICs. Further studies are urgently required to evaluate alternatives on neonatal outcomes.

## Data sharing

Data will be made available upon request, following assessment from the research team and strictly adhering to patient confidentiality and consent. Whole genome sequencing data have been submitted to the European Nucleotide Archive (ENA) and can be accessed via the ENA browser (https://www.ebi.ac.uk/ena/browser/home), as referenced in the main text and single accession numbers referenced in the appendix (pp 8–10). Excel files for pathogenicity indexing, virulence factor scores, MIC profiles, and deidentified data for demographic characterics of neonates included within this study can also be made available upon request. All relevant study protocols including ethical and consent forms are on the BARNARDS group website (www.barnards-group.com). Datasets specific to this study will be made available upon request following publication. Requests for access to additional data should be made directly to TRW via email: timothy.walsh@zoo.ox.ac.uk.

## Declaration of interests

We declare no competing interests.
