# Supplementary material for: Effects of antibiotic resistance, drug target attainment, bacterial pathogenicity and virulence, and antibiotic access and affordability on outcomes in neonatal sepsis: an international microbiology and drug evaluation prospective substudy (BARNARDS)
Source: Lancet Infect Dis. 2021 Dec;21(12):1677–88. doi: 10.1016/S1473-3099(21)00050-5 (PMC8612937; doi:10.1016/S1473-3099(21)00050-5)
Supplement: Supplementary appendix [file mmc1.pdf]

THE LANCET
Infectious Diseases

Supplementary appendix

This appendix formed part of the original submission and has been peer reviewed. We post it as supplied by the authors.

Supplement to: Thomson KM, Dyer C, LiuF, et al. Effects of antibiotic resistance, drug target attainment, bacterial pathogenicity and virulence, and antibiotic access and affordability on outcomes in neonatal sepsis: an international microbiology and drug evaluation prospective substudy (BARNARDS). *Lancet Infect Dis* 2021; published online August 9. https://doi.org/10.1016/S1473-3099(21)00050-5.

Data sharing statement

Data will be made available upon request, following assessment from the research team and strictly adhering to patient confidentiality and consent. Whole genome sequencing data have been submitted to the European Nucleotide Archive and can be accessed via ENA browser (https://www.ebi.ac.uk/ena/browser/home), as referenced in the main text and single accession numbers referenced on Appendix, pages 8-10. Excel files for pathogenicity indexing, virulence factor scores, MIC profiles and deidentified demographic data of neonates included within this study can also be made available upon request. All relevant study protocols including ethical and consent forms are on the BARNARDS group website (www.barnards-group.com). Links for specific protocols are referenced below. Datasets specific to this study will be made available upon request immediately following publication. Requests for access to additional data should be made directly to Professor Walsh, via email: timothy.walsh@zoo.ox.ac.uk

Links to a range of standard operating procedures (SOPs) and methodology for BARNARDS can be found on the BARNARDS website https://barnards-group.com/publications/

Specific URLS for each SOP can be found at the following links:

1. Appendix E: Ethics template: https://barnards-group.com/wp-content/uploads/2020/05/Appendix-E-Ethics-Template.pdf
2. Ethical approval per country can be found via https://barnards-group.com/publications/
3. Appendix C: Consent form template: https://barnards-group.com/wp-content/uploads/2020/05/Appendix-C-Consent-Form-Template.pdf
4. Appendix N: Neonatal sepsis clinical presentation worksheet: https://barnards-group.com/wp-content/uploads/2020/05/Appendix-N-Neonatal-Sepsis-Signs-of-Clinical-Presentation.pdf
5. Appendix P: Phlebotomy checklist: https://barnards-group.com/wp-content/uploads/2020/05/Appendix-P-Phelbotomy-checklist.pdf
6. Appendix M: Microbiology standard operating procedures at clinical sites: https://barnards-group.com/wp-content/uploads/2020/05/Appendix-M-Microbiology.pdf
7. Appendix F: Follow up SOP: https://barnards-group.com/wp-content/uploads/2020/05/Appendix-F-Follow-up-appendix.pdf

Supplementary Table 1. Breakpoints used to determine susceptibility profiles from Gram-negative bacteria analysed (EUCAST v9.0[1]). Enterobacteriaceae breakpoints were available for all antibiotics tested, except for Minocycline. PK-PD breakpoints were also not available; therefore SIR was not analysed for minocycline. Where available, breakpoints for *Pseudomonas* sp. as determined by EUCAST. However, where these were not available, Enterobacteriaceae breakpoints were used. Similarly, *Pseudomonas* sp. (green) or Enterobacteriaeceae (blue) breakpoints were used to determine *Acinetobater* sp. isolate resistance. Where breakpoints were not available for *Stenotrophomonas* sp., *Acinetobacter* sp. breakpoints were used were available (orange), followed by *Pseudomonas* sp. (green) or Enterobacteriaceae (blue). Enterobacteriaceae breakpoints were used for *Aeromonas* sp. isolates where no confirmed breakpoints were available.

**Antibiotic**	**Enterobacteriaceae**		**Pseudomonas***		**Acinetobacter**		**Stenotrophomonas**		**Aeromonas**		**PK-PD**	
	**≤S**	**>R**	**≤S**	**>R**	**≤S**	**>R**	**≤S**	**>R**	**≤S**	**>R**	**≤S**	**>R**
Ampicillin	8	8	8	8	8	8	8	8	8	8	2	8
Amoxicillin-clavulanate	8	8	8	8	8	8	8	8	8	8	2	8
Piperacillin-tazobactam	8	16	16	16	16	16	16	16	8	16	4	16
Ceftriaxone	1	2	1	2	1	2	1	2	1	2	1	2
Cefotaxime	1	2	1	2	1	2	1	2	1	2	1	2
Ceftazidime	1	4	8	8	8	8	8	8	1	4	4	8
Cefepime	1	4	8	8	8	8	8	8	1	4	4	8
Imipenem	2	4	4	4	2	4	2	4	2	4	2	4
Meropenem	2	8	2	8	2	8	2	8	2	8	2	8
Ertapenem	0.5	0.5	0.5	0.5	0.5	0.5	0.5	0.5	0.5	0.5	0.5	0.5
Aztreonam	1	4	16	16	16	16	16	16	1	4	4	8
Gentamicin	2	4	4	4	4	4	4	4	2	4	-	-
Amikacin	8	16	8	16	8	16	8	16	8	16	-	-
Tobramycin	2	4	4	4	4	4	4	4	2	4	-	-
Tigecycline	ECOFF values for respective species used*2										0.5	0.5
Minocycline	NA	NA	NA	NA	NA	NA	NA	NA	NA	NA	-	-
Fosfomycin	32	32	32	32	32	32	32	32	32	32	-	-
Levofloxacin	0.5	1	1	1	0.5	1	0.5	1	0.5	1	0.5	1
Ciprofloxacin	0.25	0.5	0.5	0.5	0.25	1	0.25	0.5	0.25	0.5	0.25	0.5
Colistin	2	2	2	2	2	2	2	2	2	2	-	-

***Burkholderia* sp. and *Ralstonia* sp. isolates were analysed with the same breakpoints as *Pseudomonas* sp., as these species are closely related and not in EUCAST v9.0.
*2ECOFF values can be found for Tigecycline through link provided in the reference list.[2]

Supplementary Table 2. Breakpoints used to determine susceptibility profiles from Gram-positive bacteria analysed (EUCAST v9.0[1]). No breakpoint is provided for ampicillin, as most are penicillinase producers and considered resistant[1]

**Antibiotic**	**Staphylococcus sp.**	
	**≤S**	**>R**
Ampicillin	-	-
Oxacillin	2	2
Flucloxacillin	2	2
Levofloxacin	1	1
Ciprofloxacin	1	1
Gentamicin	1	1
Amikacin	8	16
Tobramycin	1	1
Tigecycline	0.5	0.5
Minocycline	0.5	1
Rifampicin	0.06	0.5
Vancomycin	2	2
Azithromycin	1	2
Linezolide	4	4

MICs were undertaken in batches of 80 isolates, including control strain for either Gram-negative or Gram-positive isolates at Cardiff University, UK. Control strains were included in every batch. Control strains for Gram-negative batches included: *E. coli* ATCC 25922; *Pseudomonas aeruginosa* ATCC 27853; VIM-positive *Pseudomonas aeruginosa* A70; NDM-1-positive *E. coli* IR60; NDM-1-positive *K. pneumoniae* IR35. Control strains for Gram-positive batches included: *E. coli* ATCC 25922; *Pseudomonas aeruginosa* ATCC 27853; *Staphylococcus aureus* 29213*; Staphylococcus aureus* 25923; NDM-1-positive *E. coli* IR60.

Literature search for Heat Maps

We searched for the terms “Enterobacteriaceae antibiotic resistance” and “country” and sought studies possessing susceptibility to all four antibiotics shown in the heat maps. Original searches started with “neonatal sepsis” and then “sepsis” and were broadened to include other clinical syndromes. Moreover, where possible we chose studies possessing over 100 isolates. Where national reference reporting was available i.e. Canada, the data was taken from the most recent datasets. Not all countries were searched using the above terms and therefore, the heat maps are not meant to show a comprehensive review but a reflection on the global picture of ampicillin versus ceftazidime, and gentamicin versus amikacin.

Resistance data from the main BARNARDS project were also incorporated into these heatmaps for BARNARDS sites in Bangladesh, Indian, Pakistan, Ethiopia, Rwanda, South Africa and Nigeria.

Extended PK/PD methodology description

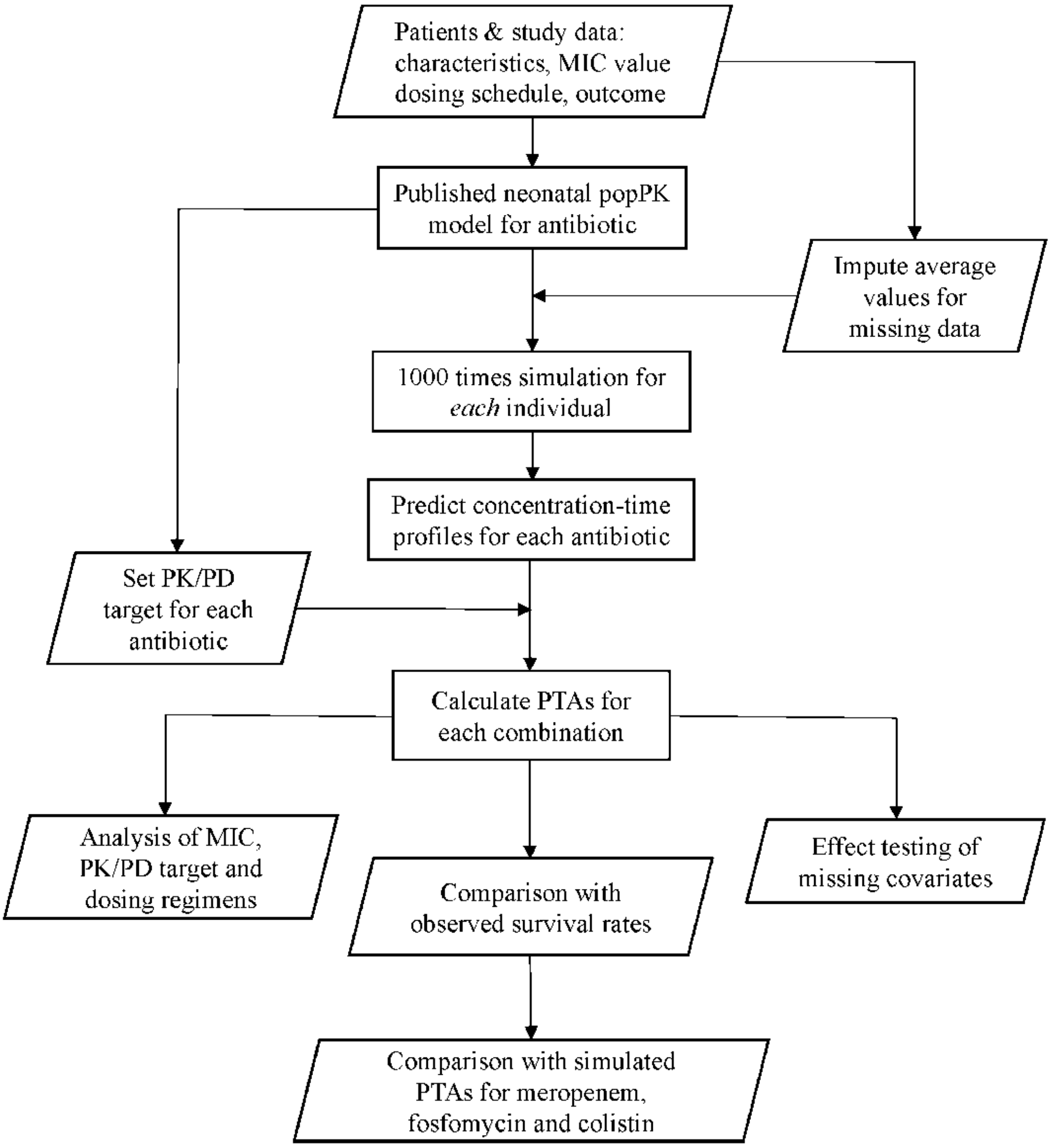

Supplementary Figure 1. An overview of the methodology is depicted in below flow chart.

Patient characteristics data

Two subsets of the data were used including either n=290 patients or n=476 patients. For both datasets we included only patients who received one of the four most commonly used antibiotic combinations, which included six antibiotics (ampicillin, amikacin, amoxicillin, ceftazidime, gentamicin and piperacillin). The dataset of n=290 patients only included patients who received a single combination treatment, whereas the larger dataset of n=476 also included patients who received another antibiotic (combination) regimen. Age, gender, patient-specific MIC, antibiotic dosing information, and study-site specific dosing schedules were collected.

Gestational and postnatal age assumptions

Missing gestational ages (GA) and postnatal ages (PNA) were imputed with the average age for preterm and no-preterm infants by sites. GAs for no-preterm infants were assumed to be either 40 weeks (on time) or 42 weeks (late). Postmenstrual ages (PMA) were calculated by PNA adding GA.

Estimation of body weight

Birth weight was estimated by ages and genders from a global reference (Eq. 1).[3] Ratios of global and local estimates were calculated to predict the region-specific birth weight. Postnatal weight was estimated based on a

growth standard for preterm from Lancet (Eq. 2)[4] and a WHO standard for no-preterm (Eq. 3-4) with a shifted ratio between the estimated birthweight by Lancet and WHO standard by sites.[5] Average birth and postnatal weight were imputed for patients whose gender information were missing.

Estimation for serum creatinine and serum albumin
Serum creatinine (SCR) was also computed using ages which are based on a reference for no-preterm infants (Eq. 5).[6] Detected creatinine values were adopted for preterm newborns younger than 1 month.[7] For preterm infants older than 1 month, the reference for no-preterm infants was used after adjusting by a ratio of no-preterm and preterm values at day 28. Plasma albumin (ALB) was also estimated by an age-related equation for children (Eq. 6).[8]

$$Birthweight(g) = exp(0.578 + 0.332 \times GA - 0.00354 \times GA^2) \times Ratio(Eq.1)$$
$$Weight(kg) = exp(2.591 - 0.012 \times PMA^{0.5} - 2201.705 \times PMA^{-2} + 0.091 \times Sex)$$
$$Sex = 1 formale, 0 forfemale (Eq.2)$$
$$Formale: weight(kg) = BCPE(x = Age^{0.35}, df(\mu) = 11, df(\sigma) = 7, df(v) = 4, \tau = 2) * Ratio(Eq.3)$$
$$Forfemale: weight(kg) = BCPE(x = Age^{0.35}, df(\mu) = 11, df(\sigma) = 7, df(v) = 3, \tau = 2) * Ratio(Eq.4)$$
$$SCR(^{\mu mol}/_{L}) = 10^{(1.75 - 0.07 \times log_2(Age)} \times Ratio(Eq.5)$$
$$ALB(^{g}/_{L}) = 1.1287 \times ln(Age) + 33.746(Eq.6)$$

MIC data handling
For MIC values recorded at the boundaries of their possible values, we assumed the next-lowest or next-highest dilution. In case of missing MIC data we performed random sampling based on the empirical MIC density.

PK/PD simulations to calculate target attainment for the 4 most commonly used antibiotics
For the six antibiotics studied (ampicillin, amikacin, amoxicillin, ceftazidime, gentamicin and piperacillin) we selected published neonatal population pharmacokinetics (PK) models.[9-14] Using the population PK models, for each individual we performed 1000 simulations based on their patient characteristics including MIC and specific dosing schedule used, using the R package RxODE. As PK/PD target we used 50% fT>MIC and Cmax/MIC>8 were used as PK/PD targets for beta-lactam (70% for ceftazidime)[15] and aminoglycosides[16], respectively. Subsequently, the percentage of PK/PD target attainment (PTA) for each combination by site was computed, determining if at least one of the targets in a combination was reached. PTAs ≥80% was applied as an index of efficacy and 100 times of above simulations were implement to get the distribution of PTAs ≥80%. The final PTAs ≥80%, shown in mean ± SD, for these four combinations were compared with the actual survival rate for each combination in this study.

For calculating the PTA per country to assess differences in dosing schedules we used the n=476 patients dataset because here we aimed to identify the impact of differences in dosing schedules used in different countries. For all other PTA calculations we used the single treatment n=290 dataset.

Sensitivity analyses
We performed sensitivity analyses to test the effect of assumptions made regarding missing sex for PTA calculations of all 4 combinations. We also tested the effect of ignoring the effect of co-administration of ibuprofen or dopamine was a covariate for population PK models for gentamicin or amoxicillin. We used a t-test to determine if PTA values were statistically different.

PK/PD simulations for meropenem, fosfomycin and colistin monotherapies
We performed PK/PD simulations for meropenem, fosfomycin and colistin, following the same steps as described for the other antibiotics. For meropenem, a published population PK model in neonates[17] and a recommended dosage of 10mg/kg every 8h were chosen for simulation[18]. 50% fT>MIC were used as the PK/PD target, assuming an approximate protein binding of 2%[15,17].

For fosfomycin and colistin, pharmacokinetics parameter (e.g. clearance and distribution volume) were based on previous studies[19-21.] Estimates for inter-individual variability were also included.[21,22] The fraction of colistin methate sodium (CMS) converted to colistin was based on a preclinical study.[23] The maximum recommended dose regimens were selected for simulation.[24,25] A 40%fT>MIC with 3% protein binding rate[26,27] and the

average steady state concentration (Css,avg) >2.0mg/L[28] were chosen as PK/PD targets for fosfomycin and colistin, respectively.

Dosing regimens used for simulations		
**Meropenem**	**Fosfomycin**	**Colistin**
10 mg/kg every 8 h	200 mg/kg every 12h	5 mg/kg per day

Antibiotics and isolate selection for FoR

Antibiotics for inclusion in FoRs were selected based on use as a current treatment and those antibiotics shown to have lower rates of resistance from the overall BARNARDS MICS dataset[1] and were thought to be potentially suitable alternative options for treatment of neonatal sepsis. Antibiotics chosen for FoR analysis included ampicillin, amikacin, amoxicillin-clavulanate, ceftazidime, gentamicin, piperacillin-tazobactam, fosfomycin, meropenem, and colistin.

Isolates were selected based on availability of antibiotic data and MIC profiles. Species commonly found as sepsis pathogens were chosen to select from, which included *Acinetobacter* spp.; *Burkholderia* spp.; *Enterobacter* spp.; *Escherichia coli; Klebsiella* spp.; *Pseudomonas* spp.; *Serratia marcescens;* and *Ralstonia mannitolilytica.* Isolates were then filtered for susceptibility towards meropenem, amikacin, fosfomycin, gentamicin and colistin.

For each species selected for inclusion, phylogenetic groups were divided via clusters/ clades and key sequence types were identified. Representative isolates were then chosen from the filtered list of sensitive isolates.

This process was repeated separately for isolates susceptible to ampicillin, as a lower number of sensitive isolates were available.

Isolate selection PI and VF

Isolate selection for pathogenicity was based on the isolates which were analysed for FoRs with additional *S. aureus*, *E. coli* and *K. pneumoniae* isolates. The additional isolates were selected based on including a range of sequence types of each species, as well as considering various clinical outcome to give a representation of the pathogens found in BARNARDS. Isolates were additionally chosen based on a range of MICs for AMP-GEN.

Virulence factor scores were given to each *E. coli, K. pneumoniae* and *S. aureus* isolates with PI analysed, which denoted how many virulence factor genes each isolate had.

A colony of *E. coli, K. pneumoniae* or *S. aureus* was transferred into 1.8ml of LB broth and incubated at 37°C, 180 rpm for 18hours. Genomic DNA was extracted on the QIAcube (Qiagen, Germany) using the QIAmp DNA mini kit (Qiagen, Germany), with an additional RNAse step. DNA was quantified using the Qubit fluorometer 3.0 and Genomic libraries were prepared using Nextera XT V2 (Illumina, USA), with bead-based normalisation as per manufacturer guidelines. Paired end WGS, was performed on an Illumina MiSeq using the V3 chemistry to generate fragment lengths up to 300bp (600 cycles). Bioinformatics analysis was performed using a high-performance computing cluster at Cardiff University (ARCCA). Quality control of sequence reads was performed using fastqc (v0.11.2)[29] and Trimgalore (v0.4.3)[30]. Paired-end reads were overlapped using Flash (v1.2.11)[31] and assembled into contigs using SPAdes (v3.9.0)[32], with the resulting contigs mapped back to the raw sequence reads using BWA (v.0.7.15)[33] and samtools (v1.3.1)[34]. Pilon (v1.22)[35] was then used to assess any misassembles/errors in base calling in the mapped BAM file. Genome assembly metrics were generated using quast (v.2.1)[36]. Species identification was performed using PathogenWatch[37]. Genomes were screened for virulence factors using Abricate (v0.9.7) and vfdb. Outputs were filtered in Microsoft Excel and genes ⩾98% identity were included to create a total virulence factor score per isolate. Virulence factor scores were given to each *E. coli, K. pneumoniae* and *S. aureus* isolates with PI analysed, which denoted how many putative virulence associated genes each isolate had.

Bioinformatics script parameters used

- trim_galore --paired --phred33 -q 25 --nextera -e 0.2[29]
- spades.py -k 21,33,55,77,99,127 --careful -1 [isolate_code]_R1 -2 [isolate_code]_R2 -o [isolate_code]
- bwa index [isolate_code].fasta | bwa mem [isolate_code].fasta [isolate_code]_R1 [isolate_code]_R2 > [isolate_code].sam | samtools sort [isolate_code].sam -o [isolate_code]_mapped.bam | samtools index [isolate_code]_mapped.bam
- pilon-1.22.jar --changes --mindepth 0.5 –genome –frags [isolate_code]_mapped.bam --output [isolate_code].fasta
- abricate –db vfdb > [isolate_code].txt

Supplementary Table 3. Accession numbers for isolates included in VF analysis.

**Species**	**Isolate ID**	**ENA accession**
Escherichia coli	BC-BB312-I	ERS5229805 (SAMEA7472110)
Escherichia coli	BC-BB322-I	ERS5229806 (SAMEA7472111)
Escherichia coli	ESS-BB0379a-I1	ERS5229993 (SAMEA7472298)
Escherichia coli	ESS-BB0140-I1	ERS5229975 (SAMEA7472280)
Escherichia coli	ESS-BB0187-I1	ERS5229979 (SAMEA7472284)
Escherichia coli	NK-BB1367-I	ERS5230076 (SAMEA7472381)
Escherichia coli	NN-BB187-I	ERS5230180 (SAMEA7472485)
Escherichia coli	NN-BB499-I	ERS5230199 (SAMEA7472504)
Escherichia coli	PP-BB2700-I	ERS5230320 (SAMEA7472625)
Escherichia coli	PP-BB2812-I	ERS5230322 (SAMEA7472627)
Escherichia coli	PP-BB5340-I	ERS5230408 (SAMEA7472714)
Escherichia coli	RK-BB62-I	ERS5230567 (SAMEA7472873)
Escherichia coli	RK-BB103-I	ERS5230533 (SAMEA7472839)
Escherichia coli	RK-BB111-I	ERS5230537 (SAMEA7472843)
Escherichia coli	RK-BB1384-I	ERS5230541 (SAMEA7472847)
Escherichia coli	RK-BB1495-I	ERS5230544 (SAMEA7472850)
Escherichia coli	RK-BB2246-I	ERS5230555 (SAMEA7472861)
Escherichia coli	RK-BB91-I	ERS5230584 (SAMEA7472890)
Escherichia coli	RK-BB973-I	ERS5230587 (SAMEA7472893)
Escherichia coli	RU-BB339-I	ERS5230598 (SAMEA7472904)
Escherichia coli	ZAT-BB1448-I1	ERS5230618 (SAMEA7472924)
Escherichia coli	ZAT-BB279-I3	ERS5230636 (SAMEA7472942)
Klebsiella pneumoniae	BC-BB1210-I	ERS5229750 (SAMEA7472055)
Klebsiella pneumoniae	BC-BB1228-I	ERS5229751 (SAMEA7472056)
Klebsiella pneumoniae	BC-BB1283-I	ERS5229755 (SAMEA7472060)
Klebsiella pneumoniae	BC-BB296-I	ERS5229802 (SAMEA7472107)
Klebsiella pneumoniae	BC-BB980-I	ERS5229897 (SAMEA7472202)
Klebsiella pneumoniae	ESI-BB0616-I2	ERS5229913 (SAMEA7472218)
Klebsiella pneumoniae	ESI-BB1044b-I1	ERS5229920 (SAMEA7472225)
Klebsiella pneumoniae	ESI-BB1341a-I1	ERS5229924 (SAMEA7472229)
Klebsiella pneumoniae	ESI-BB1344b-I1	ERS5229926 (SAMEA7472231)
Klebsiella pneumoniae	ESI-BB1384a-I1	ERS5229927 (SAMEA7472232)
Klebsiella pneumoniae	ESO-BB1839b-I1	ERS5229954 (SAMEA7472259)
Klebsiella pneumoniae	ESO-BB2005-I1	ERS5229958 (SAMEA7472263)
Klebsiella pneumoniae	ESS-BB0547a-I1	ERS5230029 (SAMEA7472334)
Klebsiella pneumoniae	ESS-BB0139-I1	ERS5229974 (SAMEA7472279)
Klebsiella pneumoniae	ESI-BB1691-I1	ERS5229940 (SAMEA7472245)
Klebsiella pneumoniae	ESO-BB1839a-I1	ERS5229953 (SAMEA7472258)
Klebsiella pneumoniae	ESS-BB0259-I1	ERS5229982 (SAMEA7472287)
Klebsiella pneumoniae	ESS-BB0304a-I1	ERS5229987 (SAMEA7472292)
Klebsiella pneumoniae	ESS-BB0383-I1	ERS5229995 (SAMEA7472300)
Klebsiella pneumoniae	ESS-BB0405-I1	ERS5229997 (SAMEA7472302)
Klebsiella pneumoniae	ESS-BB0460b-I1	ERS5230015 (SAMEA7472320)
Klebsiella pneumoniae	ESS-BB0463-I2	ERS5230016 (SAMEA7472321)

Klebsiella pneumoniae	ESS-BB0482a-I1	ERS5230018 (SAMEA7472323)
Klebsiella pneumoniae	ESS-BB0490-I1	ERS5230020 (SAMEA7472325)
Klebsiella pneumoniae	ESS-BB0490-I2	ERS5230021 (SAMEA7472326)
Klebsiella pneumoniae	ESS-BB0515a-I1	ERS5230024 (SAMEA7472329)
Klebsiella pneumoniae	ESS-BB0531a-I1	ERS5230027 (SAMEA7472332)
Klebsiella pneumoniae	NK-BB1145-I	ERS5230067 (SAMEA7472372)
Klebsiella pneumoniae	NK-BB1495-I	ERS5230088 (SAMEA7472393)
Klebsiella pneumoniae	NN-BB1542r1-I1	ERS5230132 (SAMEA7472437)
Klebsiella pneumoniae	NN-BB1647-I	ERS5230157 (SAMEA7472462)
Klebsiella pneumoniae	NN-BB169-I	ERS5230165 (SAMEA7472470)
Klebsiella pneumoniae	NN-BB170-I	ERS5230168 (SAMEA7472473)
Klebsiella pneumoniae	NN-BB1721-I	ERS5230172 (SAMEA7472477)
Klebsiella pneumoniae	NN-BB455-I	ERS5230193 (SAMEA7472498)
Klebsiella pneumoniae	NN-BB492r1-I	ERS5230198 (SAMEA7472503)
Klebsiella pneumoniae	NW-BB182ar1-I	ERS5230210 (SAMEA7472515)
Klebsiella pneumoniae	PC-BB31-I	ERS5230217 (SAMEA7472522)
Klebsiella pneumoniae	PC-BB456-I	ERS5230223 (SAMEA7472528)
Klebsiella pneumoniae	PP-BB1935-I	ERS5230279 (SAMEA7472584)
Klebsiella pneumoniae	PP-BB2093-I	ERS5230293 (SAMEA7472598)
Klebsiella pneumoniae	PP-BB2859-I	ERS5230327 (SAMEA7472632)
Klebsiella pneumoniae	PP-BB6586-I	ERS5230453 (SAMEA7472759)
Klebsiella pneumoniae	RK-BB721b-I	ERS5230573 (SAMEA7472879)
Klebsiella pneumoniae	RK-BB1216-I	ERS5230539 (SAMEA7472845)
Klebsiella pneumoniae	RK-BB1813-I	ERS5230548 (SAMEA7472854)
Klebsiella pneumoniae	RK-BB866-I	ERS5230582 (SAMEA7472888)
Klebsiella pneumoniae	RU-BB193-I	ERS5230592 (SAMEA7472898)
Klebsiella pneumoniae	RU-BB284-I	ERS5230596 (SAMEA7472902)
Klebsiella pneumoniae	RU-BB487-I	ERS5230602 (SAMEA7472908)
Klebsiella pneumoniae	ZAT-BB14-I1	ERS5230617 (SAMEA7472923)
Klebsiella pneumoniae	ZAT-BB1262-I4	ERS5230615 (SAMEA7472921)
Klebsiella pneumoniae	ZAT-BB175-I2	ERS5230622 (SAMEA7472928)
Klebsiella pneumoniae	ZAT-BB1830-I1	ERS5230624 (SAMEA7472930)
Klebsiella pneumoniae	ZAT-BB514b-I1	ERS5230641 (SAMEA7472947)

Supplementary Table 3 cont. Accession numbers for isolates included in VF analysis.

**Species**	**Isolate ID**	**ENA accession**
Staphylococcus aureus	BC-BB1562-I	ERS5229022 (SAMEA7471326)
Staphylococcus aureus	BC-BB991-I	ERS5229026 (SAMEA7471330)
Staphylococcus aureus	ESS-BB0162-I1	ERS5229029 (SAMEA7471333)
Staphylococcus aureus	NK-BB1278-I	ERS5229033 (SAMEA7471337)
Staphylococcus aureus	NK-BB2412-I	ERS5229042 (SAMEA7471346)
Staphylococcus aureus	NN-BB129-I	ERS5229047 (SAMEA7471351)
Staphylococcus aureus	NN-BB1591-I	ERS5229049 (SAMEA7471353)
Staphylococcus aureus	NN-BB1604r1-I	ERS5229050 (SAMEA7471354)
Staphylococcus aureus	NN-BB1727a-I	ERS5229052 (SAMEA7471356)
Staphylococcus aureus	NN-BB1782-I	ERS5229054 (SAMEA7471358)
Staphylococcus aureus	NN-BB651-I	ERS5229055 (SAMEA7471359)

Staphylococcus aureus	PC-BB354b-I1	ERS5229056 (SAMEA7471360)
Staphylococcus aureus	PC-BB356-I	ERS5229057 (SAMEA7471361)
Staphylococcus aureus	PC-BB442-I5	ERS5229058 (SAMEA7471362)
Staphylococcus aureus	PC-BB486-I2	ERS5229059 (SAMEA7471363)
Staphylococcus aureus	PP-BB2079-I	ERS5229063 (SAMEA7471367)
Staphylococcus aureus	PP-BB3938-I	ERS5229071 (SAMEA7471375)
Staphylococcus aureus	PP-BB3956-I	ERS5229072 (SAMEA7471376)
Staphylococcus aureus	PP-BB4507-I	ERS5229073 (SAMEA7471377)
Staphylococcus aureus	PP-BB4614-I	ERS5229075 (SAMEA7471379)
Staphylococcus aureus	PP-BB5936-I	ERS5229084 (SAMEA7471388)
Staphylococcus aureus	PP-BB6944-I	ERS5229093 (SAMEA7471397)
Staphylococcus aureus	PP-BB7632-I	ERS5229095 (SAMEA7471399)
Staphylococcus aureus	PP-BB7955-I	ERS5229096 (SAMEA7471400)
Staphylococcus aureus	PP-BB8010-I	ERS5229097 (SAMEA7471401)
Staphylococcus aureus	PP-BB8048-I	ERS5229099 (SAMEA7471403)
Staphylococcus aureus	PP-BB8061-I	ERS5229100 (SAMEA7471404)
Staphylococcus aureus	RK-BB2000-I	ERS5229102 (SAMEA7471406)
Staphylococcus aureus	ZAT-BB1262-I1	ERS5229103 (SAMEA7471407)
Staphylococcus aureus	ZAT-BB138-I1	ERS5229104 (SAMEA7471408)
Staphylococcus aureus	ZAT-BB2180a-I1	ERS5229109 (SAMEA7471413)
Staphylococcus aureus	ZAT-BB2710-I1	ERS5229113 (SAMEA7471417)
Staphylococcus aureus	ZAT-BB326b-I1	ERS5229115 (SAMEA7471419)

Questionnaire asked to site PIs regarding antibiotic therapy

All sites have an experienced clinical neonatologist and microbiologist. The questionnaire was completed via consultation between their staff and the local pharmacy department. In some countries such as Nigeria, there is a different between state and federal funding which was also captured in the income levels. Income levels was self-reporting data by the mothers but was ratified by the site PIs who have extensive local knowledge on income levels etc.

Name of person completing form …………………………………………………………………
Date………………………

Job role……………………………………………………………………………….

Name of hospital…………………………………………………………………

Country ……………………………………………………………………………….

Region………………………………………………………………………………

1.0 What is the estimated monthly prevalence of clinical diagnosis for neonatal sepsis?

……….

2.0 Do you have the necessary equipment to perform blood cultures? (Y/N – Indicate if this is on site or off site facilities if yes)

……….

2.1 If yes, what is the estimated monthly prevalence of positive blood cultures for neonatal sepsis?

…….

3.0 Please complete the table below:

	Do you have access to the following antibiotics for treatment of neonatal sepsis? (Y/N)	What are the dosing recommendations? (IV, mg/Kg, Interval (Hours)	Estimated cost per dose? ($)
Example	Y	IV, 7.5, 12	5
Ampicillin			
Gentamicin			
Ceftazidime			
Piperacillin/Tazobactam			
Amikacin			
Amoxicillin			
Fosfomycin			
Tigecycline			
Colistin			

4.0 What is the primary empirical treatment for neonatal sepsis at the facility?

………...

5.0 What is the common recommended second line of treatment for neonatal sepsis?

………
………..

6.0 Which causative pathogens for neonatal sepsis are of most concern at the facility? (Delete as appropriate) Klebsiella pneumoniae/Staphylococcus aureas/ Escherichia coli/

7.0 What is the estimated average monthly neonatal morbidity from sepsis?

………
………….

8.0 What is the estimated average total cost for stay, treatment and administration for a neonate with suspected clinical sepsis? ($ per 24 hours?)

………
………..

9.0 How much of the total average cost is invoiced to the patient at the facility? ($ per 24 hours)

………
………

10.0 Are the cost of antibiotics included in the figure above? (Y/N)

………
……….

11.0 Are the patients at the facility charged prescription fees? (If yes- please state amount $)

………
………

12.0 What is the average weekly income for the immediate area the facility serves?

………
……….

13.0 Is the facility public/private/part private (delete as appropriate)

Thank you for taking the time to complete this survey.

Sensitivity analyses, excluding untraceable neonates

Supplementary Table 4. Mortality% associated with different antibiotic therapies (n=476), comparison with untraceable neonates removed from analysis. Mortality increased slightly when untraceable neonates were removed, due to the lower denominator, but similar p values between treatments were found for both analyses.

Antibiotic combination	All neonates		Untraceable neonates removed	
	Total N	Mortality %	Total N	Mortality%
AMP-GEN	111	16.2	96	18.8
AMC-AMK	78	24.4	78	24.4
CTZ-AMK	172	9.3	138	11.6
PIP-TAZ-AMK	115	27.8	90	35.6

X^2 all: N=476, X^2=18.825, df=3, p<0.001
X^2 untraceable neonates removed: N=402, X^2=19.573, df=3, p<0.001

Supplementary Table 5. Mortality% associated with different therapies for empirical dataset only, comparison with untraceable neonates removed from analysis. Mortality increased slightly when untraceable neonates were removed, due to the lower denominator, but similar p values between treatments were found for both analyses.

Antibiotic combination	All neonates		Untraceable neonates removed	
	Total N	Mortality %	Total N	Mortality%
AMP-GEN	78	10.3	72	11.1
AMC-AMK	27	29.6	27	29.6
CTZ-AMK	109	8.3	90	10.0
PIP-TAZ-AMK	76	22.4	58	29.3

X^2 all: N=290, X^2=13.354, df=3, p=0.004
X^2 untraceable neonates removed: N=247, X^2=14.174, df=3, p=0.003

Supplementary Table 6. X^2 statistical results for MIC vs outcome empirical therapy compared to repeated analysis with untraceable neonates removed. Similar p values were found for both analyses.

Antibiotic combination	All neonates				Untraceable neonates removed			
	N	X^2	Df	P value	N	X^2	Df	P-value
AMP-GEN	76	0.804	2	0.669	70	0.718	2	0.698
AMC-AMK	NA -No untraceable neonates							
CTZ-AMK	107	2.818	3	0.421	89	3.162	3	0.367
PIP/TAZ-AMK	76	5.465	3	0.145	58	6.391	3	0.094

Supplementary Table 7. Mann-Whitney U test results for association of PI and outcome, compared to repeated analysis with untraceable neonates removed. Similar p values were found for both analyses.

Dataset	All			Untraceable neonates removed		
	N	Mann Whitney U test statistic	P value (exact)	N	Mann Whitney U test statistic	P-value (exact)
E. coli	22	33.000	0.837	20	30.000	0.892
K. pneumoniae	55	178.500	0.517	46	189.000	0.549
S. aureus	33	113.000	0.954	28	91.000	1.000

Supplementary Table 8. Mann-Whitney U test results for association of VFs and outcome, compared to repeated analysis with untraceable neonates removed. Similar p values were found for both analyses.

Dataset	All			Untraceable neonates removed		
	N	Mann Whitney U test statistic	P value	N	Mann Whitney U test statistic	P-value
E. coli	22	12.500	0.042	20	9.500	0.029
K. pneumoniae	55	188.000	0.663	46	173.000	0.870
S. aureus	33	128.000	0.630	28	108.000	0.408

Supplementary Table 9. Most frequent antibiotics given per BARNARDS sites. Cells in blue indicate the most frequently prescribed antibiotic therapy combination per site.

**Site**	**Top empirical antibiotics used**				
BC -Bangladesh	Ceftazidime	Amikacin	Cefotaxime	Ampicillin	Meropenem
BK -Bangladesh	Ampicillin	Gentamicin	Cefotaxime	Ceftazidime	Amikacin
Ethiopia	Ampicillin	Gentamicin	Cefotaxime	Ceftazidime	Vancomycin
India	Piperacillin-tazobactam	Netilmicin	Ofloxacin	Meropenem	Cefixime
NK -Nigeria	Amoxicillin	Gentamicin	Amikacin	Ceftazidime	Cloxacillin
NN -Nigeria	Amoxicillin	Amikacin	Ceftazidime	Ceftriaxone	Cefuroxime
NW -Nigeria	Amoxicillin	Ceftazidime	Ampicillin	Cloxacillin	Gentamicin
PC -Pakistan	Piperacillin-tazobactam	Amikacin	Cefotaxime	Meropenem	Vancomycin
PP -Pakistan	Piperacillin-tazobactam	Amikacin	Cefotaxime	Vancomycin	Imipenem
RK -Rwanda	Ampicillin	Gentamicin	Benzylpenicillin	Cefotaxime	Ciprofloxacin
RU -Rwanda	Ampicillin	Gentamicin	Cefotaxime	Meropenem	Ciprofloxacin
South Africa	Ampicillin	Gentamicin	Meropenem	Piperacillin-tazobactam	Amikacin
**Top total 5 antibiotics used**	Ampicillin	Gentamicin	Ceftazidime	Amikacin	Piperacillin-tazobactam

Supplementary Table 10. Number of each combination per country.

Antibiotic combination	Country						Total
	Bangladesh	Ethiopia	Nigeria	Pakistan	Rwanda	South Africa	
AMP-GEN	6	29	2	0	51	23	111
CTZ-AMK	157	0	14	1	0	0	172
AMC-AMK	0	0	75	3	0	0	78
PIP/TAZ-AMK	0	0	0	108	0	7	115

Supplementary Table 11. Number of each combination per country for the n=290 subset of neonates that only received one of these treatments with no change in therapy

Antibiotic combination	Country						Total
	Bangladesh	Ethiopia	Nigeria	Pakistan	Rwanda	South Africa	
AMP-GEN	5	29	0	0	40	4	78
CTZ-AMK	107	0	1	1	0	0	109
AMC-AMK	0	0	27	0	0	0	27
PIP/TAZ-AMK	0	0	0	76	0	0	76

Supplementary Table 12. Numbers included in this sub-study, including all isolates and all prescriptions recorded for each antibiotic combination, with information on associated cohort and clinical variables.

		Antibiotic therapy				Total	Missing data
		AMP-GEN	CTZ-AMK	AMC-AMK	PIP/TAZ-AMK		
Total number		111	172	78	115	476	-
Cohort	Inborn	69	34	37	102	242	-
	Non-inborn	42	138	41	13	234	
Gender	Male	46	116	44	46	252	70
	Female	43	52	34	25	154	
Premature	Yes	65	24	36	59	184	4
	No	42	148	42	56	288	
Caesarean	Yes	44	70	33	73	220	7
	No	60	102	45	42	249	
Onset of sepsis	EOS	61	44	20	53	178	45
	LOS	49	121	54	29	253	
Type of organism	GPB	9	9	16	24	58	-
	GNB	102	163	62	91	418	

Supplementary Table 13. Clinical information for neonates included in this study, compared to total numbers of culturally confirmed neonates in the main BARNARDS project. Similar numbers can be seen for cohort

enrolment, gender, prevalence of premature neonates and Caesarean section births. There was a higher % of LOS cases included in this subset compared to the main BARNARDS study. The main BARNARDS study originally only focused on GNB due to a higher burden of resistance. Therefore, GPB cases of sepsis were recorded, but the isolates were not sent until later in the study. This is why the WGS for GPB is much lower in the main BARNARDS study and therefore, a low rate of GPB could be incorporated into this dataset due to availability of WGS data.

		Dataset in this study (n=442)	Missing data, this study	Total (%) from main BARNARDS study	Missing data from main BARNARDS study	X^2 statistic	P-value
Total number enrolled with culturally confirmed sepsis		442 neonates<br>457 isolates	-	2483 neonates<br>2620 isolates	-		
Cohort	Inborn<br>Non-inborn	221 (50%)<br>221 (50%)	-	1111 (44.7%)<br>1372 (55.3%)	-	0.409	0.463
Gender	Male<br>Female	231 (52.26%)<br>145 (32.81%)	66 (14.93%)	1018 (41.0%)<br>733 (29.5%)	732 (29.5%)	1.256	0.239
Premature	Yes<br>No	161 (36.43%)<br>277 (62.67%)	4 (0.90%)	787 (31.7%)<br>1630 (65.7%)	66 (2.7%)	2.945	0.086
Caesarean	Yes<br>No	198 (44.79%)<br>238 (53.85%)	6 (1.36%)	972 (39.2%)<br>1387 (55.9%)	124 (5.0%)	2.679	0.102
Onset of sepsis	EOS<br>LOS	167 (37.78%)<br>232 (52.49%)	43 (9.73%)	1429 (57.55%)<br>829 (33.39%	228 (9.2%)	64.930	<0.0001
Type of organism per 457/2620 isolates	GPB<br>GNB	55<br>402	-	1266 (48.32%)<br>130 WGS<br>1038 (39.62%)<br>916 WGS	294 + 21 fungal isolates (12.06%)	0.046*	0.898

*X^2 for type of organism was based on total sequenced at Cardiff University. X^2 carried comparing type of organism to the total recorded in BARNARDS was significant. However, as described in the table legend above, most GPB isolates were not received at Cardiff University.

Supplementary Table 14. Number of cases for which each antibiotic combination was prescribed. Mortality rate for each of these combinations has also been reported.

Antibiotic treatment	Number of prescriptions including treatment	Mortality rate (%)
**Ampicillin + Gentamicin**	111	16.2
**Amoxicillin + Amikacin**	78	24.4
**Ceftazidime + Amikacin**	172	9.3
**Piperacillin-tazobactam + Amikacin**	115	27.8

Supplementary Table 15. Number of cases each empirical therapy combination was prescribed with no change in antibiotics reported to have been prescribed following initial therapy combination. Mortality rate for each of these combinations has also been reported. Cases where additional antibiotics were reported to be prescribed simultaneously were excluded.

Empirical therapy	Number of cases	Mortality rate (%)
**Ampicillin + Gentamicin**	78	10.3
**Amoxicillin + Amikacin**	27	29.6
**Ceftazidime + Amikacin**	109	8.2
**Piperacillin-Tazobactam + Amikacin**	76	22.4

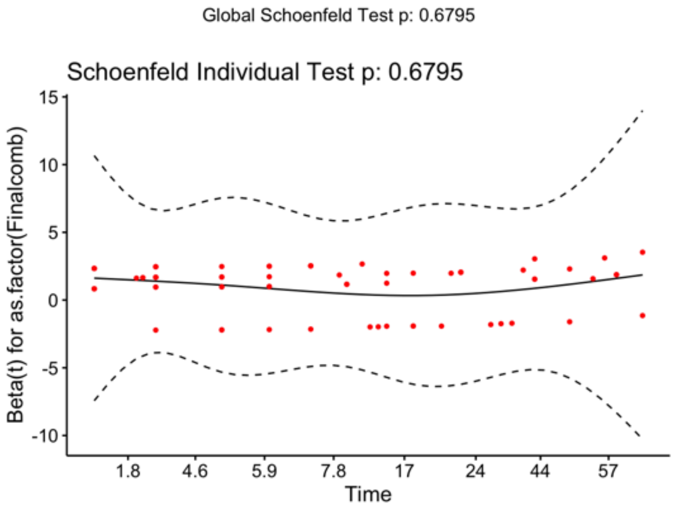
Global Schoenfeld Test p: 0.6795
Schoenfeld Individual Test p: 0.6795
Beta(t) for as.factor(Finalcomb)
Time

A

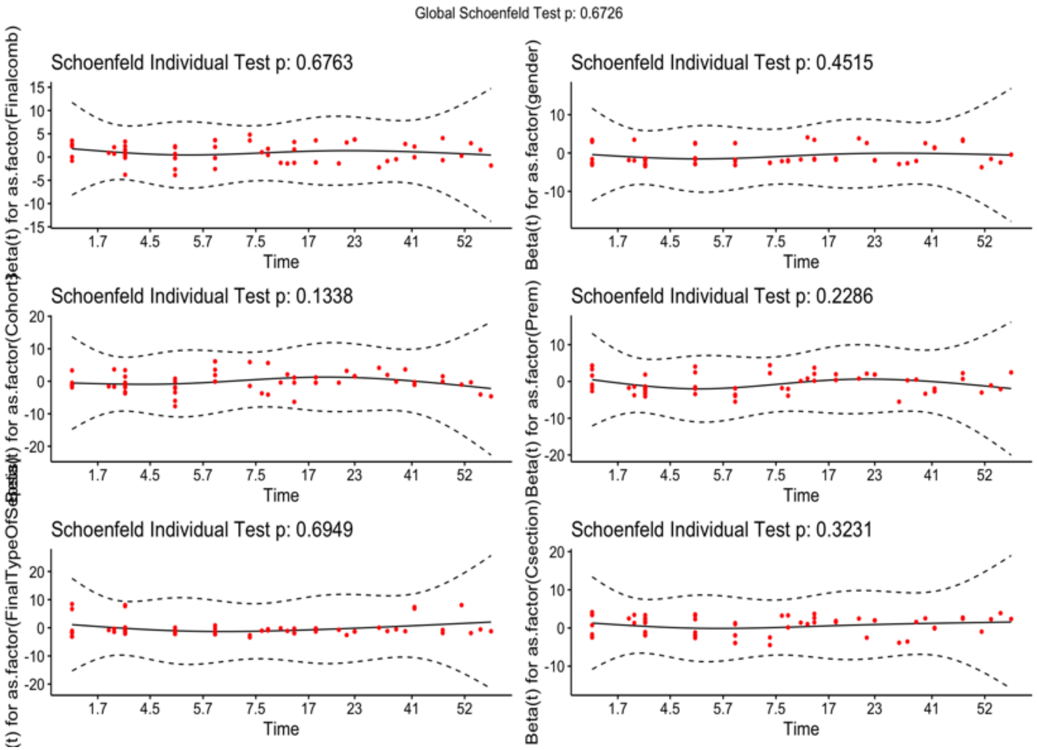
Global Schoenfeld Test p: 0.6726
Schoenfeld Individual Test p: 0.6763
Beta(t) for as.factor(Finalcomb)
Schoenfeld Individual Test p: 0.4515
Beta(t) for as.factor(gender)
Schoenfeld Individual Test p: 0.1338
Beta(t) for as.factor(Cohort)
Schoenfeld Individual Test p: 0.2286
Beta(t) for as.factor(Prem)
Schoenfeld Individual Test p: 0.6949
Schoenfeld Individual Test p: 0.3231
Beta(t) for as.factor(Csection)
Time

B

C

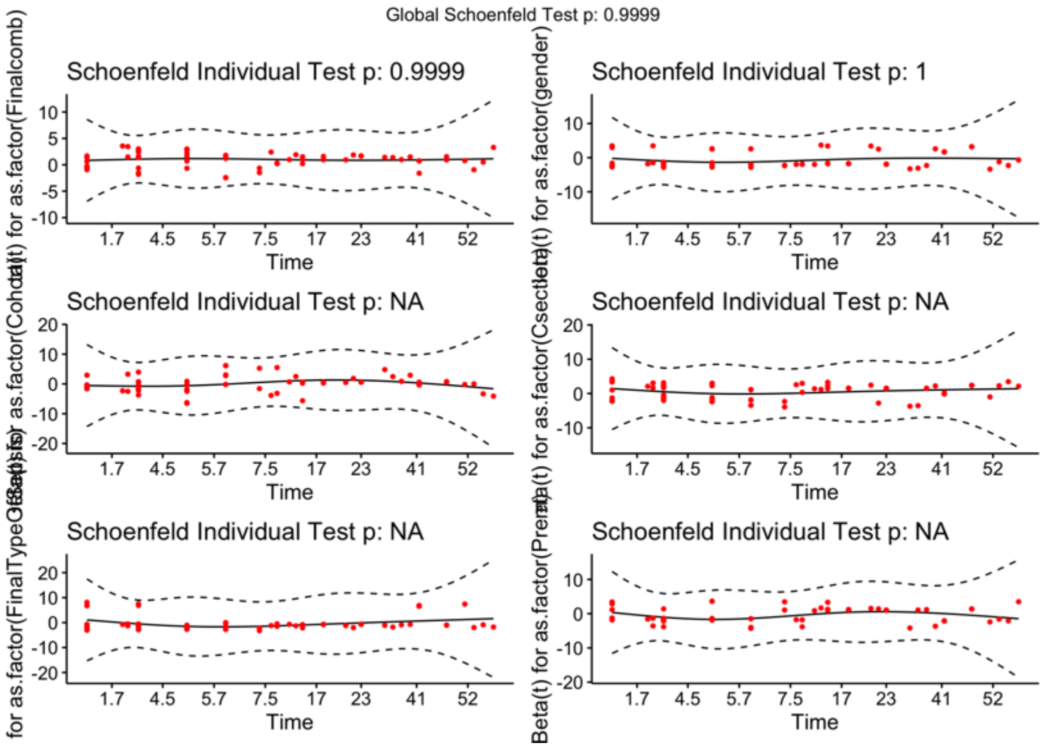

Supplementary Figure 2. Schoenfeld residual plots for A. Unadjusted and B. Adjusted Cox proportional hazard regression models carried out on n=476. Type of sepsis (EOS/LOS) was stratified within the adjusted model to ensure that Cox proportional hazard assumptions were met. C. Mixed-effect model: PH assumptions displayed NA for multiple variables, showing that the mixed-effect model with country incorporated as a random effect did not fit the data well, due to the dispersion of the data between countries.

Supplementary Table 16. Cox regression proportional hazards results for overall data, n=476 for unadjusted and adjusted models as per clinical information provided in Supplementary Table 12. Adjusted analysis n=368 (108 observations deleted due to missingness), number of events=63. EOS/LOS was stratified in this model to ensure proportional hazard assumptions were met. Associated graphs provided below. No confidence intervals could be obtained for the mixed effect model accounting for country variation due to the poor fit of the model.

	Unadjusted				Adjusted for clinical factors				Mixed effects model, country variation			
	HR	95% CI		P value	HR	95% CI		P value	HR	95% CI		P-value
		Lower	Upper			Lower	Upper			Lower	Upper	
AMP-GEN (1)												
CTZ-AMK (2)	0.338	0.169	0.675	0.002	0.316	0.139	0.718	0.006	2.092			0.450
AMC-AMK (3)	1.281	0.666	2.467	0.458	1.186	0.595	2.237	0.628	2.760			0.270
PIP/TAZ-AMK (4)	1.669	0.851	3.275	0.136	1.894	0.883	4.063	0.101	0.342			0.190

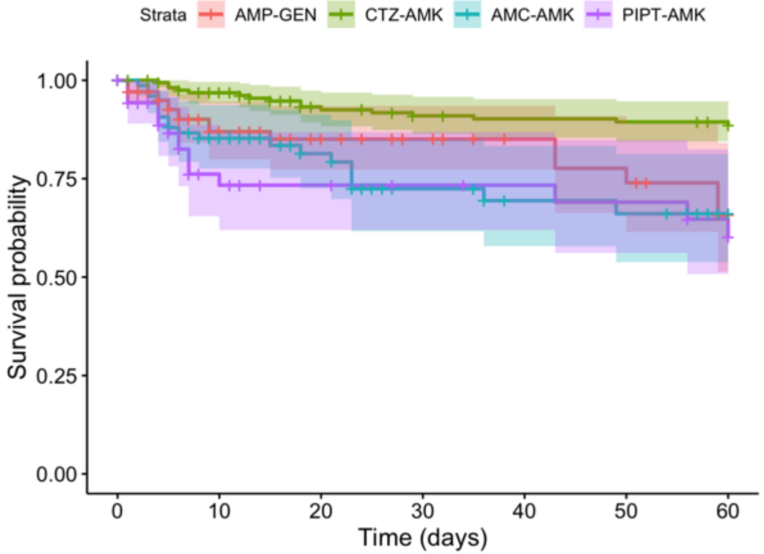

A

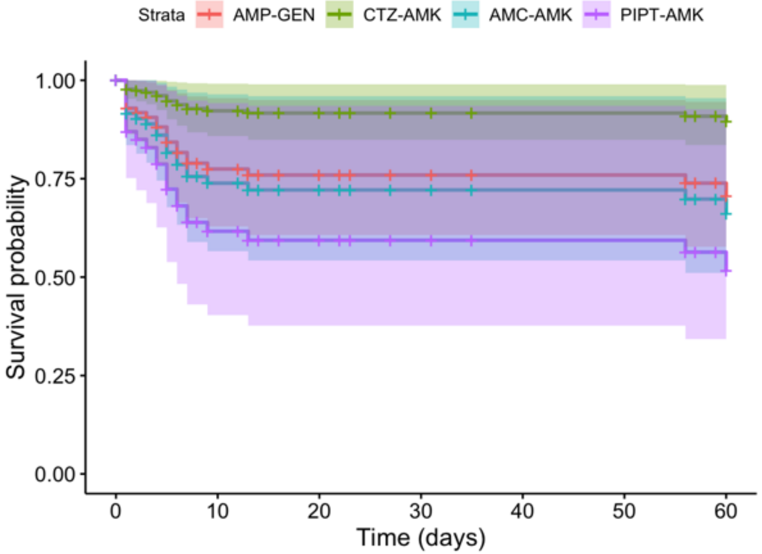

B

Supplementary Figure 3. Cox regression proportional hazards results displayed as graphs for overall data, n=476 per antibiotic therapy given for A) unadjusted and B) adjusted models as per clinical information provided in Supplementary Table 12. For adjusted graph, clinical information was set at: gender: male, cohort: inborn, Type of sepsis: EOS, sepsis pathogen type: GNB, C-section: no, premature: no. Survival curves were made in R Studio, with the survival and survminer packages.

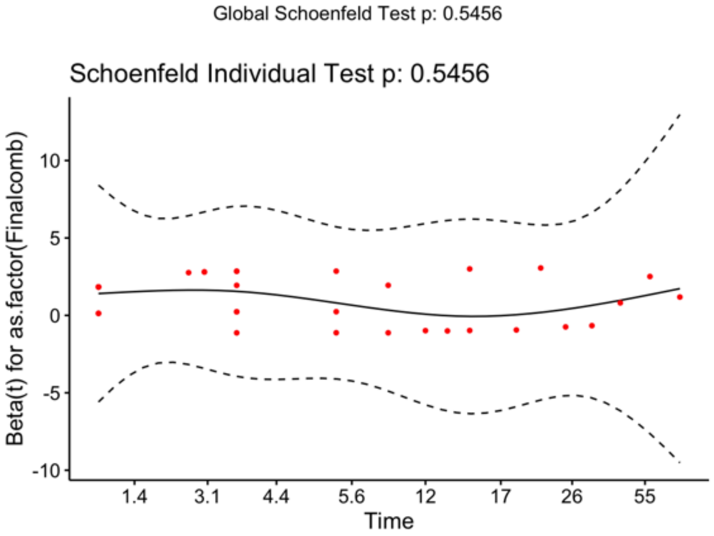
Global Schoenfeld Test p: 0.5456
Schoenfeld Individual Test p: 0.5456
Beta(t) for as.factor(Finalcomb)
Time

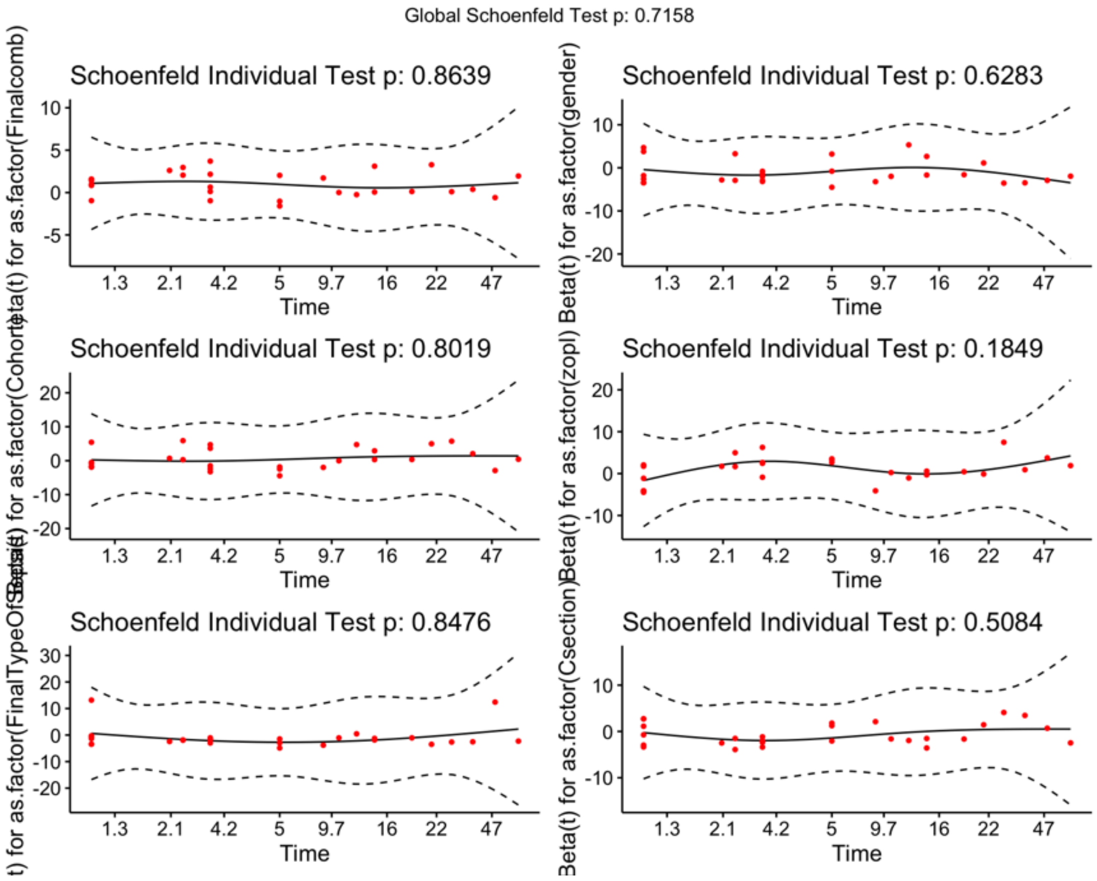
Global Schoenfeld Test p: 0.7158
Schoenfeld Individual Test p: 0.8639
Schoenfeld Individual Test p: 0.6283
Beta(t) for as.factor(gender)
Schoenfeld Individual Test p: 0.8019
Schoenfeld Individual Test p: 0.1849
Beta(t) for as.factor(zopl)
Schoenfeld Individual Test p: 0.8476
Schoenfeld Individual Test p: 0.5084
Time

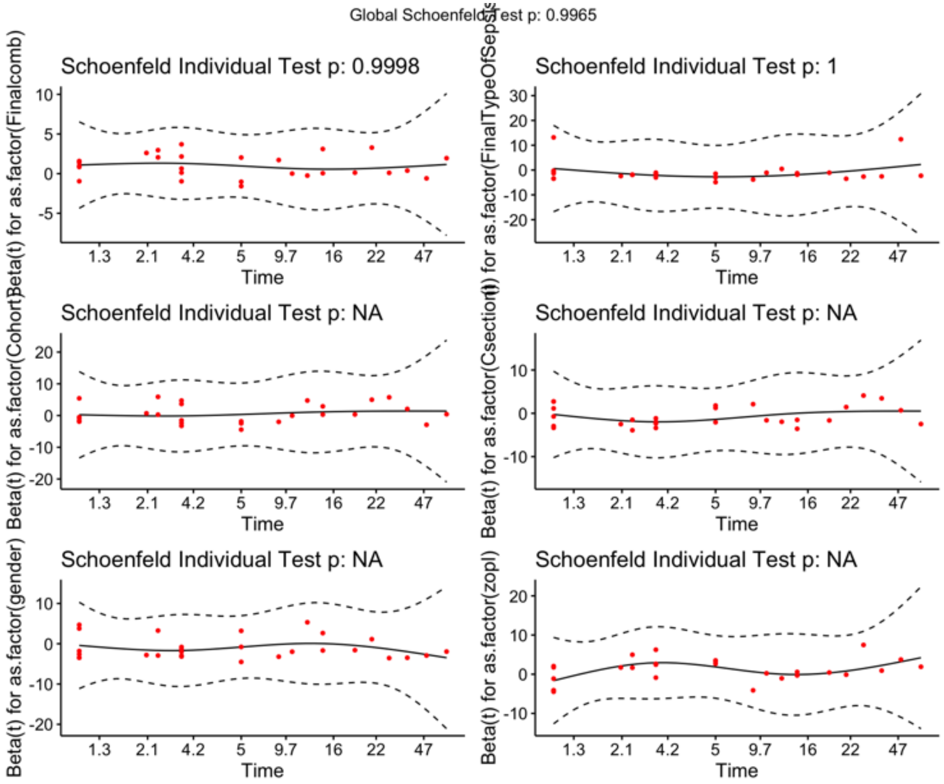

Supplementary Figure 4. Schoenfeld residual plots for A. Unadjusted and B. Adjusted Cox proportional hazard regression models carried out on n=290. Type of sepsis (EOS/LOS) was stratified within the adjusted model to ensure that Cox proportional hazard assumptions were met. C. Mixed-effect model: PH assumptions displayed NA for multiple variables in the mixed-effect model with country incorporated as a random effect, showing that this model did not fit the data well, due to the dispersion of the data between countries.

Supplementary Table 17. Cox regression proportional hazards results for empirical dataset, n=290 for unadjusted and adjusted models as per clinical information provided in Supplementary Table 12. Adjusted model n=210 (80 observations deleted due to missingness), events, n=28). EOS/LOS was stratified in this model to ensure proportional hazard assumptions were met. No confidences intervals were available for the mixed effect model. Associated graphs provided below.

	Unadjusted				Adjusted for clinical factors				Mixed effects model, country variation			
	HR	95% CI		P value	HR	95% CI		P value	HR	95% CI		P-value
		Lower	Upper			Lower	Upper			Lower	Upper	
AMP-GEN (1)												
CTZ-AMK (2)	0.572	0.211	1.566	0.279	0.511	0.155	1.687	0.271	0.511			0.270
AMC-AMK (3)	2.900	1.050	8.011	0.040	5.557	1.707	18.083	0.004	5.557			0.004
PIP/TAZ-AMK (4)	2.002	0.701	5.713	0.195	3.018	0.928	9.813	0.066	3.018			0.066

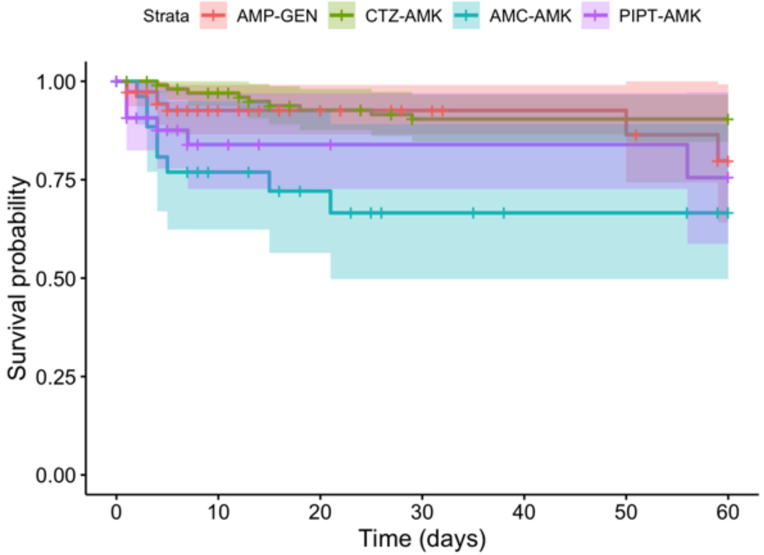

A

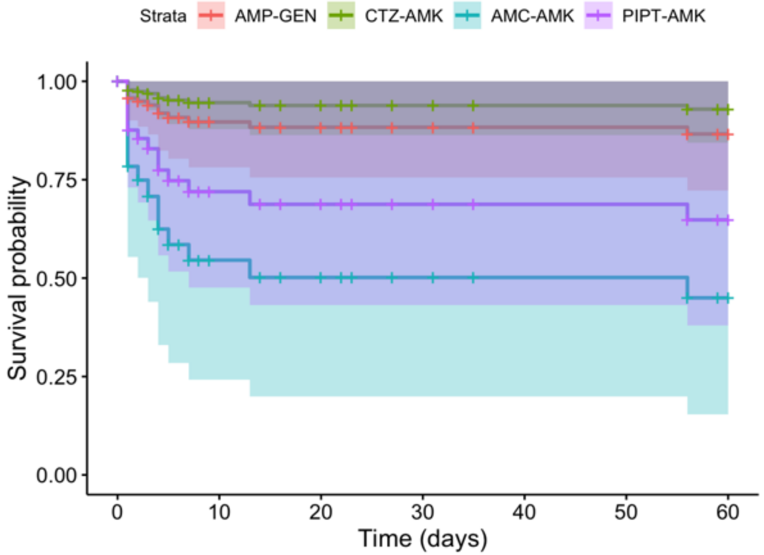

B

Supplementary Figure 5. Cox regression proportional hazards results displayed as graphs for empirical dataset with survival, n=290 per antibiotic therapy given for A) unadjusted and B) adjusted models as per clinical information provided in Supplementary Table 11. For adjusted graph, clinical information was set at: gender: male, cohort: inborn, Type of sepsis: EOS, sepsis pathogen type: GNB, C-section: no, premature: no. Strata 1=AMP-GEN; 2=CTZ-AMK; 3=AMC-AMK; 4=PIP/TAZ-AMK. Survival curves were made in R Studio, with the survival and survminer packages.

Supplementary Table 18. MIC_{50} and MIC_{90} results from for Gram-negative isolates included in this study (n=401). Highest concentration tested on all isolates input.

**Antibiotic**	**MIC_{50}**	**MIC_{90}**
Ampicillin	>32	>32
Amoxicillin-clavulanate	>32	>32
Piperacillin-tazobactam	4	>32
Ceftriaxone	>4	>4
Cefotaxime	>4	>4
Ceftazidime	>4	>4
Cefepime	>4	>4
Imipenem	1	>8
Meropenem	1	>8
Ertapenem	0.25	>2
Aztreonam*	>4	>4
Gentamicin	>8	>8
Amikacin	4	>32
Tobramycin	>8	>8
Tigecycline	1	2
Minocycline	4	>4
Fosfomycin	16	64
Levofloxacin	0.5	>4
Ciprofloxacin	0.5	>2
Colistin	1	>8

Supplementary Table 19. MIC50 and MIC90 results from MIC testing for Gram-positive isolates tested that were included in this study (n=56). Highest concentration tested on all isolates input.

**Antibiotic**	**MIC_{50}**	**MIC_{90}**
Ampicillin	4	64
Oxacillin	2	>8
Flucloxacillin	1	>8
Levofloxacin	0.5	>4
Ciprofloxacin	0.5	>4
Gentamicin	0.5	>4
Amikacin	4	8
Tobramycin	0.5	>4
Tigecycline	0.25	0.5
Minocycline	0.25	1
Rifampicin	0.03	0.03
Vancomycin	1	2
Azithromycin	4	>8
Linezolid	2	4

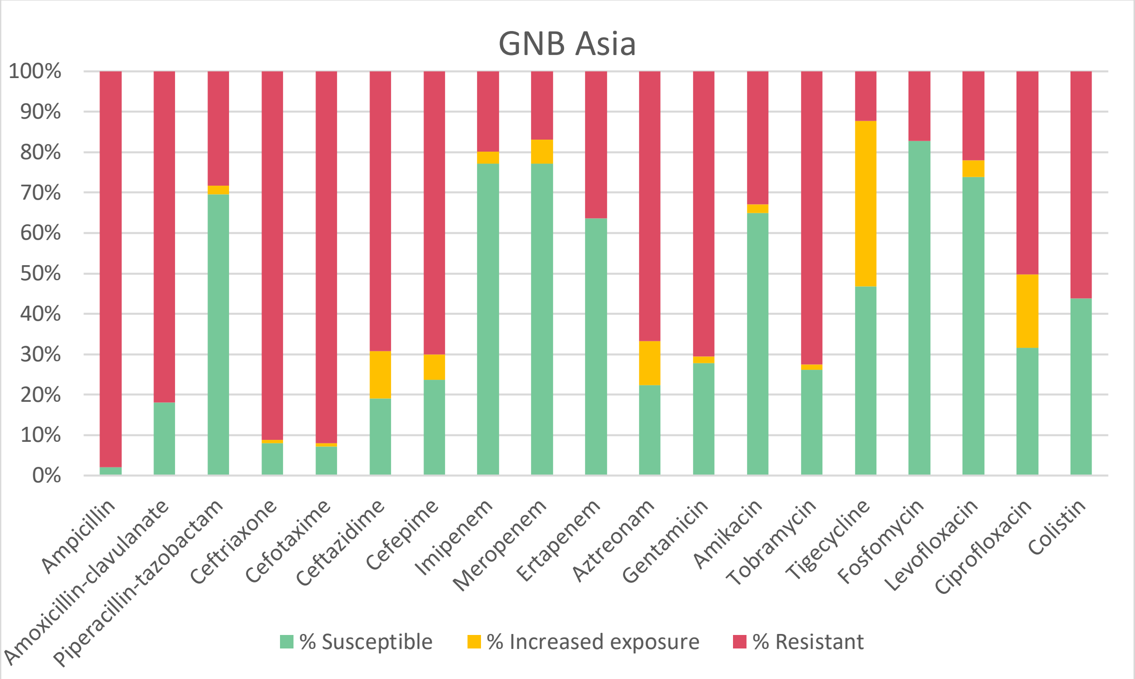

A

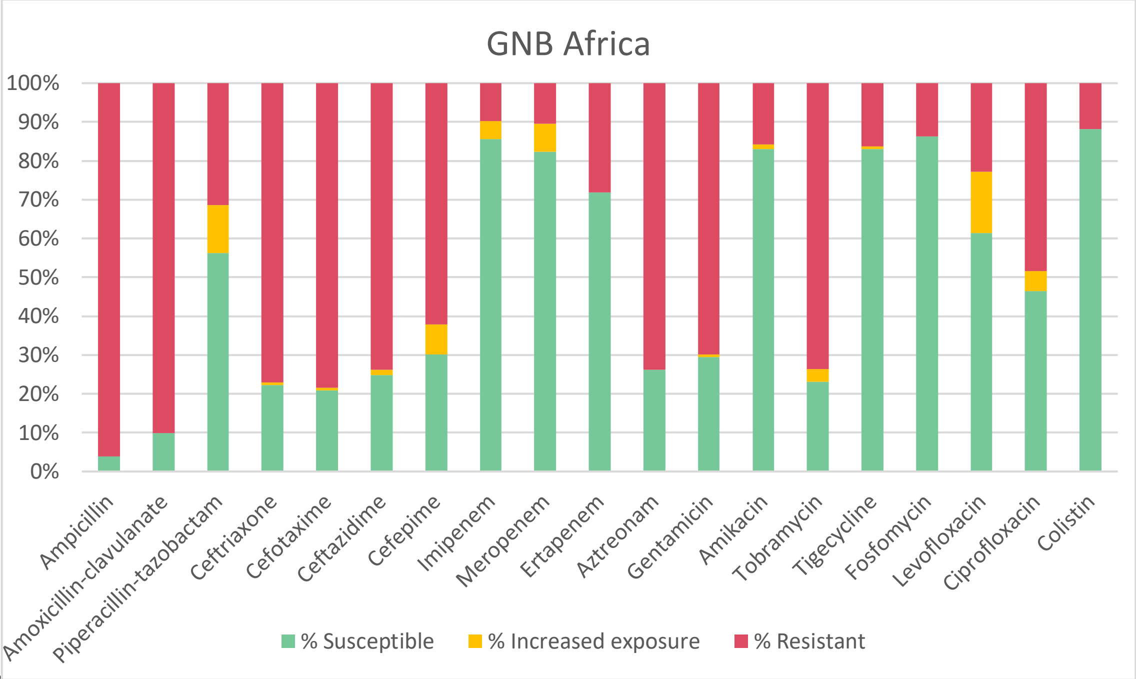

B

Supplementary Figure 6. Antibiotic resistance profiles according to EUCAST v9.0 (2019)[1] breakpoints for Gram-negative isolates within this subset from sites in A. Asia (n=237) and B. Africa (n=153). Similar resistance profiles can be seen per continent for most antibiotics. However, differences are seen for tigecycline, as resistance for this antibiotic was determined via ECOFF values and therefore very reliant on species diversity in a continent. Colistin also displayed a higher prevalence of resistance in Asia. However, this was due to higher frequency of intrinsically resistant species, including *S. marcescens*.

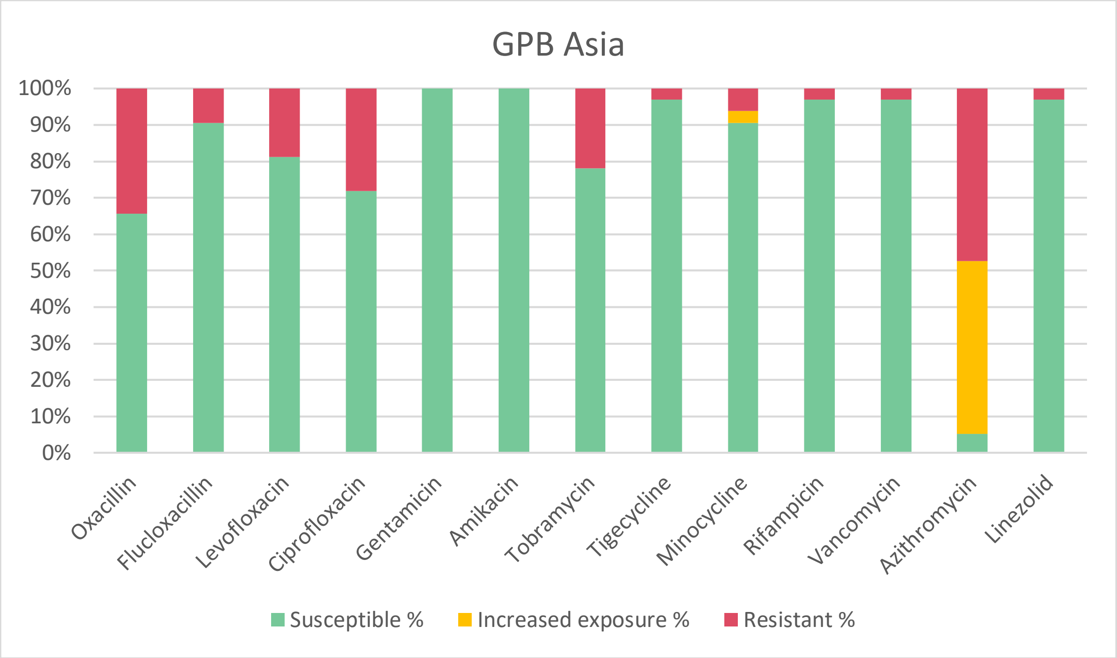

A

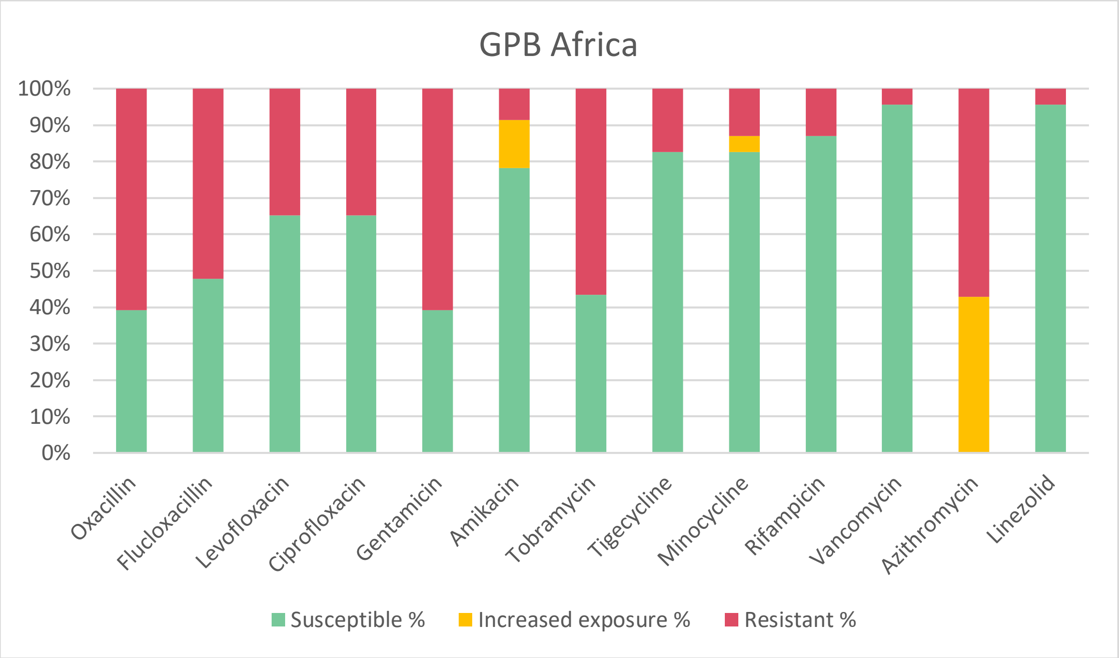

B

Supplementary Figure 7. Antibiotic resistance profiles, according to EUCAST v9.0 (2019)[1] breakpoints for Gram-positive bacteria with MIC profiles within this sub-set from sites in A. Asia (n=32) and B. Africa (n=23). Overall, lower resistance profiles were demonstrated, with exception of high Azithromycin resistance in both continents. Higher levels of resistance were seen in isolates in from clinical sites in Africa for most antibiotics tested.

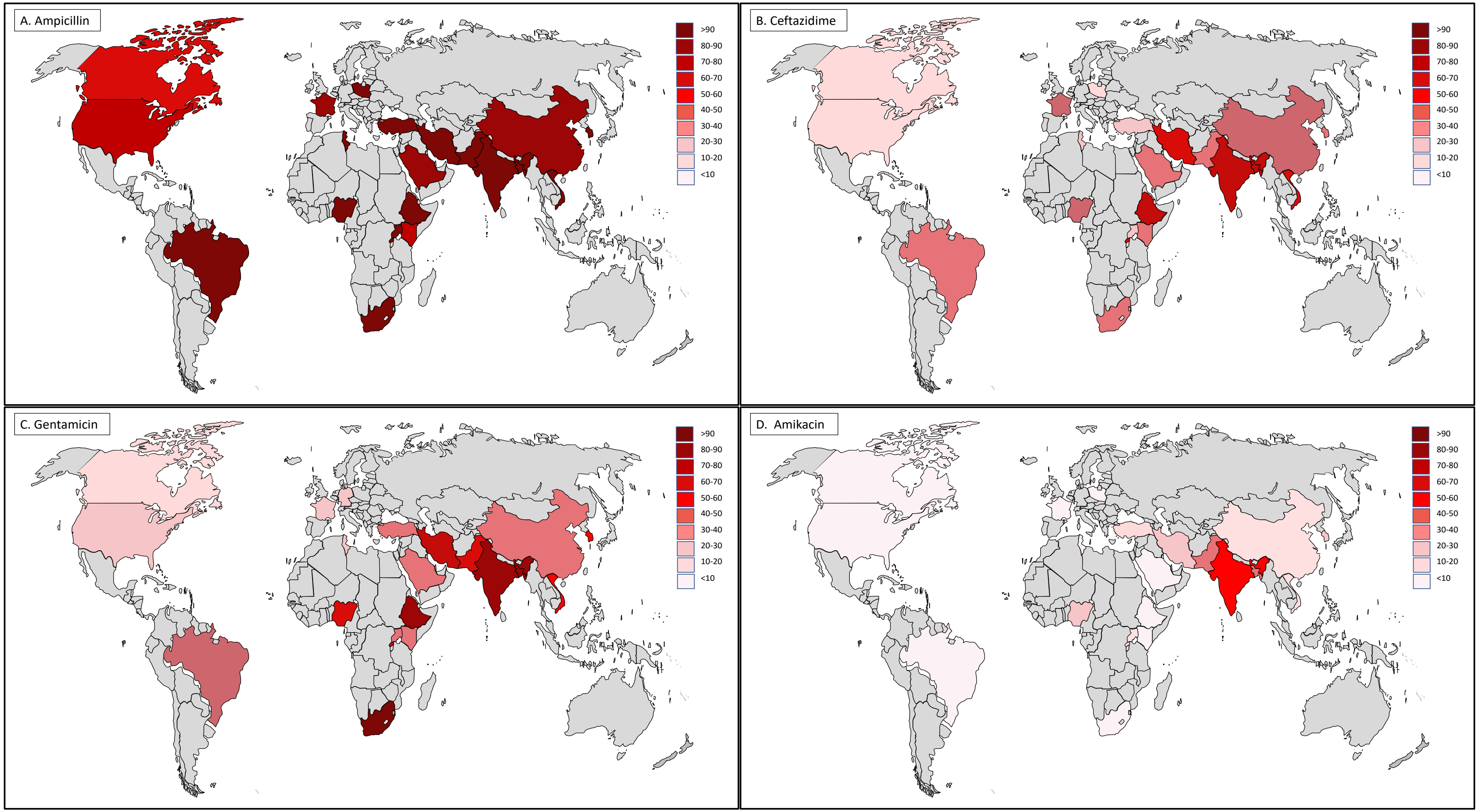

Supplementary Figure 8. Global heatmaps showcasing prevalence of resistance found against A. ampicillin; B. Ceftazidime; C. Gentamicin; and D. Amikacin. Heatmaps incorporate results from a literature review with terms “Enterobacteriaceae antibiotic resistance” and “country”, “neonatal sepsis” and “sepsis”. Where possible, studies possessing over 100 isolates were included; where national reference reporting was available i.e. Canada, the data was taken from the most recent datasets. Not all countries were searched using the above terms and therefore, the heat maps are not meant to show a comprehensive review but a reflection on the global picture of ampicillin versus ceftazidime, and gentamicin versus amikacin.[38-51] The data for Bangladesh, Indian, Pakistan, Ethiopia, Rwanda, South Africa and Nigeria were taken from the main BARNARDS study.

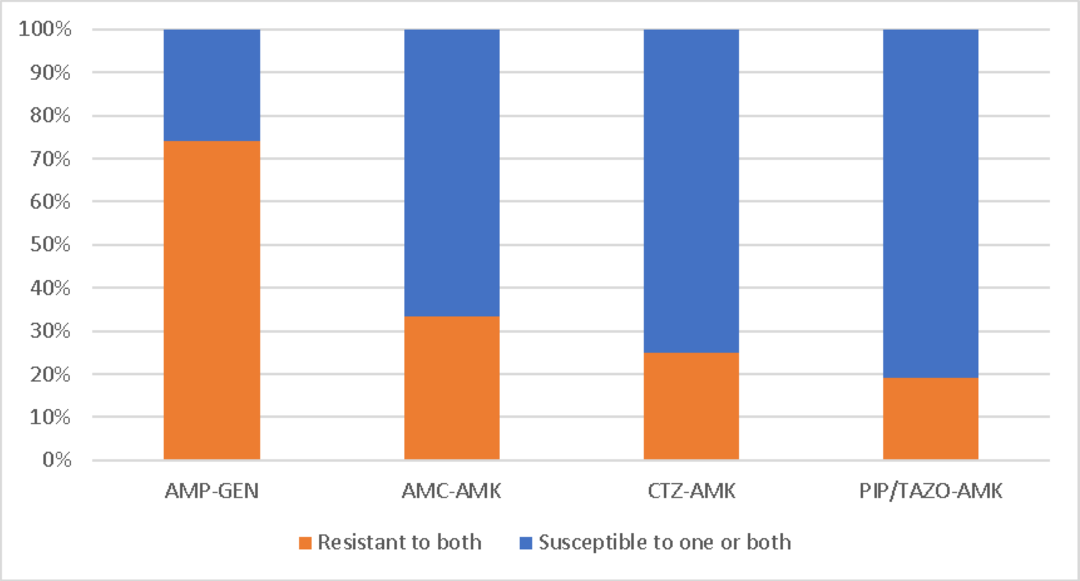

Supplementary Figure 9. Proportion of resistance (%) to both antibiotics included in antibiotic combinations significantly differed between treatment combinations ($X^2(3, N= 461) = 91.226, p=<0.001$) with highest pathogen resistance to both antibiotics found against Ampicillin & Gentamicin (74.1%), comparative to resistance seen for ceftazidime & amikacin, piperacillin/tazobactam & amikacin and amoxicillin & amikacin (24.9, 19.1 and 33.3% respectively). Isolates susceptible to one of the antibiotics in the treatment were included with isolates suceptible to both antibiotics, as one antibiotic would provide coverage.

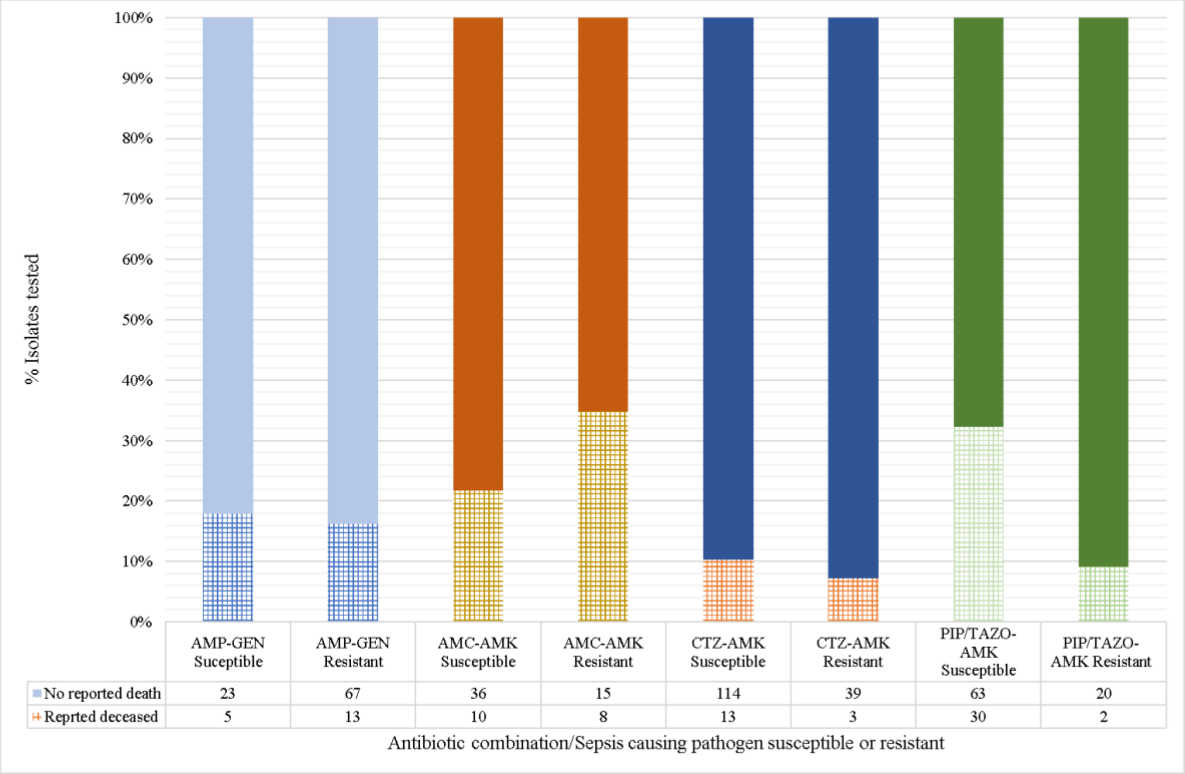

	AMP-GEN Suceptible	AMP-GEN Resistant	AMC-AMK Susceptible	AMC-AMK Resistant	CTZ-AMK Susceptible	CTZ-AMK Resistant	PIP/TAZO-AMK Susceptible	PIP/TAZO-AMK Resistant
No reported death	23	67	36	15	114	39	63	20
Reprted deceased	5	13	10	8	13	3	30	2

Supplementary Figure 10. Proportion of infant outcome based on susceptibility of the pathogen to common empirical treatment. Per antibiotic combination, the sepsis causing pathogens susceptibility or resistance to treatment had no significant effect on patient outcome (Ampicillin & Gentamicin, $X^2(1, N = 108) = 0.39$, p =0.844; Ceftazidime & Amikacin, $X^2(1, N = 169) = 0.352$, p =0.553;); Amoxicillin & Amikacin, $X^2(1, N = 69) = 1.353$, p =0.245). With the exception of Piperacillin/Tazobactam & Amikacin, where infants with pathogens susceptible to this treatment have a significantly higher proprotion of infant mortality $X^2(1, N = 115) = 4.755$, p =0.029.

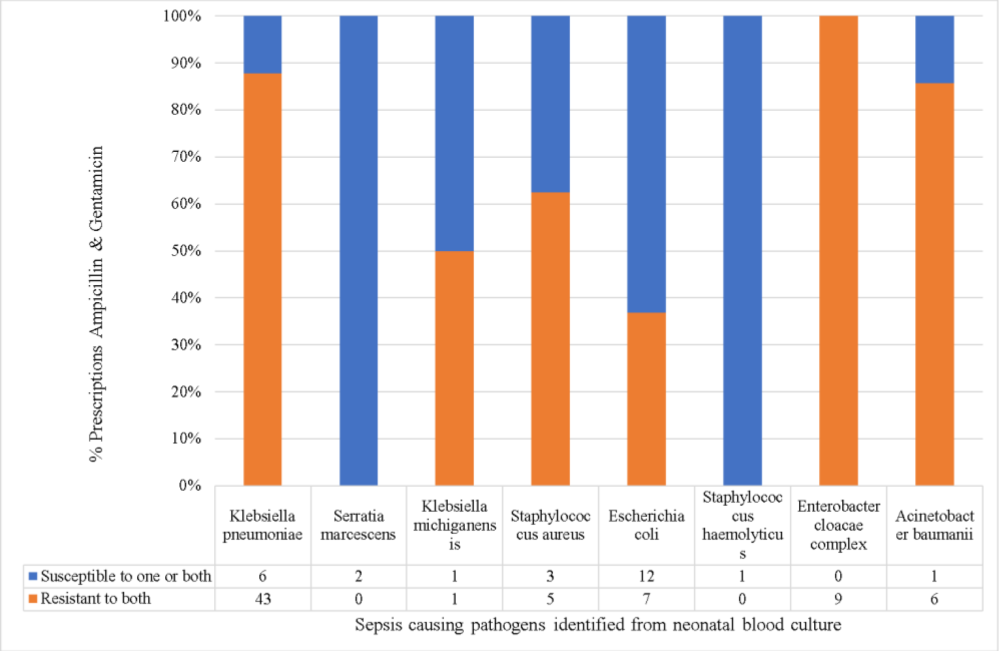
100%
90%
80%
70%
60%
50%
40%
30%
20%
10%
0%
% Prescriptions Ampicillin & Gentamicin
Klebsiella pneumoniae
Serratia marcescens
Klebsiella michiganensis
Staphylococcus aureus
Escherichia coli
Staphylococcus haemolyticus
Enterobacter cloacae complex
Acinetobacter baumanii
Susceptible to one or both
6
2
1
3
12
1
0
1
Resistant to both
43
0
1
5
7
0
9
6
Sepsis causing pathogens identified from neonatal blood culture
A

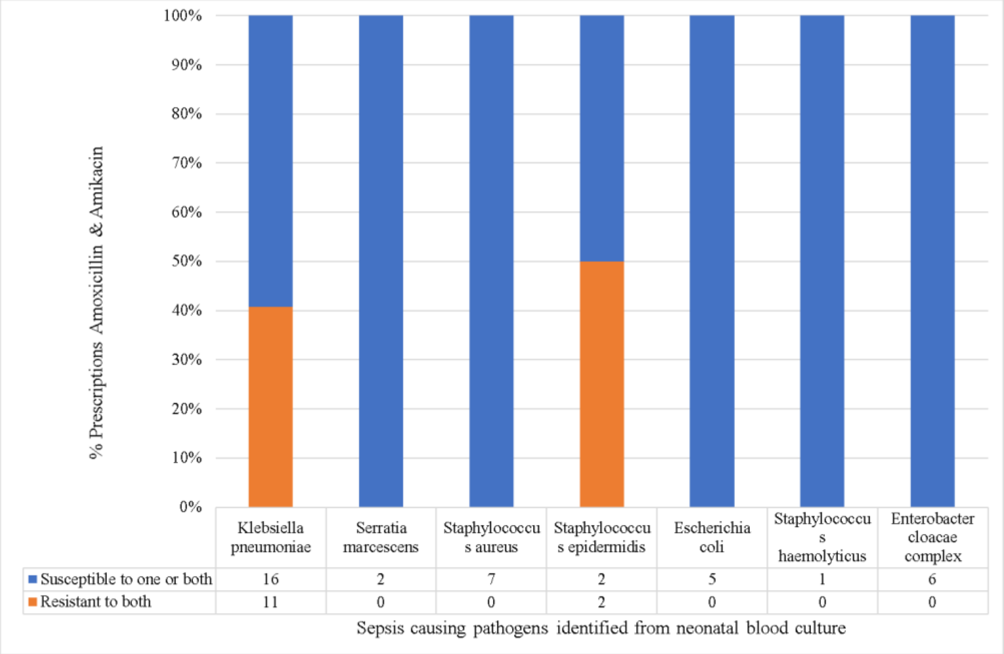
100%
90%
80%
70%
60%
50%
40%
30%
20%
10%
0%
% Prescriptions Amoxicillin & Amikacin
Klebsiella pneumoniae
Serratia marcescens
Staphylococcus aureus
Staphylococcus epidermidis
Escherichia coli
Staphylococcus haemolyticus
Enterobacter cloacae complex
Susceptible to one or both
16
2
7
2
5
1
6
Resistant to both
11
0
0
2
0
0
0
Sepsis causing pathogens identified from neonatal blood culture
B

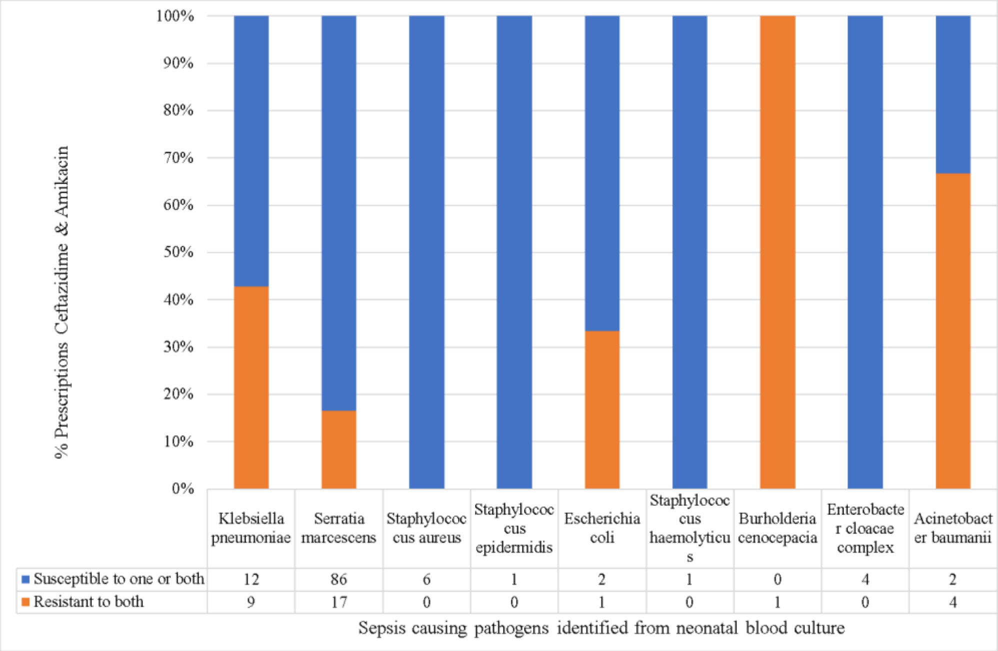

C

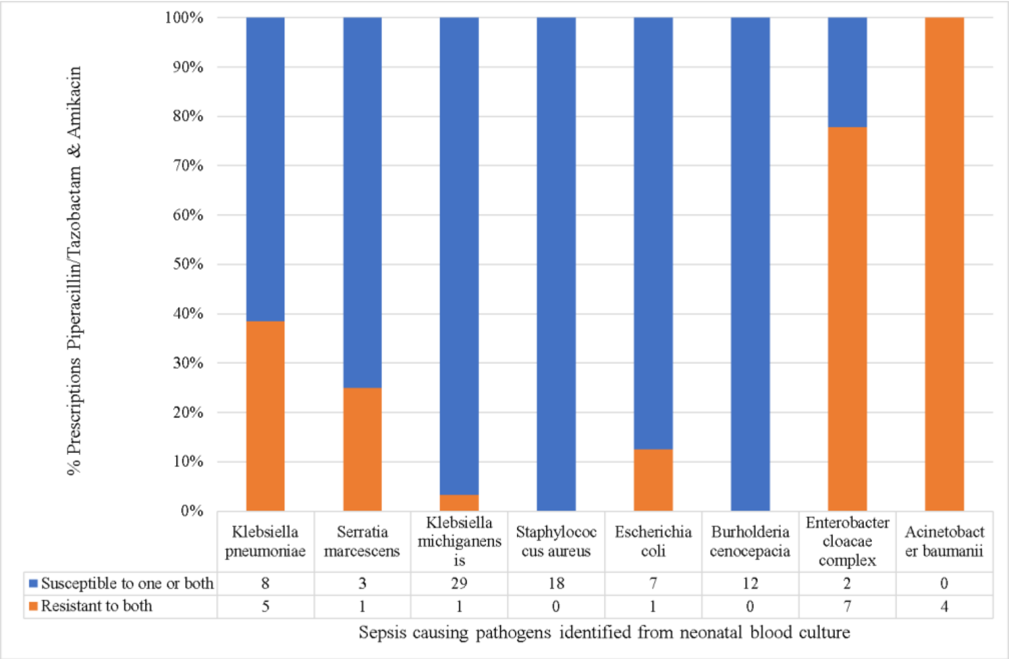

D

Supplementary Figure 11. Proportion of susceptibility of sepsis causing pathogens isolated from positive neonatal blood cultures to the treatment given to the infant. Only species included in the top 10 most occurring sepsis pathogens across sites have been included. A) AMP-GEN; B) AMC-AMK; C) CTZ-AMK; D) PIP/TAZ-AMK.

Supplementary Table 20. Top ten species overall from BARNARDS with WGS IDs, vs those selected for this study. The 476 subset included 418 (87.82%) Gram-negative species and 58/476 (12.18%) Gram-positive species, while the overall dataset with WGS IDs included 916/1,046 (87.57%) Gram-negative and 130 (12.43%) Gram-positive species. This difference was found not to be statistically different (X^2=0.0179, p=0.894). Both datasets had the same top ten species, in a similar order of occurrence.

**From BARNARDS overall with WGS data**		**From 476 subset**	
**Species ID**	**Number /1,046**	**Species ID**	**Number /476**
Klebsiella pneumoniae	258 (24.67%)	*Serratia marcescens*	112 (23.53%)
Serratia marcescens	151 (14.44%)	*Klebsiella pneumoniae*	110 (23.11%)
Klebsiella michiganensis	117 (11.19%)	*Staphylococcus aureus*	40 (8.40%)
Staphylococcus aureus	100 (9.56%)	*Escherichia coli*	35 (7.35%)
Escherichia coli	75 (7.17%)	*Klebsiella michiganensis*	32 (6.72%)
Enterobacter cloacae complex	57 (5.45%)	*Enterobacter cloacae complex*	28 (5.88%)
Burkholderia cenocepacia	56 (5.35%)	*Acinetobacter baumannii*	18 (3.78%)
Acinetobacter baumannii	38 (3.63%)	*Burkholderia cenocepacia*	13 (2.73%)
Pseudomonas aeruginosa	23 (2.20%)	*Ralstonia mannitolytica*	13 (2.73%)
Ralstonia mannitolytica	20 (1.91%)	*Pseudomonas aeruginosa*	9 (1.89%)
Other species	151 (31.72%)	Other species	66 (13.87%)

Supplementary Table 21. Chi square tests undertaken for all top ten species listed above to determine any significant differences in dispersion of species included in the subset included in this study. Overall, the 476 subset included in this study was representative of the overall dataset. However, a statistical difference was found in the number of *S. marcescens K. michiganensis*, and *B. cenocepacia*. Despite this, all top ten species were the same beween datasets and numbers were relatively close to each other.

**Species**	X^2	**P-value**
Serratia marcescens	18.925	<0.0001*
Klebsiella pneumoniae	0.432	0.511
Staphylococcus aureus	0.362	0.547
Escherichia coli	0.016	0.898
Klebsiella michiganensis	7.378	0.007*
Enterobacter cloacae complex	0.116	0.733
Acinetobacter baumannii	0.020	0.886
Burkholderia cenocepacia	5.199	0.023*
Ralstonia mannitolytica	1.035	0.309
Pseudomonas aeruginosa	0.151	0.698
Other species	0.087	0.768

Supplementary Table 22. Comparison of numbers of Gram-negative isolates resistant to each antibiotic assessed in the study, for BARNARDS overall MIC dataset and those within this study. MICs were representative of the overall dataset for ampicillin, amikacin and piperacillin-tazobactam. However, a higher resistance % was seen for those included in the study for gentamicin, ceftazidime and amoxicillin-clavulanate. This could have been partly due to the higher number of *S. marcescens* isolates included.

Antibiotic	% resistant isolates from BARNARDS overall with MIC data, n=883		% resistant isolates from study subset with MIC data, n=390		X^2	P-value
	Resistant	Increased exposure	Resistant	Increased exposure		
Ampicillin	95.36% (842)	-	97.17% (379)	-	2.294	0.130
Gentamicin	59.91% (529)	1.02% (9)	70.30% (274)	1.29% (5)	12.436	0.0004*
Ceftazidime	60.36% (533)	11.89% (105)	71.02% (277)	7.71% (30)	13.291	0.0003*
Amikacin	25.25% (223)	1.93% (17)	25.90% (101)	1.80% (7)	0.059	0.808
Amoxicillin-clavulanate	77.80% (687)	-	85.13% (332)	-	9.089	0.003*
Piperacillin-tazobactam	28.20% (249)	4.30% (38)	29.49 % (115)	6.15% (24)	0.220	0.639

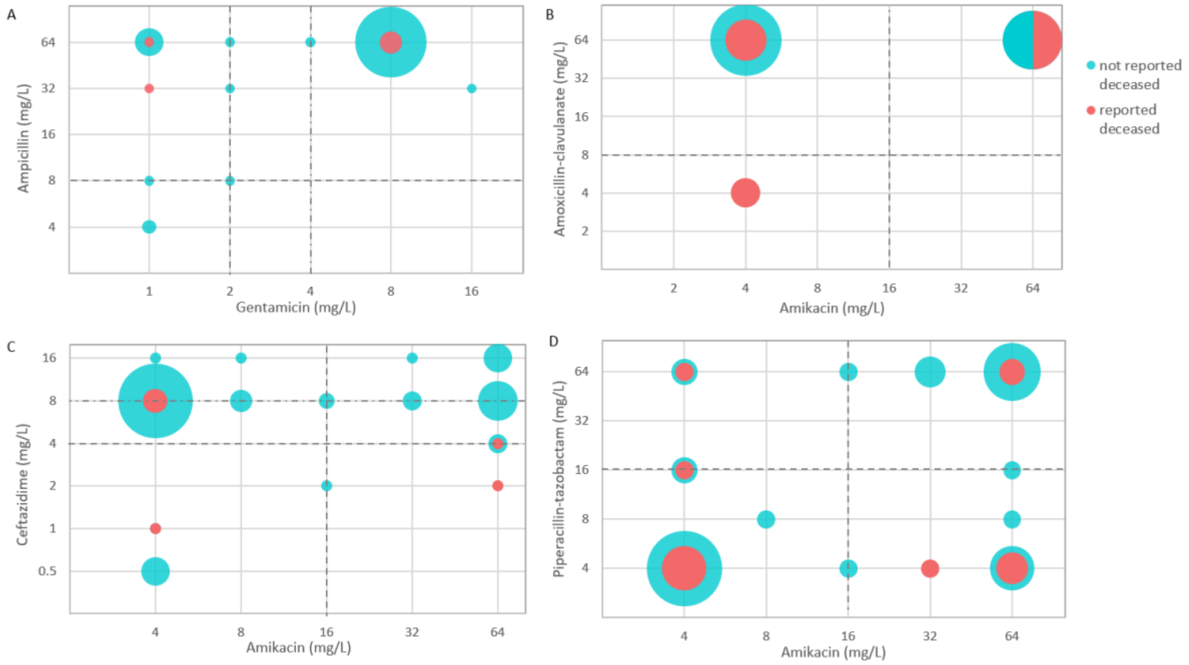

Supplementary Figure 12. MIC outcome graphs showcasing dispersion of combined MIC values for the top four most commonly applied treatment combinations with outcome for neonates only treated with each of the first empirical therapy. A: AMP-GEN, n=76 (MIC values not available for 2 isolates), B: AMC-AMK, n=24 (MIC values not available for 3 isolates), C: CTZ-AMK, n=107 (no MIC values for 2 isolates), and D: PIP/TAZ-AMK, n=76. Size represents the number of isolates with MIC value combinations. Isolates were split according to outcome after follow-up for 60 days following birth or admission into the hospital. Dotted lines represent breakpoint values according to EUCAST v9.0 (EUCAST, 2019) for Enterobacteriaceae and Pseudomonas species where relevant. MICs were tested only at concentrations around their respective breakpoints. Maximum MICs given are the concentration above the highest concentration tested. MICs are provided for non-fermenting bacteria up to 16µg/ml and Enterobacteriaceae up to 8µg/ml to cover resistance breakpoints. NB. Only non-fermenters were tested at concentration 8mg/L for Ceftazidime. Antibiotic therapies in this analysis were the primary empirical treatment given to neonates upon clinical diagnosis of sepsis. The four charts are all set to a scale of 100 and therefore the sizes of the bubbles are relative to the number of isolates included in each treatment combination.

MIC vs outcome survival curves for differing resistance profiles: resistance to both or susceptible to at least one antibiotic for each treatment combination with outcome for neonates treated only with one empirical therapy, n=290.

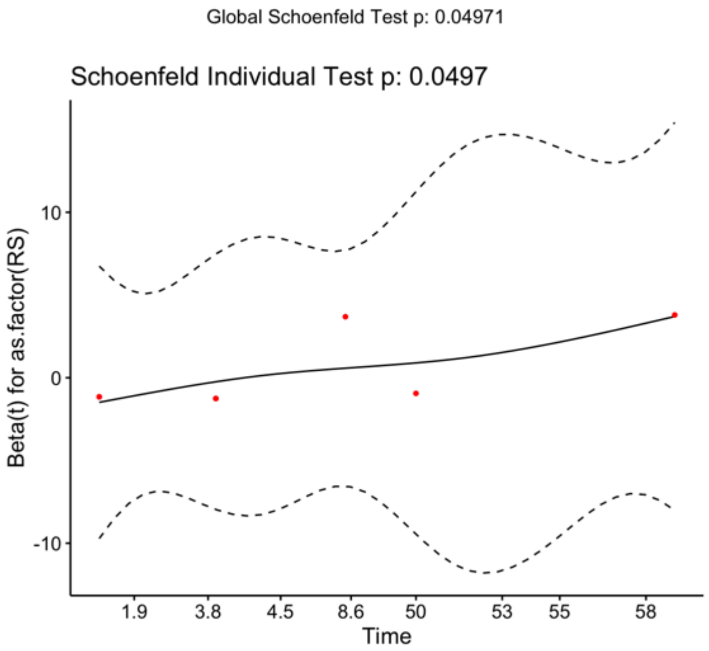

A

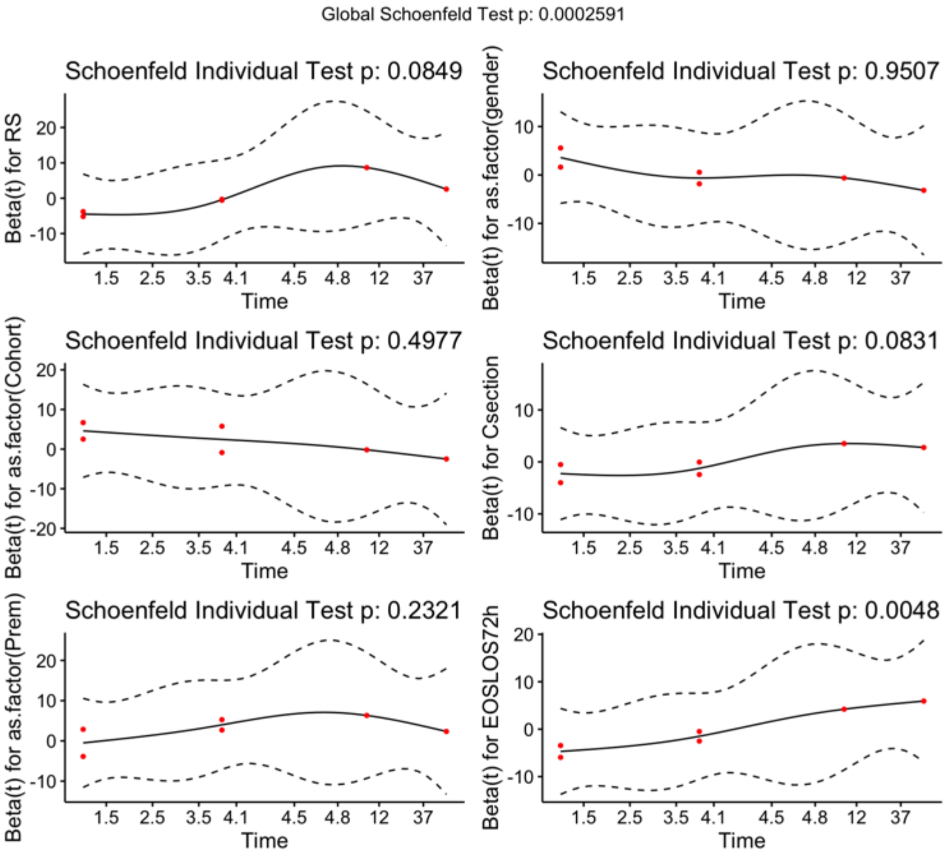

B.

Supplemental Figure 13. Schoenfeld analyses for A. unadjusted and B adjusted cox regression carried out for AMP-GEN MIC vs outcome. Small sample size for AMP-GEN with few events in this empirical only dataset. Not adjusted: 1 observation deleted due to missingness n=75, events=7. Adjusted: 8 observations deleted due to missingness n=52. Number of events=6. Type of sepsis (GNB/GNB) was removed from the adjusted model due to expansive CIs. Proportional hazard assumptions could not be met after stratification of variables while maintaining convergence (no stratification shown in B) therefore results should be read with caution as suggests a possible relationship with time.

Supplementary Table 23. Cox regression proportional hazards results for empirical dataset for neonates treated with only AMP-GEN, n=76 (MIC values not available for 2 isolates) comparing survival for those infected with pathogens resistant to both antibiotics in the combination compared to those infected with pathogens susceptible to at least one of the antibiotics prescribed. Results are given for unadjusted and adjusted models as per clinical information provided in Supplementary Table 12. Proportional hazard assumptions could not be met via stratification, therefore results for the adjusted model are to be evaluated with caution. Adjusted analysis n=52 (24 observations deleted due to missingness), number of events=6). Associated graphs are provided below.

	Unadjusted				Adjusted for clinical factors				Mixed effects model, country variation			
	HR	95% CI		P-value	HR	95% CI		P-value	HR	95% CI		P-value
		Lower	Upper			Lower	Upper					
RR												
RS	1.279	0.248	6.603	0.769	1.256	0.092	17.205	0.863	1.258	-2.386	2.845	0.860

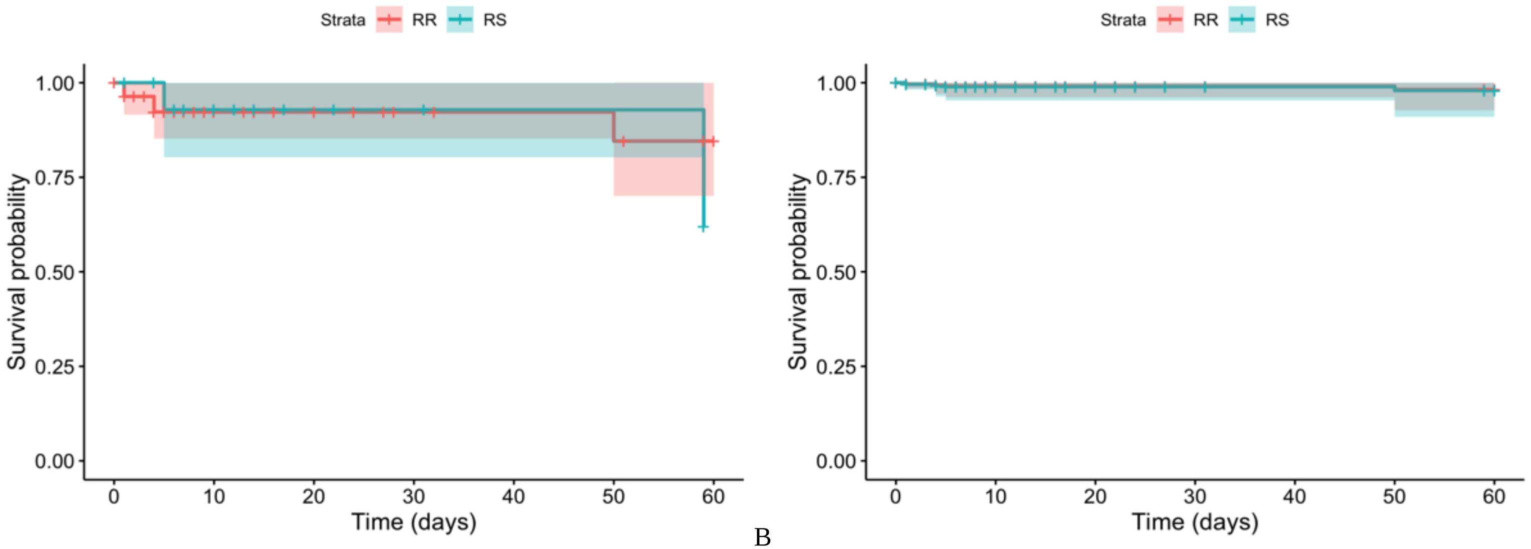

A B

Supplementary Figure 14. Cox regression proportional hazards results displayed as graphs for neonates treated only with AMP-GEN, n=76 per MIC of sepsis causing pathogen, provided for A) unadjusted and B) adjusted models as per clinical information provided in Supplementary Table 11. For adjusted graph, clinical information was set at: gender: male, cohort: inborn, Type of sepsis: EOS, sepsis pathogen type: GNB, C-section: no, premature: no. Survival curves were made in R Studio, with the survival and survminer packages.

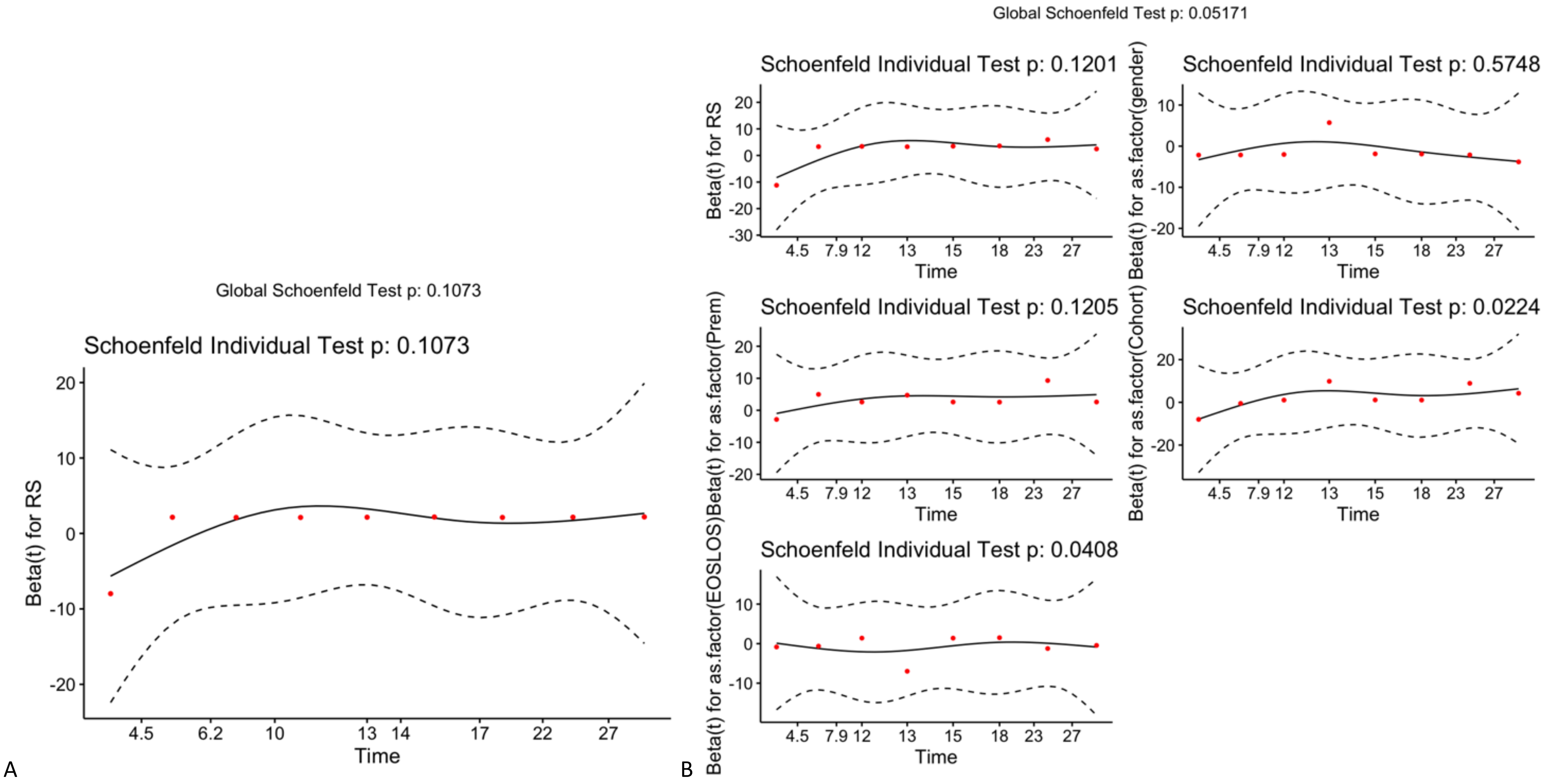

Supplementary Figure 15. Schoenfeld analyses for A. unadjusted and B adjusted cox regression carried out for CTZ-AMK MIC vs outcome. Adjusted: N=99 (8 observations deleted due to missingness), N observations=8. Type of sepsis (GNB/GPB was removed from this model due to expansive CIs and stratified by C-Section in order to meet PH assumptions.

Supplementary Table 24. Cox regression proportional hazards results for empirical dataset for neonates treated with CTZ-AMK, n=107 (MIC values not available for 2 isolates) for resistance of infecting isolate against CTZ-AMK and outcome. Results below include unadjusted and adjusted models as per clinical information provided in Supplementary Table 12. Type of sepsis (GNB/GPB) was removed from adjusted Cox regression due to expansive confidence intervals and stratified by C-section yes or no in order to meet PH assumptions. Adjusted analysis n=100 (7 observations deleted due to missing data), number of events=8. A mixed effect model and adjusted for country was not carried out as 105/107 isolates were from Bangladesh. Associated graphs provided below.

	Unadjusted				Adjusted for clinical factors			
	HR	95% CI		P-value	HR	95% CI		P-value
		Lower	Upper			Lower	Upper	
RR								
RS	2.781	0.348	22.240	0.335	5.984	0.416	86.109	0.188

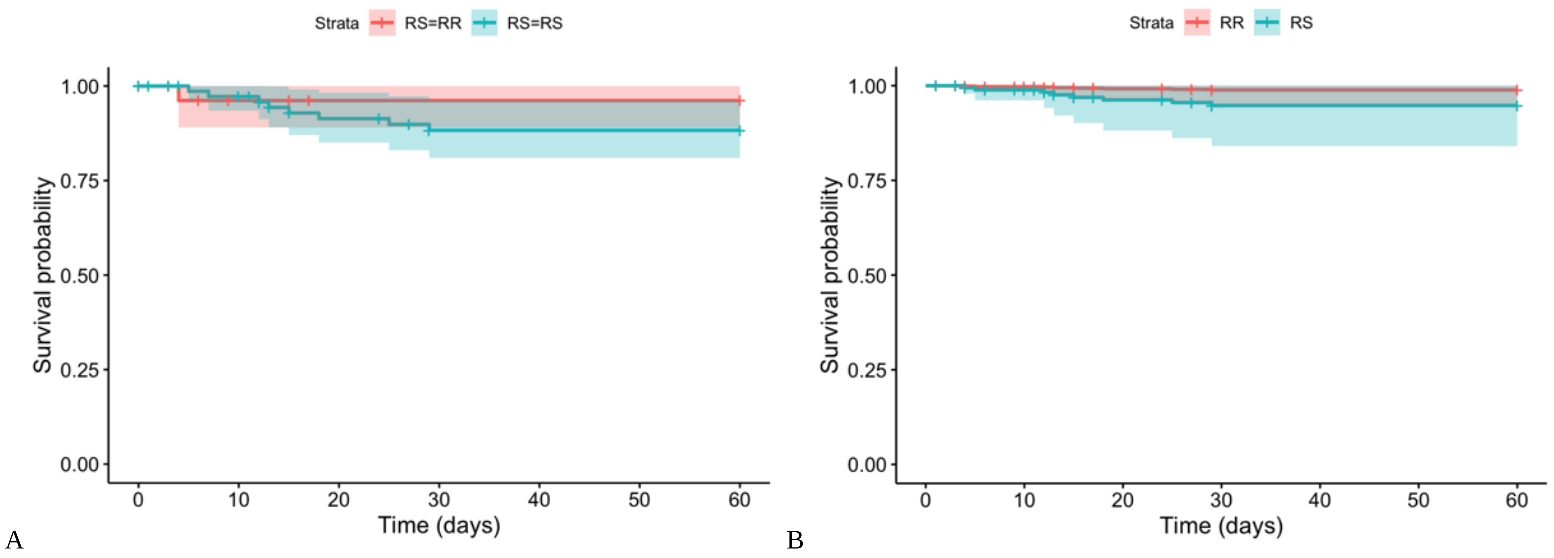

Supplementary Figure 16. Cox regression proportional hazards survival curves for neonates treated only with CTZ-AMK, per MIC of sepsis causing pathogen, provided for A) unadjusted and B) adjusted models as per clinical information provided in Supplementary Table 12. For adjusted graph, clinical information was set at: gender: male, cohort: inborn, Type of sepsis: EOS, C-section: no, premature: no. Survival curves were made in R Studio, with the survival and survminer packages.

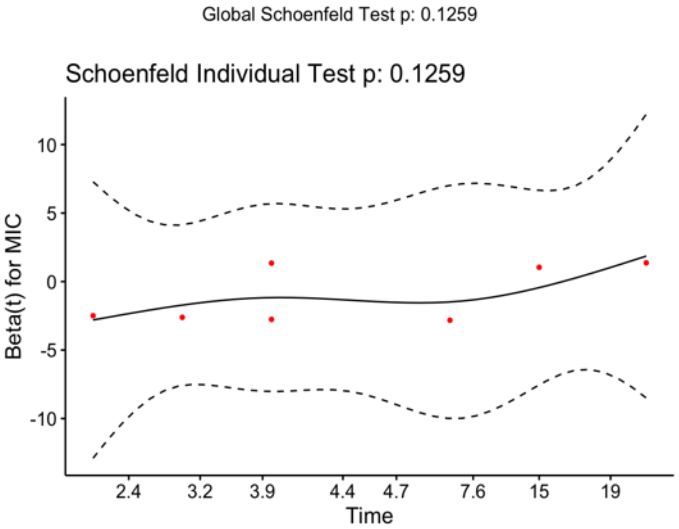

A

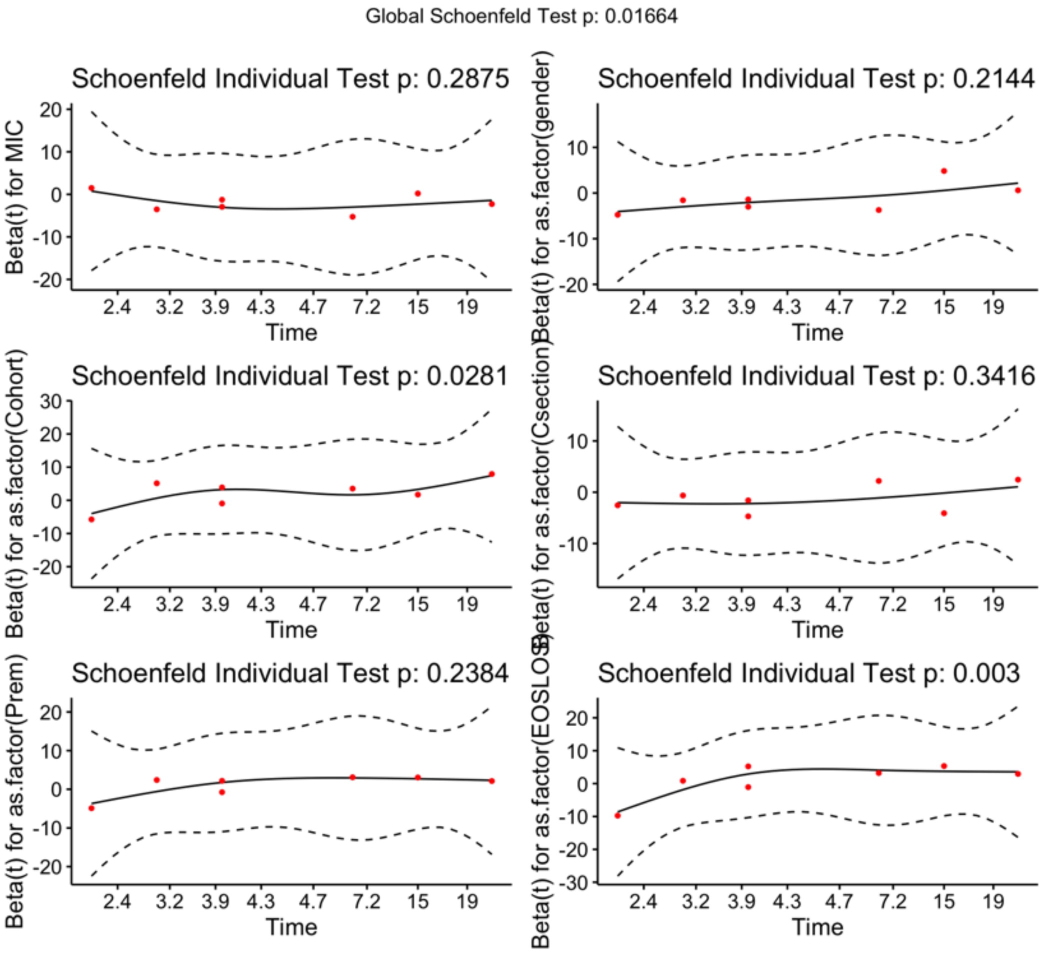

B

Supplementary Figure 17. Schoenfeld residuals tests for MIC vs outcome AMC-AMK. A significant result demonstrates significance with EOSLOS. The model could not be converged with stratification of this variable, therefore this was results are to be read with caution.

Supplementary Table 25. Cox regression proportional hazards results for empirical dataset for neonates treated with AMC-AMK, n=24 (MIC values not available for 3 isolates) for unadjusted and adjusted models as per clinical information provided in Supplementary Table 12. N=23 for adjusted analysis (1 observation deleted due to missing data, number of events=7). Final type of sepsis variable was removed from this model due to expansive CIs. Stratification could not overcome issues with proportional hazard violoations for this model, therefore adjusted results should be assessed with caution. A mixed effect model and adjusted for country was not carried out as all isolates were from Nigeria. Associated graphs provided below.

	Unadjusted				Adjusted for clinical factors			
	HR	95% CI		P-value	HR	95% CI		P-value
		Lower	Upper			Lower	Upper	
RR								
RS	0.372	0.083	1.669	0.196	0.144	0.009	2.314	0.171

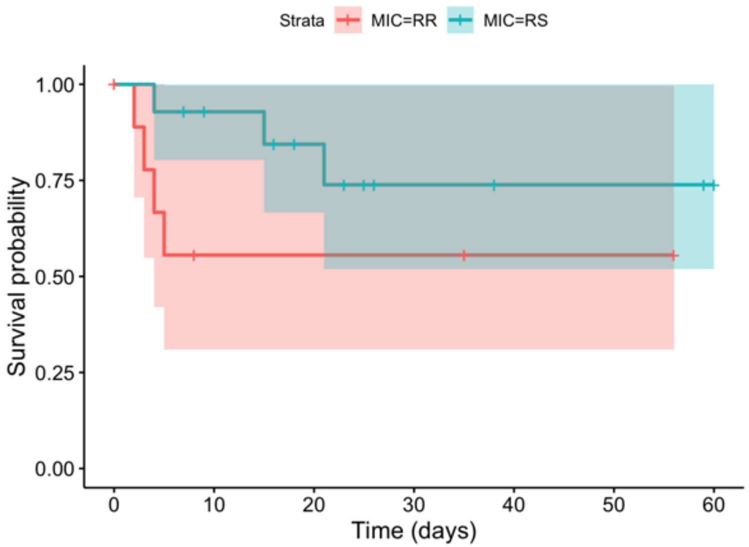

A

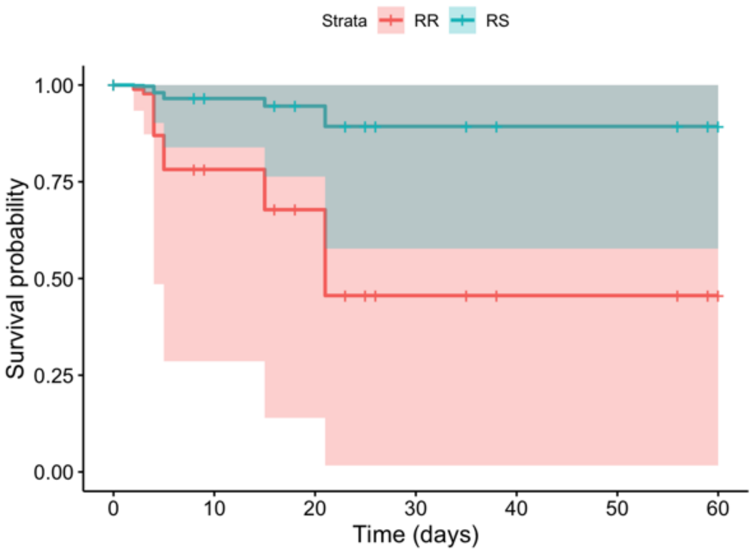

B

Supplementary Figure 18. Cox regression proportional hazards results displayed as graphs for neonates treated only with AMC-AMK, per MIC of sepsis causing pathogen, provided for A) unadjusted and B) adjusted models as per clinical information provided in Supplementary Table 12. For the adjusted graph, clinical information was set at: gender: male, cohort: inborn, Type of sepsis: EOS, sepsis pathogen type: GNB, C-section: no, premature: no. Strata 1=RR; 2=RS (No SS isolates were found in this dataset). Survival curves were made in R Studio, with the survival and survminer packages.

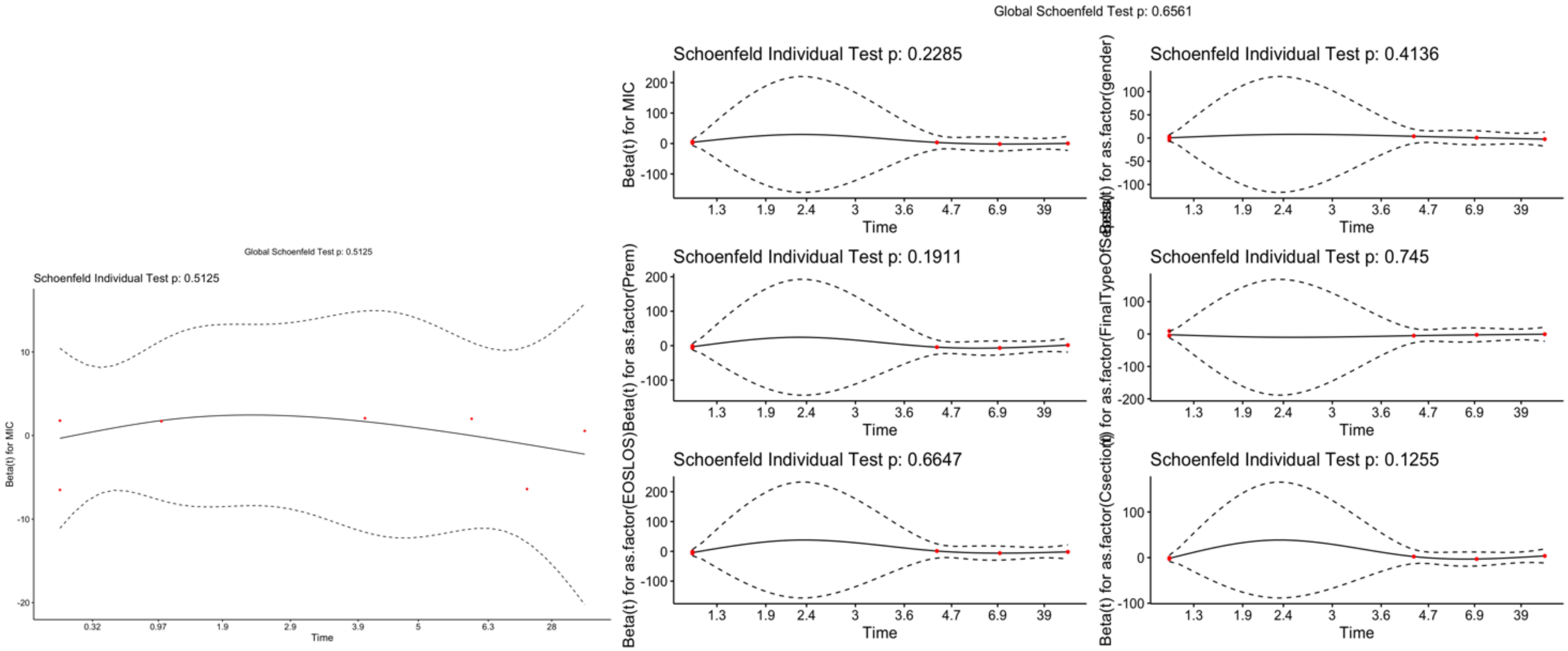

Supplementary Figure 19. Schoenfeld residuals tests for MIC vs outcome PIPT-AMK. All PH assumptions were met for these models, however, large CIs can be seen for the adjusted model.

Supplementary Table 26. Cox regression proportional hazards results for empirical dataset for neonates treated with PIP/TAZ-AMK, n=76 (MIC values not available for 3 isolates) for unadjusted and adjusted models as per clinical information provided in Supplementary Table 12. High confidence intervals were witnessed for the adjusted analysis, partly due to 47 observations deleted from analysis with missing data (n=29, number of events=8). Cohort was deleted from this model due to expansive CIs. Associated graph provided below. A mixed effect model and adjusted for country was not carried out as all isolates were from Pakistan.

	Unadjusted				Adjusted for clinical factors			
	HR	95% CI		P-value	HR	95% CI		P-value
		Lower	Upper			Lower	Upper	
RR								
RS	1.732	0.383	7.825	0.475	15.276	0.914	255.254	0.058

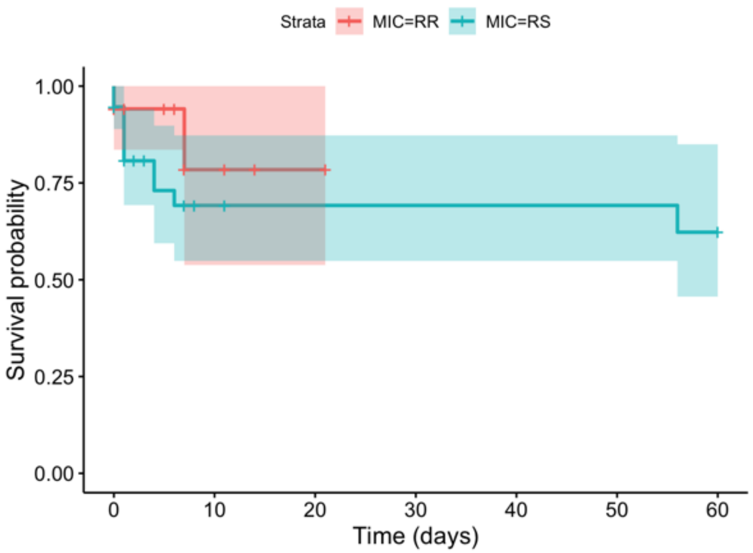

Supplementary Figure 20. Cox regression proportional hazards results displayed as graphs for neonates treated only with PIP/TAZ-AMK, per MIC of sepsis causing pathogen, provided for unadjusted cox regression analysis. Red line displaying RR isolates stops at 21 days, as this was the last observation for neonates with RR pathogens. No adjusted graph is displayed due to extremely high confidence interval, this was not easily visualised. Survival curves were made in R Studio, with the survival and survminer packages.

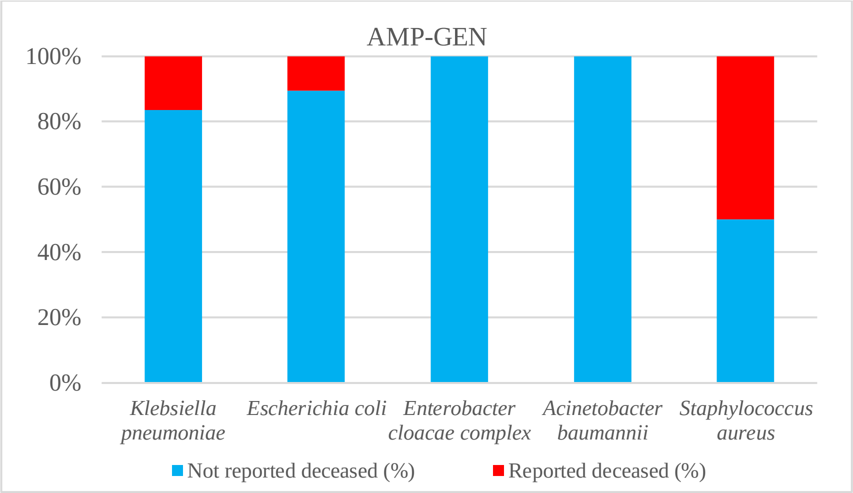
AMP-GEN
100%
80%
60%
40%
20%
0%
Klebsiella pneumoniae
Escherichia coli
Enterobacter cloacae complex
Acinetobacter baumannii
Staphylococcus aureus
Not reported deceased (%)
Reported deceased (%)

A

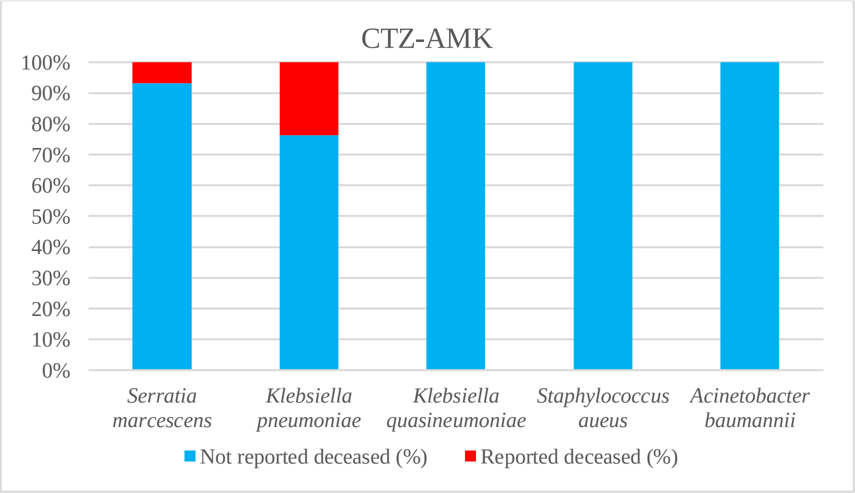
CTZ-AMK
100%
90%
80%
70%
60%
50%
40%
30%
20%
10%
0%
Serratia marcescens
Klebsiella pneumoniae
Klebsiella quasineumoniae
Staphylococcus aueus
Acinetobacter baumannii
Not reported deceased (%)
Reported deceased (%)

B

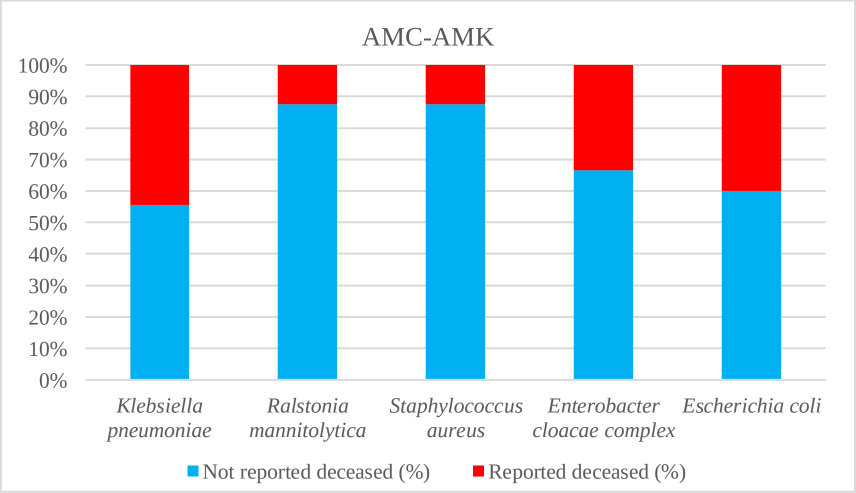
AMC-AMK
100%
90%
80%
70%
60%
50%
40%
30%
20%
10%
0%
Klebsiella pneumoniae
Ralstonia mannitolytica
Staphylococcus aureus
Enterobacter cloacae complex
Escherichia coli
Not reported deceased (%)
Reported deceased (%)

C

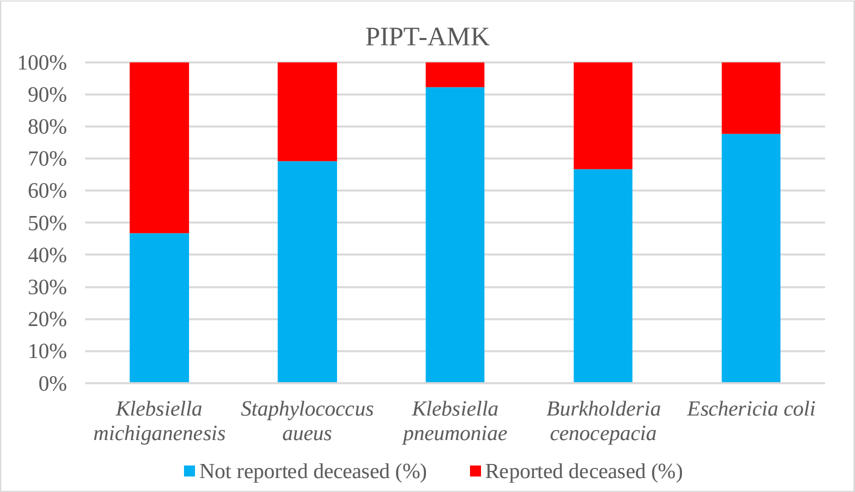

D

Supplementary Figure 21. Outcomes considered per top five species found to be sepsis causing pathogen in neonates treated by each antibiotic combination therapy. A) AMP-GEN: K. pneumoniae n=49, E. coli n=19, *E. cloacae* n=9, *A. baumannii* n=8, *S. aureus* n=8; B) CTZ-AMK: S. marcescens n=103, K. pneumoniae n=21, *K. quasipneumonia*e n=6, *S. aureus* n=6, *A. baumannii* n=6; C) AMC-AMK: *K. pneumoniae* n=27, *R. mannitolytica* n=12, S. aureus n=8, E. cloacae n=6, *E. coli* n=5; D) PIP/TAZ-AMK: *K. michiganensis* n=30, *S. aureus* n=18, *K. pneumoniae* n=13, *B. ceneocepacia* n=12, *E. cloacae* n=9. Of those infected with *S. aureus*, worst outcomes were found for treatment with AMP-GEN. The poorest outcomes for neonates infected with *K. pneumoniae* or *E. coli* were found when treated with AMC-AMK. *A. baumannii* was not associated with mortality.

Supplementary Table 27. Number of cases with top four combinations prescribed as empirical first-line treatment and number of times each therapy combination needed to be changed following original therapy. Cases where additional antibiotics were reported to be prescribed simultaneously were excluded.

Antibiotic first line treatment combination	Total number	Number of times therapy was changed from original
**Ampicillin + Gentamicin**	106	22 (22%)
**Amoxicillin + Amikacin**	61	34 (55.7%
**Piperacillin-Tazobactam + Amikacin**	83	6 (7.2%)
**Ceftazidime + Amikacin**	158	49 (31.0%)

Supplementary Table 28. Antibiotics used in treatment line of neonatal sepsis across multiple sites. This table denotes whether an antibiotic therapy change was recorded following treatment with each antibiotic/ combination. These include first line to fifth line treatment, and whether a change occurred following prescription of each antibiotic/ combination below. Antibiotics included if prescribed in >=10 cases of neonates included in this study. Cases where additional antibiotics were reported to be prescribed simultaneously were excluded.

Antibiotic prescription (singular or combination)	Total number treated with antibiotic	Number of cases reported antibiotic failure	% antibiotic failure
**Ceftazidime + Amikacin**	159	50	31.4%
**Ampicillin + Gentamicin**	113	31	27.4%
**Piperacillin-Tazobactam + Amikacin**	85	9	10.6%
**Amoxicillin (clavulanate) + Amikacin**	66	38	57.6%
**Meropenem**	26	11	42.3%
**Levofloxacin**	19	9	47.4%
**Cefotaxime**	14	3	21.4%
**Vancomycin**	12	6	50.0%
**Ciprofloxacin**	11	1	9.1%
**Meropenem + Vancomycin**	10	1	10.0%
**Meropenem + Levofloxacin**	10	0	0%

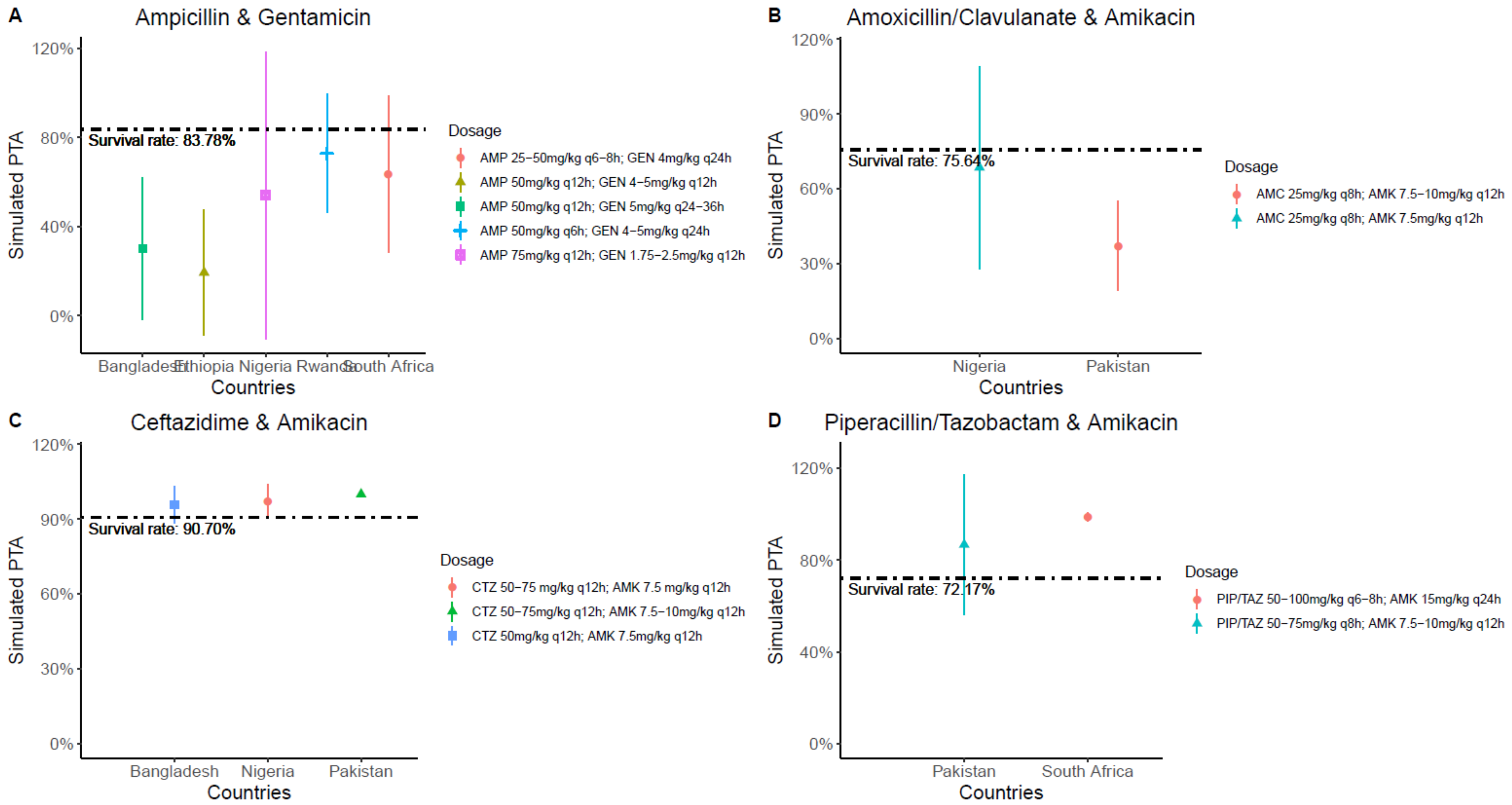

Supplementary Figure 22. Simulated PTAs for different site (country)-specific dosage regimens for the most commonly used four antibiotic combination therapies (n=476 patients).

Supplementary Table 29. Sensitivity analyses of PTA analysis. Effect of gender imputation and co-administration of dopamine and ibuprofen on PTA values.

Antibiotic combination	Impute with average	Assumption all males	Assumption of all females	T-test
	Combined PTA	Combined PTA	Combined PTA	
AMP-GEN	0.546 ± 0.390	0.548 ± 0.391	0.544 ± 0.391	P= 0.984, 0.969
AMX-AMK	0.692 ± 0.381	0.639 ± 0.402	0.709 ± 0.368	P= 0.628, 0.866
CAZ-AMK	0.951 ± 0.077	0.950 ± 0.077	0.948 ± 0.080	P = 0.898, 0.813
TAP-AMK	0.865 ± 0.308	0.864 ± 0.306	0.862 ± 0.306	P= 0.973, 0.940

Antibiotic combination	Without co-administration of Dopamine (F_DOPA=0)	With co-administration of Dopamine (F_DOPA=-0.12)	T-test
	Combined PTA	Combined PTA	
AMP-GEN	0.546 ± 0.390	0.549 ± 0.391	P = 0.968

Antibiotic combination	Without co-administration of ibuprofen (IBU=1)	With co-administration of ibuprofen (IBU=0.833)	T-test
	Combined PTA	Combined PTA	
AMX-AMK	0.692 ± 0.381	0.733 ± 0.382	P = 0.691
CAZ-AMK	0.951 ± 0.077	0.954 ± 0.076	P = 0.748
TAP-AMK	0.865 ± 0.308	0.868 ± 0.307	P = 0.951

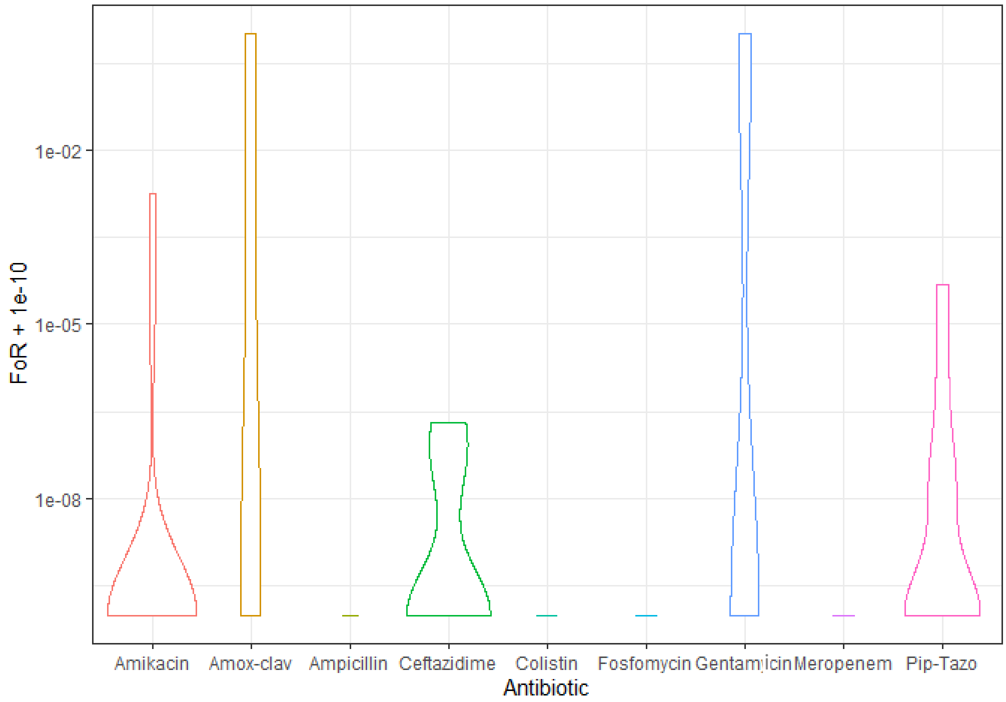

Supplementary Figure 23. Frequency of resistance occurring in *E. coli* isolates tested against Amikacin (n=19); Amoxicillin-clavulanate (n=6); Ampicillin (n=4); Ceftazidime (n=12); Colistin (n=19); Fosfomycin (n=19); Gentamicin (n=19); Meropenem (n=19) and Piperacillin-tazobactam (n=18). Data is presented per ml. Results have been log transformed with a standard of $1x10^{-10}$ added to enable incorporation of zero values. This standard was chosen, as the lowest rate of FoR found was $1x10^{-9}$.

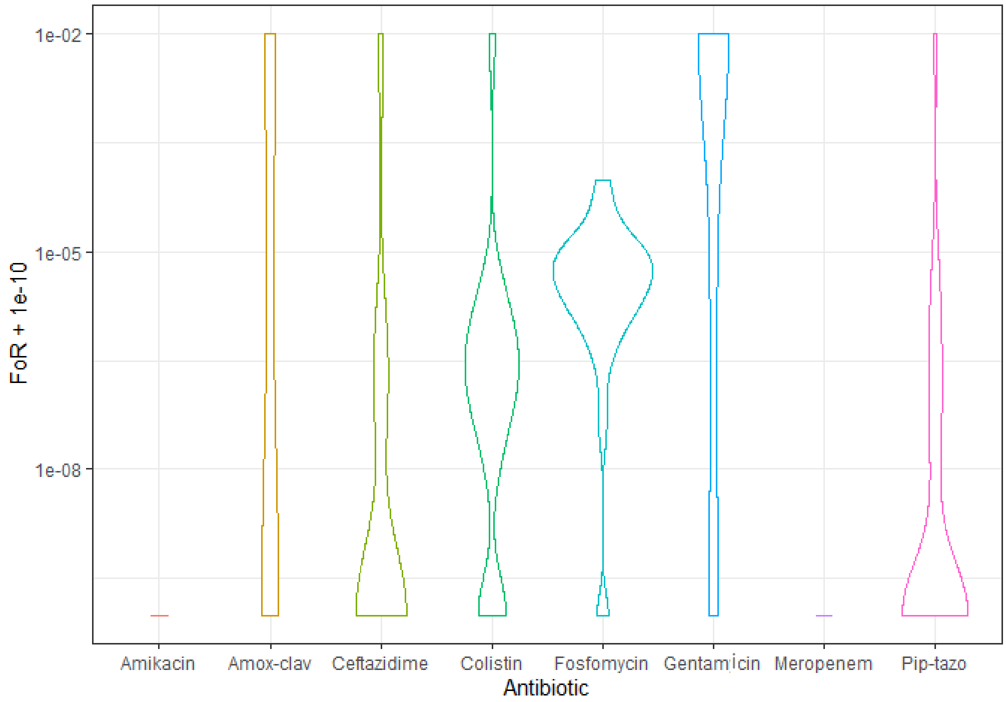

Supplementary Figure 24. Frequency of resistance occurring in *K. pneumoniae* isolates tested against Amikacin (n=52); Amoxicillin-clavulanate (n=13); Ceftazidime (n=19); Colistin (n=49); Fosfomycin (n=49); Gentamicin (n=52); Meropenem (n=52) and Piperacillin-tazobactam (n=44). No isolates were tested against Ampicillin, as *K. pneumoniae* is intrinsically resistant to this antibiotic. Data is presented per ml. Results have been log transformed with a standard of $1x10^{-10}$ added to enable incorporation of zero values. This standard was chosen, as the lowest rate of FoR found was $1x10^{-9}$.

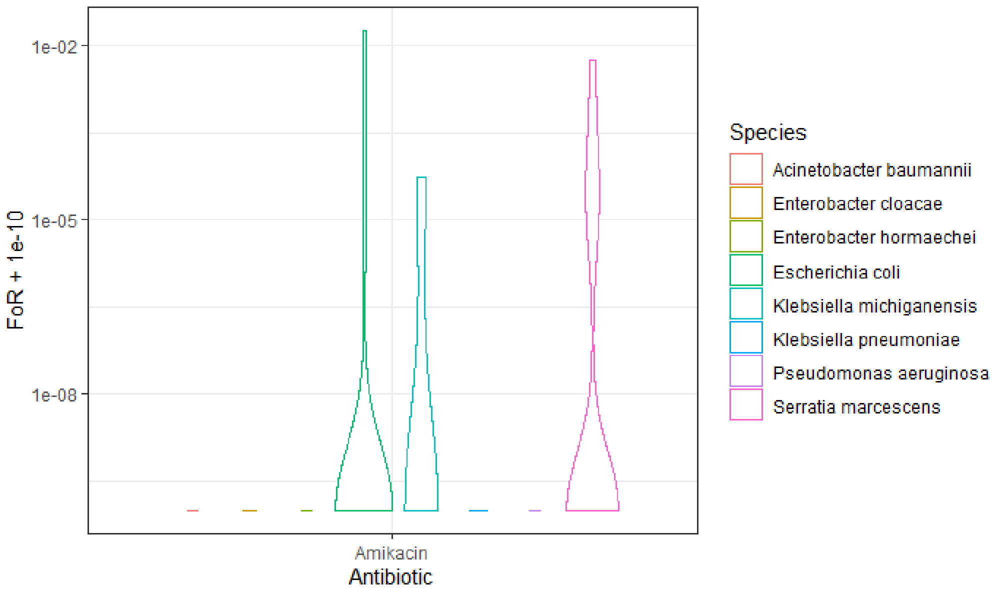
1e-02
1e-05
1e-08
FoR + 1e-10
Amikacin
Antibiotic
Species
Acinetobacter baumannii
Enterobacter cloacae
Enterobacter hormaechei
Escherichia coli
Klebsiella michiganensis
Klebsiella pneumoniae
Pseudomonas aeruginosa
Serratia marcescens

A

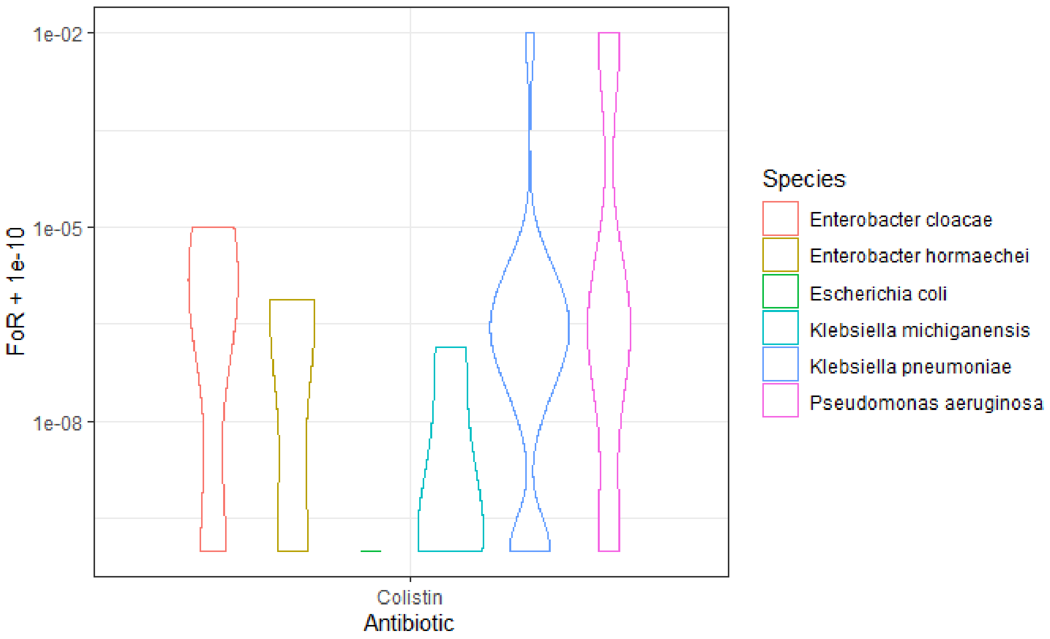
1e-02
1e-05
1e-08
FoR + 1e-10
Colistin
Antibiotic
Species
Enterobacter cloacae
Enterobacter hormaechei
Escherichia coli
Klebsiella michiganensis
Klebsiella pneumoniae
Pseudomonas aeruginosa

B

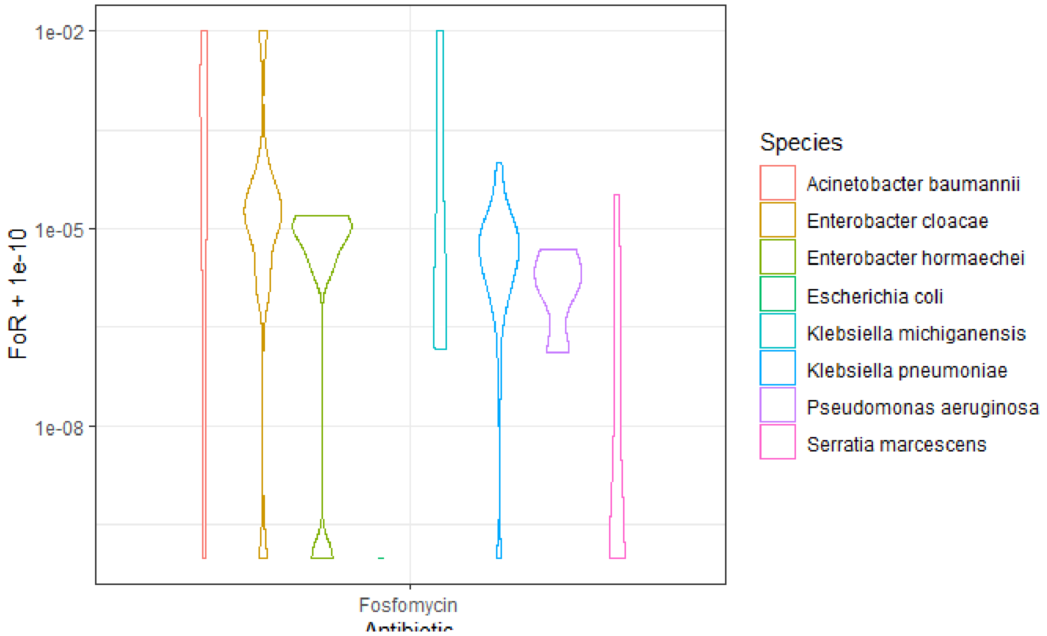
1e-02
1e-05
1e-08
FoR + 1e-10
Fosfomycin
Species
Acinetobacter baumannii
Enterobacter cloacae
Enterobacter hormaechei
Escherichia coli
Klebsiella michiganensis
Klebsiella pneumoniae
Pseudomonas aeruginosa
Serratia marcescens

C

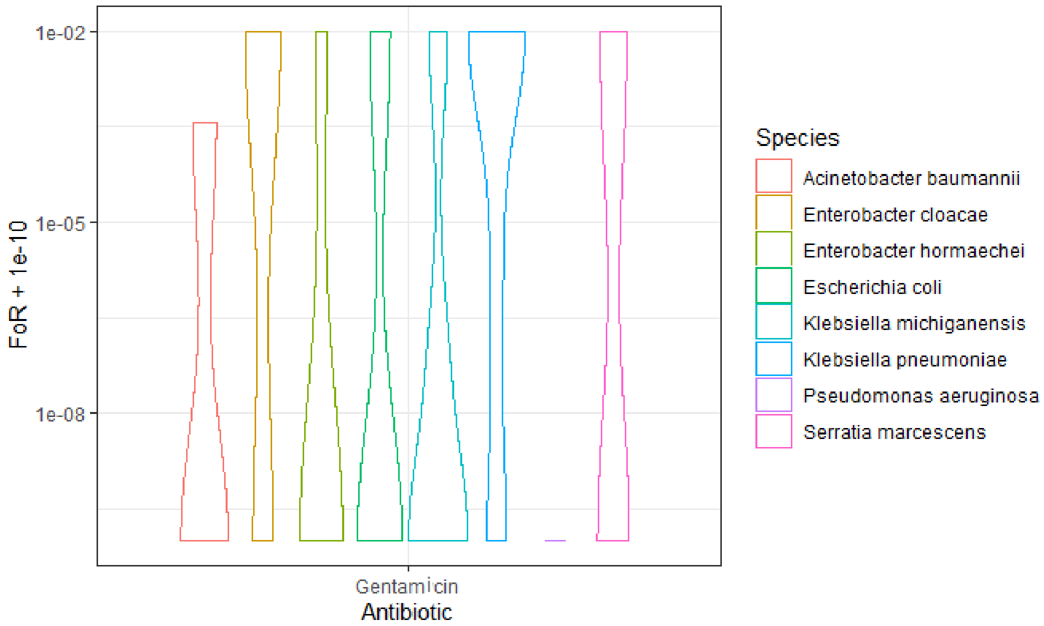
1e-02
1e-05
1e-08
FoR + 1e-10
Gentamicin
Antibiotic
Species
Acinetobacter baumannii
Enterobacter cloacae
Enterobacter hormaechei
Escherichia coli
Klebsiella michiganensis
Klebsiella pneumoniae
Pseudomonas aeruginosa
Serratia marcescens

D

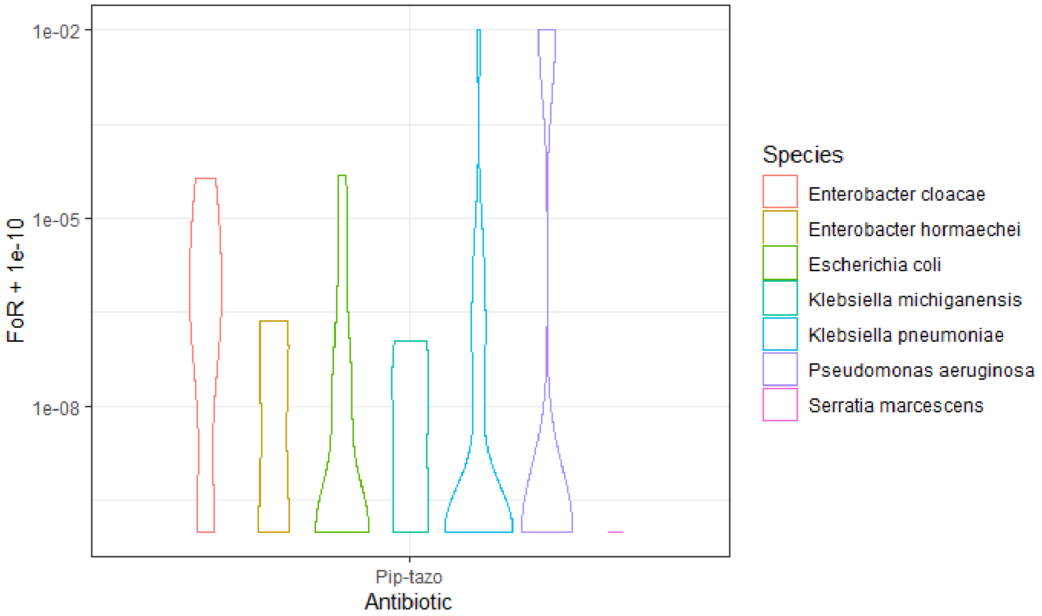

E

Supplementary Figure 25. Frequency of resistance for all Gram-negative species tested against A) Amikacin; B) Colistin; C) Fosfomycin; D) Gentamicin; E) Piperacillin-tazobactam. Results have been log transformed with a standard of $1x10^{-10}$ added to enable incorporation of zero values. This standard was chosen, as the lowest rate of FoR found was $1x10^{-9}$.

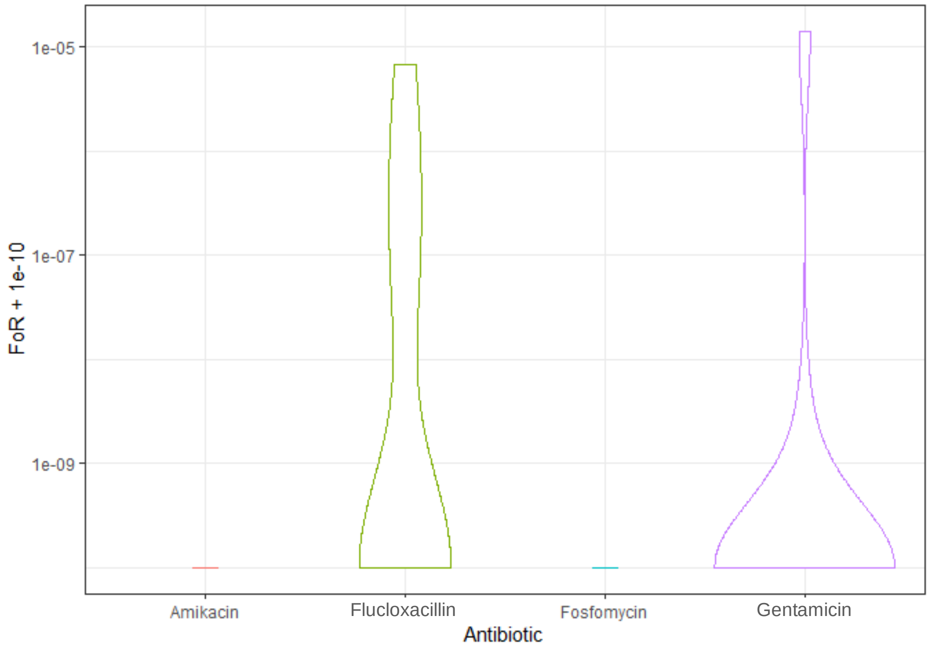

Supplementary Figure 26. Frequency of resistance (FoR) occurring in *Staphylococcus aureus* isolates tested (n=19) against amikacin, flucloxacillin, fosfomycin and gentamicin. Data is presented as growth in colony forming units (CFU) per ml. Results have been log transformed with a standard of 1x10-10 added to enable incorporation of zero values. This standard was chosen, as the lowest rate of FoR found was 1x10-9. One isolate with growth on plates supplemented with fosfomycin, one isolate on flucloxacillin supplemented agar and two on gentamicin supplemented agar were not included in this analysis, as the isolates had too much growth to determine colony forming units per ml in the neat concentration, combined with either i) too much growth at lower bacterial dilutions (fosfomycin), or ii) no growth at lower bacterial dilutions (gentamicin and flucloxacillin).

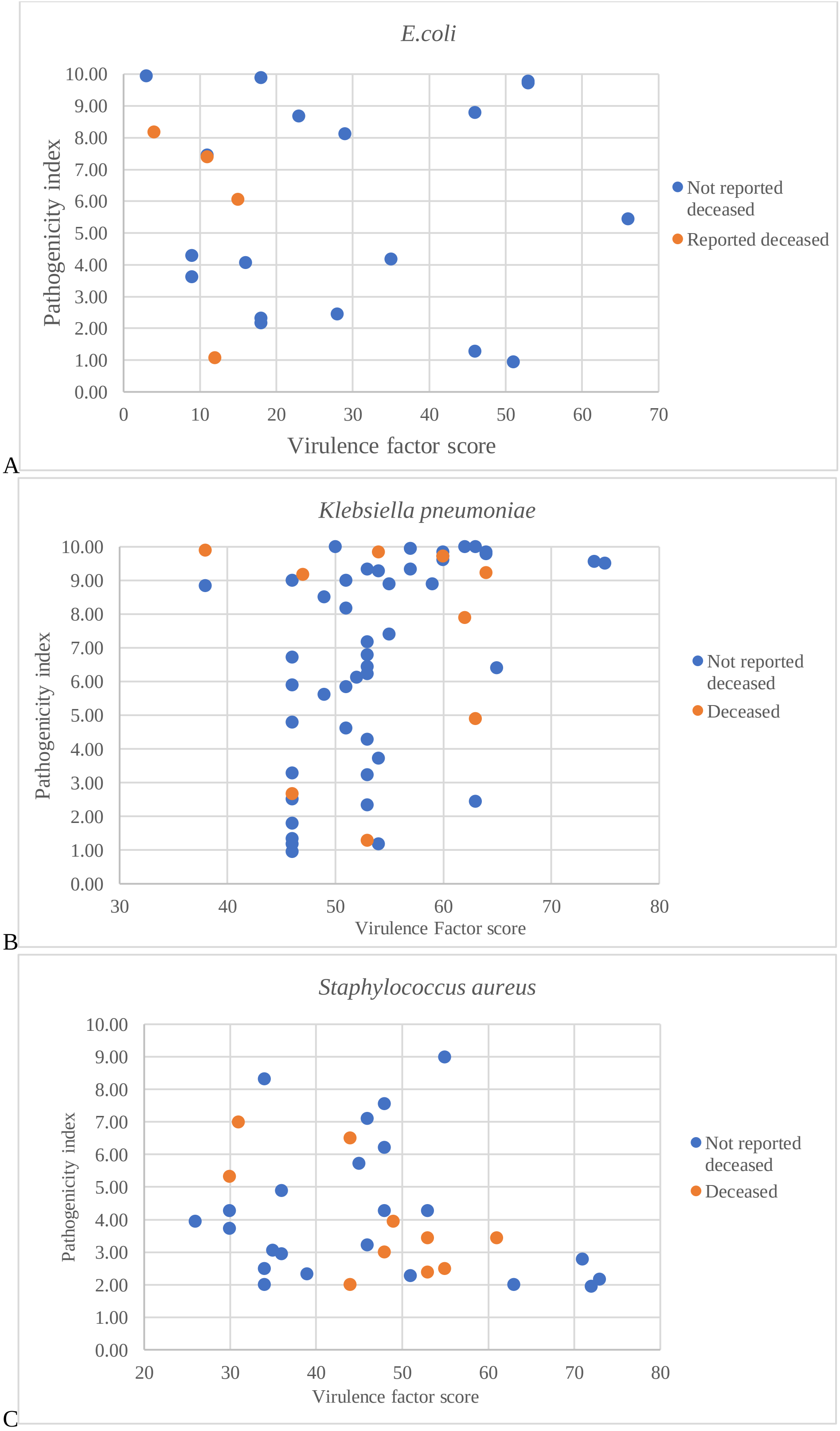

Supplementary Figure 27. Pathogencity index (PI) calculated from pathogenicity of isolates injected into *G. mellonella* models have been plotted against Virulence factor (VF) scores obtained from sequencing data for A. *E. coli* (n=24), B. *K. pneumoniae* (n=55) and C. *S. aureus* (n=34) isolates. The data was split into two groups for comparison (‘reported deceased’ and ‘not reported deceased’). Any untraceable neonates have been put into the ‘not reported deceased’ category. These bacterial species were selected for this analysis, as were found to be common causes of neonatal sepsis across BARNARDS sites and have extensive literature available on clinically relevant virulence factors.

References:

1. The European Committee on Antimicrobial Susceptibility Testing. Breakpoint tables for interpretation of MICs and zone diameters. Version 9.0, 2019. http://www.eucast.org
2. https://mic.eucast.org/Eucast2/SearchController/search.jsp?action=performSearch&BeginIndex=0&Micdif=mic&NumberIndex=50&Antib=838&Specium=-1
3. Mikolajczyk RT, Zhang J, Betran AP, *et al.* A global reference for fetal-weight and birthweight percentiles. *Lancet* 2011; **377**: 1855–61.
4. Villar J, Giuliani F, Bhutta ZA, *et al.* Postnatal growth standards for preterm infants: The Preterm Postnatal Follow-up Study of the INTERGROWTH-21stProject. *Lancet Glob Heal* 2015; **3**: e681–91.
5. WHO Child Growth Standards. *Dev Med Child Neurol* 2009; **51**: 1002–1002. https://www.who.int/childgrowth/standards/technical_report/en/
6. Boer DP, De Rijke YB, Hop WC, Cransberg K, Dorresteijn EM. Reference values for serum creatinine in children younger than 1 year of age. *Pediatr Nephrol* 2010; **25**: 2107–13.
7. Cuzzolin L, Fanos V, Pinna B, *et al.* Postnatal renal function in preterm newborns: a role of diseases, drugs and therapeutic interventions. *Pediatr Nephrol* 2006; **21**: 931–8.
8. Johnson TN, Rostami-Hodjegan A, Tucker GT. Prediction of the clearance of eleven drugs and associated variability in neonates, infants and children. *Clin Pharmacokinet* 2006; **45**: 931–56.
9. Li Z, Chen Y, Li Q, *et al.* Population pharmacokinetics of piperacillin/tazobactam in neonates and young infants. *Eur J Clin Pharmacol* 2013; **69**: 1223–33.
10. Fuchs A, Guidi M, Giannoni E, *et al.* Population pharmacokinetic study of gentamicin in a large cohort of premature and term neonates. *Br J Clin Pharmacol* 2014; **78**: 1090–101.
11. Tang BH, Wu YE, Kou C, *et al.* Population pharmacokinetics and dosing optimization of amoxicillin in neonates and young infants. *Antimicrob Agents Chemother* 2019. DOI:10.1128/AAC.02336-18.
12. De Cock RFW, Allegaert K, Schreuder MF, *et al.* Maturation of the glomerular filtration rate in neonates, as reflected by amikacin clearance. *Clin Pharmacokinet* 2012; **51**: 105–17.
13. Tremoulet A, Le J, Poindexter B, *et al.* Characterization of the population pharmacokinetics of ampicillin in neonates using an opportunistic study design. *Antimicrob Agents Chemother* 2014; **58**: 3013–20.
14. Wang H, Li X, Sun S, *et al.* Population Pharmacokinetics and Dosing Simulations of Ceftazidime in Chinese Neonates. *J Pharm Sci* 2018; **107**: 1416–22.
15. Lodise TP, Lomaestro BM, Drusano GL. Application of antimicrobial pharmacodynamic concepts into clinical practice: Focus on β-lactam antibiotics - Insights from the Society of Infectious Diseases Pharmacists. Pharmacotherapy. 2006. DOI:10.1592/phco.26.9.1320.
16. Bland CM, Pai MP, Lodise TP. Reappraisal of Contemporary Pharmacokinetic and Pharmacodynamic Principles for Informing Aminoglycoside Dosing. Pharmacotherapy. 2018. DOI:10.1002/phar.2193.
17. Smith PB, Cohen-Wolkowiez M, Castro LM, *et al.* Population pharmacokinetics of meropenem in plasma and cerebrospinal fluid of infants with suspected or complicated intra-abdominal infections. *Pediatr Infect Dis J* 2011; **30**: 844–9.
18. Du X, Li C, Kuti JL, Nightingale CH, Nicolau DP. Population pharmacokinetics and pharmacodynamics of meropenem in pediatric patients. *J Clin Pharmacol* 2006; **46**: 69–75.
19. Guggenbichler JP, Kienel G, Frisch H. [Fosfomycin, a new antibiotic drug (author's transl)]. *Padiatr Padol* 1978; **13**: 429–42936.
20. Molina MA, Olay T, Quero J. Pharmacodynamic data on fosfomycin in underweight infants during the neonatal period. *Chemotherapy* 1977; **23**: 217–22.
21. Nakwan N, Usaha S, Chokephaibulkit K, Villani P, Regazzi M, Imberti R. Pharmacokinetics of Colistin Following a Single Dose of Intravenous Colistimethate Sodium in Critically Ill Neonates. *Pediatr Infect Dis J* 2016; **35**: 1211–4.
22. Parker SL, Frantzeskaki F, Wallis SC, *et al.* Population pharmacokinetics of fosfomycin in critically ill patients. *Antimicrob Agents Chemother* 2015; **59**: 6471–6.
23. Landersdorfer CB, Nguyen TH, Lieu LT, *et al.* Substantial targeting advantage achieved by pulmonary administration of colistin methanesulfonate in a large-animal model. *Antimicrob Agents Chemother* 2017; **61**. DOI:10.1128/AAC.01934-16.
24. Traunmller F, Popovic M, Konz KH, Vavken P, Leithner A, Joukhadar C. A reappraisal of current dosing strategies for intravenous fosfomycin in children and neonates. *Clin Pharmacokinet* 2011; **50**: 493–503.
25. Nation RL, Garonzik SM, Li J, *et al.* Updated US and European Dose Recommendations for Intravenous Colistin: How Do They Perform? *Clin Infect Dis* 2016; **62**: 552–8.

26. Lepak AJ, Zhao M, Vanscoy B, *et al.* In Vivo pharmacokinetics and pharmacodynamics of ZTI-01 (fosfomycin for injection) in the neutropenic murine thigh infection model against Escherichia coli, klebsiella pneumoniae, and pseudomonas aeruginosa. *Antimicrob Agents Chemother* 2017; **61**. DOI:10.1128/AAC.00476-17.
27. Kirby WMM. Pharmacokinetics of fosfomycin. *Chemotherapy* 1977; **23**: 141–51.
28. Ooi MH, Ngu SJ, Chor YK, Li J, Landersdorfer CB, Nation RL. Population Pharmacokinetics of Intravenous Colistin in Pediatric Patients: Implications for the Selection of Dosage Regimens. *Clin Infect Dis* 2019; **69**: 1962–8.
29. Andrews, S. FastQC: a quality control tool for high throughput sequence data. http://www.bioinformatics.babraham.ac.uk/projects/fastqc (2009).
30. Krueger, F. Trimgalore. https://www.bioinformatics.babraham.ac.uk/projects/trim_galore/
31. Salzberg, S. L. FLASH : fast length adjustment of short reads to improve genome assemblies Tanja Mago c. *Bioinformatics* **27**, 2957–2963 (2011).
32. Bankevich, A. *et al.* and Its Applications to Single-Cell Sequencing. *J. Comput. Biol.* **19**, 455–477 (2012).
33. Li, H. & Durbin, R. Fast and accurate short read alignment with Burrows – Wheeler transform. *Bioinformatics* **25**, 1754–1760 (2009).
34. Li, H. *et al.* The Sequence Alignment / Map format and SAMtools. *Bioinformatics* **25**, 2078–2079 (2009).
35. Walker, B. J. *et al.* Pilon : An Integrated Tool for Comprehensive Microbial Variant Detection and Genome Assembly Improvement. *PLoS One* **9**, (2014).
36. Gurevich, A., Saveliev, V., Vyahhi, N. & Tesler, G. BIOINFORMATICS APPLICATIONS NOTE Genome analysis QUAST : quality assessment tool for genome assemblies. *Bioinformatics* **29**, 1072–1075 (2013).
37. PathogenWatch developed by Centre for Genomic Pathogen. Surveillance. https://pathogen.watch
38. Li X, Ding X, Shi P, Zhu Y, Huang Y, Li Q, Lu J, Li Z, Zhu L. Clinical features and antimicrobial susceptibility profiles of culture-proven neonatal sepsis in a tertiary children's hospital, 2013 to 2017. Medicine (Baltimore). 2019 Mar;98(12):e14686.
39. Williams PCM, Waichungo J, Gordon NC, Sharland M, Murunga S, Kamau A, Berkley JA. The potential of fosfomycin for multi-drug resistant sepsis: an analysis of in vitro activity against invasive paediatric Gram-negative bacteria. J Med Microbiol. 2019 May;68(5):711-719.
40. Tumuhamye J, Sommerfelt H, Bwanga F, Ndeezi G, Mukunya D, Napyo A, Nankabirwa V, Tumwine JK. Neonatal sepsis at Mulago national referral hospital in Uganda: Etiology, antimicrobial resistance, associated factors and case fatality risk. PLoS One. 2020 Aug 10;15(8):e0237085.
41. Gul A, Takci S. Analysis of late-onset neonatal sepsis cases in a level three neonatal intensive care unit. North Clin Istanb. 2020 May 28;7(4):354-358.
42. Shakiba T, Sadeghnia A, Karbasizade V. Detection of $bla_{\text{CTX-M15}}$ and $bla_{\text{OXA-48}}$ genes in Gram-negative isolates from neonatal sepsis in central of Iran. Iran J Microbiol. 2019 Aug;11(4):280-287. PMID: 31719958; PMCID: PMC6829106.
43. Park JW, Lee H, Park SY, Kim TH. Epidemiological, clinical, and microbiological characteristics of carbapenemase-producing Enterobacteriaceae bloodstream infection in the Republic of Korea. Antimicrob Resist Infect Control. 2019 Mar 5;8:48.
44. Leal HF, Azevedo J, Silva GEO, Amorim AML, de Roma LRC, Arraes ACP, Gouveia EL, Reis MG, Mendes AV, de Oliveira Silva M, Barberino MG, Martins IS, Reis JN. Bloodstream infections caused by multidrug-resistant gram-negative bacteria: epidemiological, clinical and microbiological features. BMC Infect Dis. 2019 Jul 11;19(1):609.
45. Sader HS, Flamm RK, Carvalhaes CG, Castanheira M. Comparison of ceftazidime-avibactam and ceftolozane-tazobactam in vitro activities when tested against gram-negative bacteria isolated from patients hospitalized with pneumonia in United States medical centers (2017-2018). Diagn Microbiol Infect Dis. 2020 Mar;96(3):114833.
46. https://www.canada.ca/content/dam/hc-sc/documents/services/drugs-health-products/canadian-antimicrobial-resistance-surveillance-system-2020-report/CARSS-2020-report-2020-eng.pdf.
47. Taher I, Almaeen A, Aljourfi H, Bohassan E, Helmy A, El-Masry E, Saleh B, Aljaber N. Surveillance of antibiotic resistance among uropathogens in Aljouf region northern Saudi Arabia. Iran J Microbiol. 2019 Dec;11(6):468-477.
48. Vu TVD, Do TTN, Rydell U, Nilsson LE, Olson L, Larsson M, Hanberger H, Choisy M, Dao TT, van Doorn HR, Nguyen VK, Nguyen VT, Wertheim HFL; VINARES consortium. Antimicrobial susceptibility testing and antibiotic consumption results from 16 hospitals in Viet Nam: The VINARES project 2012-2013. J Glob Antimicrob Resist. 2019 Sep;18:269-278.

49. Duployez C, Loïez C, Cattoen C, Wallet F, Vachée A. In vitro activity of temocillin against extended-spectrum beta-lactamase-producing Escherichia coli and Klebsiella pneumoniae strains isolated from urinary tract infections in France. Med Mal Infect. 2019 Feb;49(1):47-53.
50. Guermazi-Toumi S, Boujlel S, Assoudi M, Issaoui R, Tlili S, Hlaiem ME. Susceptibility profiles of bacteria causing urinary tract infections in Southern Tunisia. J Glob Antimicrob Resist. 2018 Mar;12:48-52.
51. Chmielarczyk A, Pobiega M, Wójkowska-Mach J, Romaniszyn D, Heczko PB, Bulanda M. Bloodstream Infections due to Enterobacteriaceae Among Neonates in Poland--Molecular Analysis of the Isolates. Pol J Microbiol. 2015;64(3):217-25.
